# Genome-wide CpG island methylation and intergenic demethylation propensities vary among different tumor sites

**DOI:** 10.1093/nar/gkv1038

**Published:** 2015-10-12

**Authors:** Seung-Tae Lee, Joseph L. Wiemels

**Affiliations:** 1 Department of Laboratory Medicine, Yonsei University College of Medicine, Seoul, 120752, Republic of Korea; 2 Department of Epidemiology and Biostatistics, University of California, San Francisco, San Francisco, California, CA 94158, USA

## Abstract

The epigenetic landscape of cancer includes both focal hypermethylation and broader hypomethylation in a genome-wide manner. By means of a comprehensive genomic analysis on 6637 tissues of 21 tumor types, we here show that the degrees of overall methylation in CpG island (CGI) and demethylation in intergenic regions, defined as ‘backbone’, largely vary among different tumors. Depending on tumor type, both CGI methylation and backbone demethylation are often associated with clinical, epidemiological and biological features such as age, sex, smoking history, anatomic location, histological type and grade, stage, molecular subtype and biological pathways. We found connections between CGI methylation and hypermutability, microsatellite instability, *IDH1* mutation, 19p gain and polycomb features, and backbone demethylation with chromosomal instability, *NSD1* and *TP53* mutations, 5q and 19p loss and long repressive domains. These broad epigenetic patterns add a new dimension to our understanding of tumor biology and its clinical implications.

## INTRODUCTION

Epigenetic alterations have pivotal roles in development and cancer biology (1,2). A canonical observation in many cancers is the *de novo* methylation of CpG islands (CGIs) in the promoters of tumor-related genes, which is significantly associated with clinical behavior in many tumors. Tumors with CGI methylation in multiple genes were often referred to as CpG island methylator phenotype (CIMP) but the definition varied among different tumors (3,4). Aside from this specific subtype, it is increasingly recognized that cancer cells show global demethylation of large intergenic and repeat regions and have large hypomethylated blocks largely overlapping across different tumor types (2,5–8). Studies on such demethylated tumors are however limited to sporadic reports on cell-lines or small patient series due to the limitation of conventional assays.

Recent genomic techniques enabled examination on such unnoticed regions. Whole genome bisulfite sequencing (WGBS) will be the best method to investigate the intergenic region; however, its utility is hampered by high cost and analytical burden. DNA methylation arrays can be a good alternative to investigate genome-wide methylation among a large collection of tumors. Such arrays have probes densely positioned for CGI and promoter regions but also have probes sparsely embedded for intergenic regions, and therefore, the comprehensive methylome can be investigated through a careful statistical approach.

This study was motivated and expanded from our previous work on B-cell acute lymphoblastic leukemias (7), in which WGBS and methylation array analyses indicated two-track methylation changes for ‘potentially functioning’ sites including CGIs, CGI shores, promoters, 5′-bodies, exons, DNase hypersensitive sites (HS), transcription factor (TF)-binding sites and enhancer sites which behave differently from other ‘relatively non-functioning’ intergenic sites. For example, many CGIs and DNase HS were *de novo* methylated in sharp contrast to the frequent demethylation of intergenic regions. TF-binding sites behaved differently according to TF contents; the binding sites of embryonic stem cell (ESC)-related TFs including polycomb proteins and CTBP2 were frequently *de novo* methylated while the binding sites of other differentiation-associated TFs were rather demethylated or unchanged. In this regard, we partitioned such regions and defined the ‘backbone’ as the remainder of the genome corresponding to neither of those functional sites nor repeat sequences; such repeat sequences were also excluded because of observed technical and statistical problems. As the backbone may represent the non-functional sites where the vast majority of human CpGs are located, we could assess genome-wide demethylation in tumor cells in comparison with the *de novo* methylation in small functional sites, especially in CGIs. The partitioning and averaging also enabled us to overcome certain biases from the unevenness of array probe design.

Adopting this strategy, we analyzed 6637 tumors in 21 tumor categories from the Cancer Genome Atlas (TCGA) project. The two-track epigenetic changes, represented as CGI methylation and backbone demethylation, were evident in most tumors but the degrees varied among different tumors. More importantly, the degrees of abnormal methylation correlated with certain biological and clinical characteristics. Such associations, especially regarding demethylation, are as yet largely unexplored and thus the current study may provide insights to biologists and clinicians interested in each tumor type.

## MATERIALS AND METHODS

### Subjects and tumor types

Genomic data and clinical information of 6637 tumor and 700 adjacent normal tissues of 21 tumor types was retrieved from the TCGA data portal (Supplementary Table S1). The 21 tumor types include adrenocortical carcinoma (ACC), bladder urothelial carcinoma (BLCA), breast invasive carcinoma (BRCA), cervical squamous cell carcinoma and endocervical adenocarcinoma (CESC), colorectal adenocarcinoma (COADREAD), glioblastoma multiforme (GBM), head and neck squamous cell carcinoma (HNSC), kidney chromophobe (KICH), kidney renal clear cell carcinoma (KIRC), kidney renal papillary cell carcinoma (KIRP), acute myeloid leukemia (LAML), brain lower grade glioma (LGG), liver hepatocellular carcinoma (LIHC), lung adenocarcinoma (LUAD), lung squamous cell carcinoma (LUSC), prostate adenocarcinoma (PRAD), skin cutaneous melanoma (SKCM), stomach adenocarcinoma (STAD), thyroid carcinoma (THCA), uterine corpus endometrial carcinoma (UCEC) and uterine carcinosarcoma (UCS).

### Retrieval and processing of methylation data

For methylation, we obtained level 2 data of the Infinium HumanMethylation450 array (Illumina) containing background-corrected methylated and unmethylated summary intensities and beta values as extracted by the methylumi package. Type 1 and 2 probe adjustment was done using the beta-mixture quantile normalization (BMIQ) model implemented in the *R* software ChAMP package. A supervised batch correction using the ComBat algorithm was done by integrating all the 6637 methylation array data and incorporating batch and array ID information. Probes having a common single nucleotide polymorphism (SNP) (minor allele frequency > 1% as defined by the UCSC snp135common track) within 10 bp of the interrogated CpG site or having 15 bp from the interrogated CpG site overlap with a repeat element (as defined by the RepeatMasker) were masked across all samples, and probes with a non-detection probability (*P*-value) greater than 0.05 in a given sample are also masked. CpGs in sex chromosomes were excluded. We also downloaded WGBS data of 40 samples from the TCGA data portal, and 14 among them had methylation array data.

### Downloading and processing other genomic data

For other genomic data, normalized gene expression (RSEM) values from RNA sequencing, microRNA (miRNA) expression values from miRNA sequencing, calculated segmentation values from the Genome-Wide Human SNP 6.0 array (Affymetrix), and validated mutation calls from exome, RNA and whole genome sequencing were obtained from the Broad Genome Data Analysis Center (GDAC) Firehose server as processed and normalized by its pre-established pipeline (version 04–16–2014; doi:10.7908/C16W9975) (Supplementary Table S1). Secondary analysis data were all downloaded from the Broad GDAC Firehose server (version 04–16–2014), including clustering information of DNA methylation, RNA expression, miRNA expression and somatic copy number alteration (SCNA) by the cNMF algorithm (best clusters by the cNMF algorithm were adopted for further analysis), significantly-mutated genes by the MutSig algorithm, significant SCNA peaks and arm- and gene-level copy changes by the GISTIC algorithm, and pathway activities inferred from RNA expression and SCNA data by the PARADIGM algorithm. Using the SCNA segmentation data downloaded from the GDAC Firehose server, we further calculated tumor purity and ploidy estimates by the ABSOLUTE software package (9).

### Region definition and calculation

Annotations on CGIs, reference genes (RefSeq gene), exons, DNase hypersensitive site clusters, consensus binding sites of 161 TFs from the chromatin-immunoprecipitation data by the ENCODE project, Vista enhancer sites, and repeat regions (defined by RepeatMasker) were downloaded from the USCS genome server. CGI shore was defined as ± 2 kb region from the CGI boundary. Promoter was defined as 0–1.5 kb upstream region of transcription start site and 5′-body was defined as 0–0.1 in fractional region of gene body. We excluded all these defined regions and designated the remainder region as ‘backbone’. Lamina-associated domains (LADs) of lung fibroblasts and FSU Repli-chip data of eight cell-lines were also downloaded from the USCS genome server. Information on partially methylated domains (PMDs) of a colon cancer cell-line and placenta, and LADs of cultured B-lymphoblasts was obtained from previous studies (10–12). Definition on 199 chromatin-modifier genes was introduced from a curated list by Gonzalez-Perez *et al*. (13).

### Clinical and pathological parameters

Clinical and pathological parameters analyzed in each tumor type are listed in Supplementary Table S2. All gradable variables including stage, histological grade, immunohistochemical stain intensity, Gleason score, Karnofsky score and ECOG performance grade were converted to numeric factors for further analysis. Smoking history graded by pack/year, anatomical locations ordered according to digestive and airway tracts, microsatellite instability (MSI) graded as microsatellite-stable (MSS), intermediate (MSI-I) and high (MSI-H), and primary treatment response graded as complete response, partial response, stable disease and progressive disease were also converted into numeric factors. Categorical variables were coded as dummy variables. A multivariate linear model controlling age and gender was used wherever applicable. A Kaplan-Meier survival analysis was performed to assess implication on clinical outcome. By reviewing literature and considering the availability of information in TCGA data set, clinical, pathological and molecular variables having well-known impact or being repeatedly suggested in previous studies were included in a multivariate Cox proportional hazards regression model in each tumor type.

### Molecular parameters

DNA methylation, RNA expression, miRNA expression and SCNA clusters were identified by the cNMF algorithm in the Firehose GDAC server and coded as dummy variables. Significantly mutated genes identified by the MutSig algorithm (Supplementary Table S3) were selected and the presence and absence of mutation in each gene was coded as binomial variables for further analysis. For copy number analysis, log2ratios of 24 174 genes, 39 arms of autosomal chromosomes, and significant chromosomal loci in each tumor type were identified by the GISTIC algorithm (Supplementary Tables S4 and S5) and coded as continuous variables. Association of molecular parameters with average CGI and backbone methylation levels were analyzed by the Wilcoxon rank-sum test for binomials, Kruskal-Wallis test for categorical variables, and Kendall rank correlation test for continuous variables. In every analysis, Benjamini-Hochberg correction by the number of comparisons (e.g. 24 174 for gene-level SCNA association) was performed.

### Differentially methylated CpGs and TF enrichment

For 12 tumor types with sufficient number of normal tissue data, we analyzed differentially methylated CpGs (defined by methylation change >0.2 compared to mean of normal tissues) in each sample. Using the 161 ENCODE TF binding sites, enrichment rates were calculated for each TF using the following equation:

\begin{equation*} \begin{array}{*{20}l} {Enrichment\;rate = } \\ {\frac{{({\rm n}.\;{\rm of}\;{\rm differentially}\;{\rm methylated}\;{\rm CpGs}\;{\rm inside}\;{\rm TF}\;{\rm sites})/({\rm n}{\rm .}\;{\rm of}\;{\rm CpGs}\;{\rm inside}\;{\rm TF}\;{\rm sites})}}{{({\rm n}.\;{\rm of}\;{\rm differentially}\;{\rm methylated}\;{\rm CpGs}\;{\rm outside}\;{\rm TF}\;{\rm sites})/({\rm n}{\rm .}\;{\rm of}\;{\rm CpGs}\;{\rm outside}\;{\rm TF}\;{\rm sites})}}} \\ \end{array} \end{equation*}

## RESULTS

### Characterization of genome-wide average methylation

The subjects comprise 6637 tumor and 700 adjacent normal tissues with Illumina 450k methylation array data available from the TCGA database (Supplementary Table S1). After filtering out sex chromosome CpGs and redundant CpGs around SNP and repeat regions, we selected a total of 136 186 CpGs in CGI and averaged their methylation levels for each tumor. Likewise, we selected 49 277 CpGs in backbone, as defined above, and averaged their methylation levels for each tumor. To confirm the validity of our definition on backbone and calculations of average methylation, we compared averages calculated by array with averages calculated by WGBS from 14 subjects from TCGA and found a good correlation (*r* = 0.840). We further estimated tumor purities by the ABSOLUTE algorithm and found that the methylation changes are not strongly affected by purity biases (Supplementary Figure S1).

**Figure 1.**
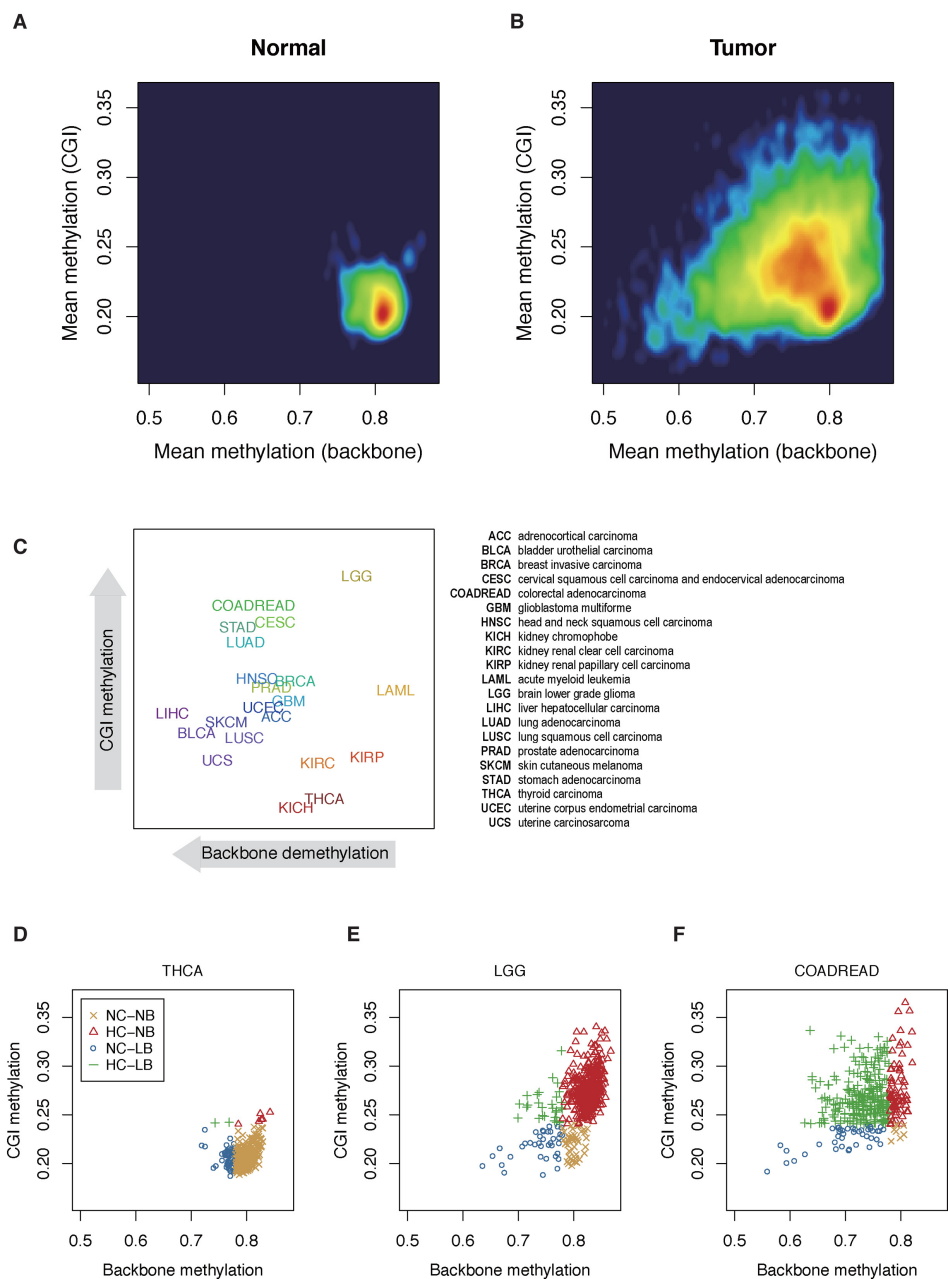
Distribution of genome-wide average CGI and backbone methylation in normal and tumor tissues. (**A**) Normal adjacent tissues have average CGI methylation around 0.2 and average backbone methylation around 0.8 within narrow limits. (**B**) Tumor cells have variable degrees of CGI methylation and backbone demethylation. (**C**) The distributions of abnormal methylations according to tumor types. Tumor names are presented at median levels of CGI and backbone methylation in each tumor type. (**D**) THCA cases are distributed mostly in normal ranges (NC-NB). Abbreviations: NC, normal CGI methylation (≤0.24); HC, high CGI methylation (>0.24); NB, normal backbone methylation (≥0.78); LB, low backbone methylation (<0.78). (**E**) LGG cases are distributed mostly in the CGI-methylated zone (HC-LB). (**F**) Most of COADREAD cases are both CGI-methylated and backbone-demethylated with variable degrees.

Figure 1 is a density plot generated by the average methylation of all CGI CpGs (*y*-axis) and average methylation of all backbone CpGs (*x*-axis) in each sample. Normal tissues maintained their CGIs hypomethylated and backbones hypermethylated within narrow ranges (0.18–0.24 and 0.78–0.82, respectively) whereas tumor cells displayed variable degrees of CGI methylation and backbone demethylation (Figure 1A and B; Supplementary Figure S2). Considering the normal ranges, we defined high CGI methylation (HC; average CGI methylation >0.24), as opposed to normal CGI methylation (NC; average CGI methylation ≤0.24), and low backbone methylation (LB; average backbone methylation <0.78), as opposed to normal backbone methylation (NB; average backbone methylation ≥0.78). In Figure 1C, the location of the tumor name represents the median of cases in each tumor type, showing a wide variety of case distribution according to tumor type. The vast majorities of THCA, KICH, KIRP and KIRC cases maintained CGI and backbone within normal methylation ranges (NC-NB; Figure 1D) whereas many LGG tumors showed deviation to CGI methylation (HC-NB; Figure 1E). Nonetheless, most tumors showed both CGI methylation and backbone demethylation with variable degrees (HC-LB; Figure 1F; Supplementary Figure S2). Medians of BLCA, UCS and LIHC were more skewed to backbone demethylation.

### Tumor-specific association

Both CGI and backbone methylation correlated very significantly with DNA methylation clusters supporting the validity of our approach. And in most tumors, DNA methylation correlated with mRNA expression, miRNA expression, copy number and pathway clusters suggesting an underlying biological background. Among the epidemiological, clinical, pathological and molecular parameters analyzed (Supplementary Tables S2–S5), those showing significant associations are exemplified in Figure 2 and outlined in more detail in Supplementary Figures S4–S24; presentation order is according to the deviation from normal zone (NC-NB, HC-NB and HC-LB) in Figure 1C.

**Figure 2.**
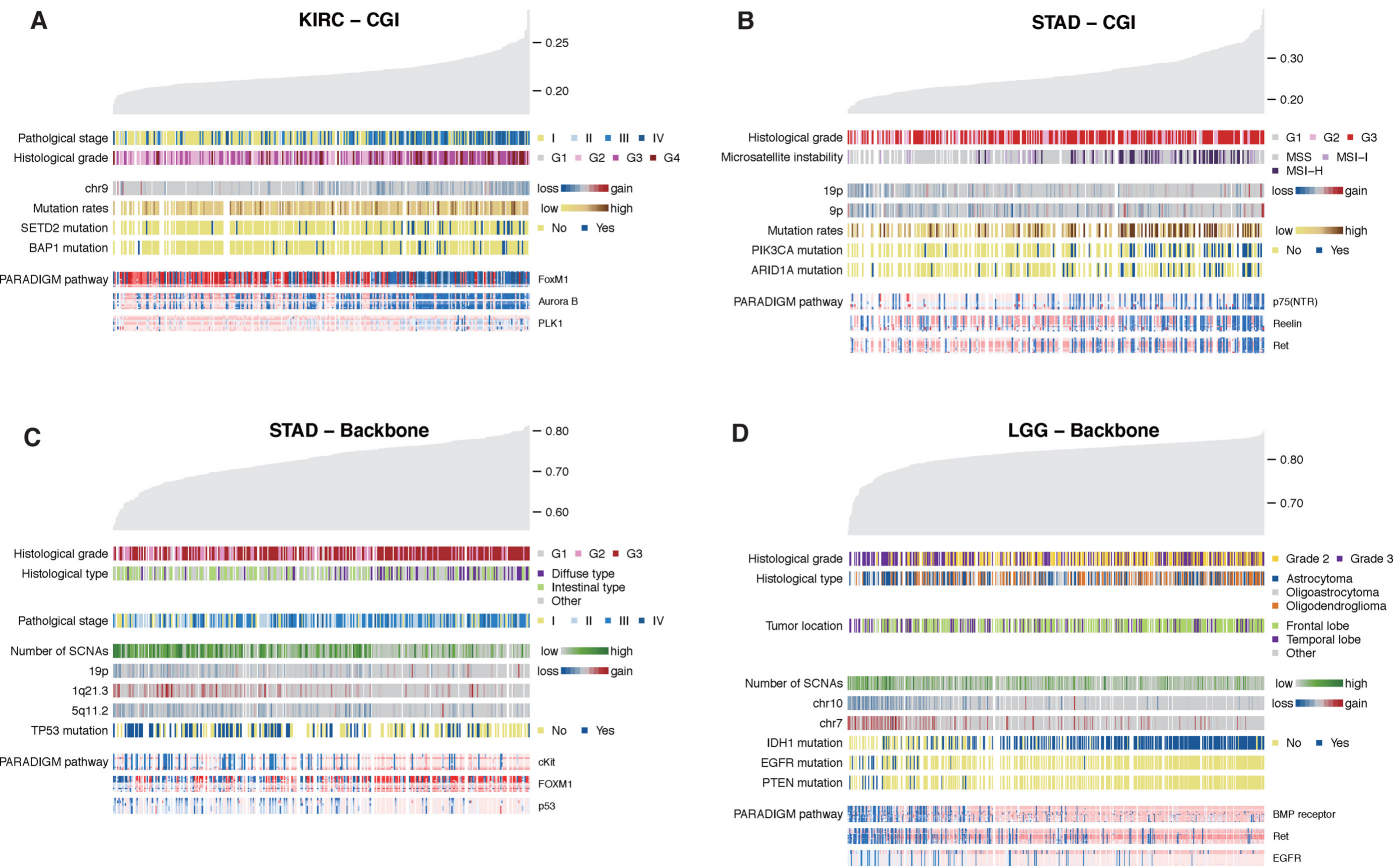
Exemplary tumors showing remarkable association with clinical, pathological and molecular parameters. (**A**) KICH tumors higher CGI methylation are related with advanced pathological stage, high grade, chromosome 9 loss, high mutation rate, *SETD2* and *BAP1* mutations, and low activities of FoxM1, Aurora B and PLK1 pathways. (**B**) STAD tumors with higher CGI methylation are related with high histological grade, high microsatellite instability (MSI-H), gains of 9p and 19p, high mutation rate, *PIK3CA* and *ARID1A* mutations, and low activities of p75^NTR^, Reelin and Ret pathways. (**C**) STAD tumors with lower backbone methylation are associated with low histological grade, non-diffuse type, low pathological stage, higher number of somatic copy number alterations (SCNAs), loss of 19p and 5q11, gain of 1q21, *TP53* mutation, and low activities of c-Kit, FoxM1 and p53 pathways. (**D**) In LGG, backbone demethylation is associated high histological grade, astrocytoma histology, temporal lobe location, high number of SCNAs, loss of chromosome 10, gain of chromosome 7, *EGFR* and *PTEN* mutations, and low activities of BMP receptor, Ret and EGFR pathways.

### Epidemiology

The CGI and backbone methylations correlated with age (*r* = +0.064 and –0.230, respectively), with different degrees according to tumor type (Supplementary Figure S3). The methylation levels also had a slight correlation with gender in a set of tumors (Supplementary Table S6). Accordingly, we controlled age and gender in the following analyses on clinical and pathological parameters.

Smoking history was significantly associated with backbone demethylation in LUAD with current smokers being the most demethylated (*P* = 1.4 × 10^−3^). In THCA, histories of lymphocytic thyroiditis significantly correlated with high CGI methylation (*P* = 6.3 × 10^−4^). In STAD, those with very high CGI methylation were not MSI-H (Figure 2B) and supposed to be the Epstein-Barr virus (EBV) type as suggested by a previous TCGA study (14).

### Anatomic location

In COADREAD, CGI methylation was highest in cecum tumors and became modest when moving towards the rectum (*P* = 1.3 × 10^−16^; Supplementary Figure S25A). In HNSC, CGI methylation was highest in the oral cavity and lower in the caudal direction in the oropharangeal tract (*P* = 8.9 × 10^−5^; Supplementary Figure S25B). In LGG, higher CGI methylation was observed in frontal lobe tumors and lower backbone methylation in temporal lobe tumors (*P* = 5.1 × 10^−6^ and 1.1 × 10^−3^, respectively).

### Histology and stage

Methylation status often correlated with histological subtypes in part reflecting their difference in cell composition. Follicular THCA had slightly higher CGI methylation than papillary THCA (*P* = 2.3 × 10^−3^). Undifferentiated LAML and LAML without maturation (M0 and M1 by the FAB classification, respectively) had higher CGI methylation (*P* = 4.5 × 10^−4^), and monocytic LAML (M5) had lower backbone methylation (*P* = 1.2 × 10^−5^). In LGG, oligodendrogliomas displayed very high CGI methylation while astrocytomas showed backbone demethylation (*P* = 2.2 × 10^−6^ and 5.7 × 10^−4^, respectively). In CESC, adenocarcinoma had mostly high CGI methylation (*P* = 2.7 × 10^−9^). Intestinal and non-diffuse type STADs were associated with lower backbone methylation (*P* = 1.7 × 10^−5^) (Figure 2B). Endometrioid UCEC had both CGI methylation and backbone demethylation (*P* = 1.4 × 10^−12^ and 1.7 × 10^−2^, respectively). Type 2 KIRP, an eosinophilic tumor with worse prognosis, was associated with both CGI methylation and backbone demethylation (*P* = 4.4 × 10^−5^ and 1.9 × 10^−3^, respectively).

Higher histological grade was significantly associated with higher CGI methylation in KIRC, STAD and BLCA (*P* = 1.5 × 10^−6^, 2.5 × 10^−2^ and 2.8 × 10^−2^, respectively) and with lower backbone methylation in LGG (*P* = 6.9 × 10^−6^) (Figure 2). In PRAD, Gleason scores tended to be high both in CGI-methylated and backbone-demethylated tumors (*P* = 6.1 × 10^−3^ and 3.9 × 10^−4^, respectively). An association of CGI methylation with high mitosis was observed in ACC (*P* = 4.5 × 10^−4^).

In many tumors including KICH, KIRP, KIRC, PRAD, BRCA, LUAD, HNSC, ACC and UCEC, increased CGI methylation was associated with advanced stages. In THCA, KIRP, KIRC, BRCA, SKCM, and UCS, decreased backbone methylation was also associated with advanced stages.

### Molecular subtype

As examined by conventional diagnostic tests, some molecular subtypes were associated with methylation. MSI-H tumors were very significantly associated with CGI methylation in COADREAD, STAD and UCEC (*P* = 3.2 × 10^−12^, 2.4 × 10^−7^ and 2.0 × 10^−22^, respectively) (Figure 2B). In COADREAD, CGI methylation was associated with negative expression of MSH6 and PMS2 proteins (*P* = 9.3 × 10^−3^). In BRCA, CGI methylation was significantly associated with positive expression of estrogen and HER2/neu receptors (*P* = 1.5 × 10^−5^ and 8.3 × 10^−8^, respectively) and thus with luminal B subtype (*P* = 1.3 × 10^−9^).

### Gene mutation

The number of gene mutations correlated positively with CGI methylation in THCA, KIRC, LAML, LGG, GBM, PRAD, STAD, BRCA, ACC and UCEC (Figure 2A and B). In line with the previous knowledge, *IDH1* mutation was associated with high CGI methylation in LGG (*P* = 2.1 × 10^−20^), GBM, PRAD and CESC, and *IDH2* was so in LAML. Mutations in *CIC, NOTCH1* and *FUBP1* were very significantly associated with high CGI methylation in LGG (*P* = 5.7 × 10^−11^, 5.2 × 10^−7^ and 9.9 × 10^−5^, respectively), mutation in *PIK3CA* was so in STAD (*P* = 7.4 × 10^−9^), and mutations in *PTEN* and *PIK3R1* were so in UCEC (*P* = 5.9 × 10^−7^ and 1.6 × 10^−4^, respectively). *NRAS* mutation was frequent in THCA with higher CGI methylation and *BRAF* was so in THCA with lower backbone methylation (*P* = 5.9 × 10^−3^ and 9.9 × 10^−7^, respectively) but this may be confounded by the difference in methylation according to histological type, as described above, and high frequency of *NRAS* mutation in follicular and *BRAF* mutation in papillary types (15).

**Figure 3.**
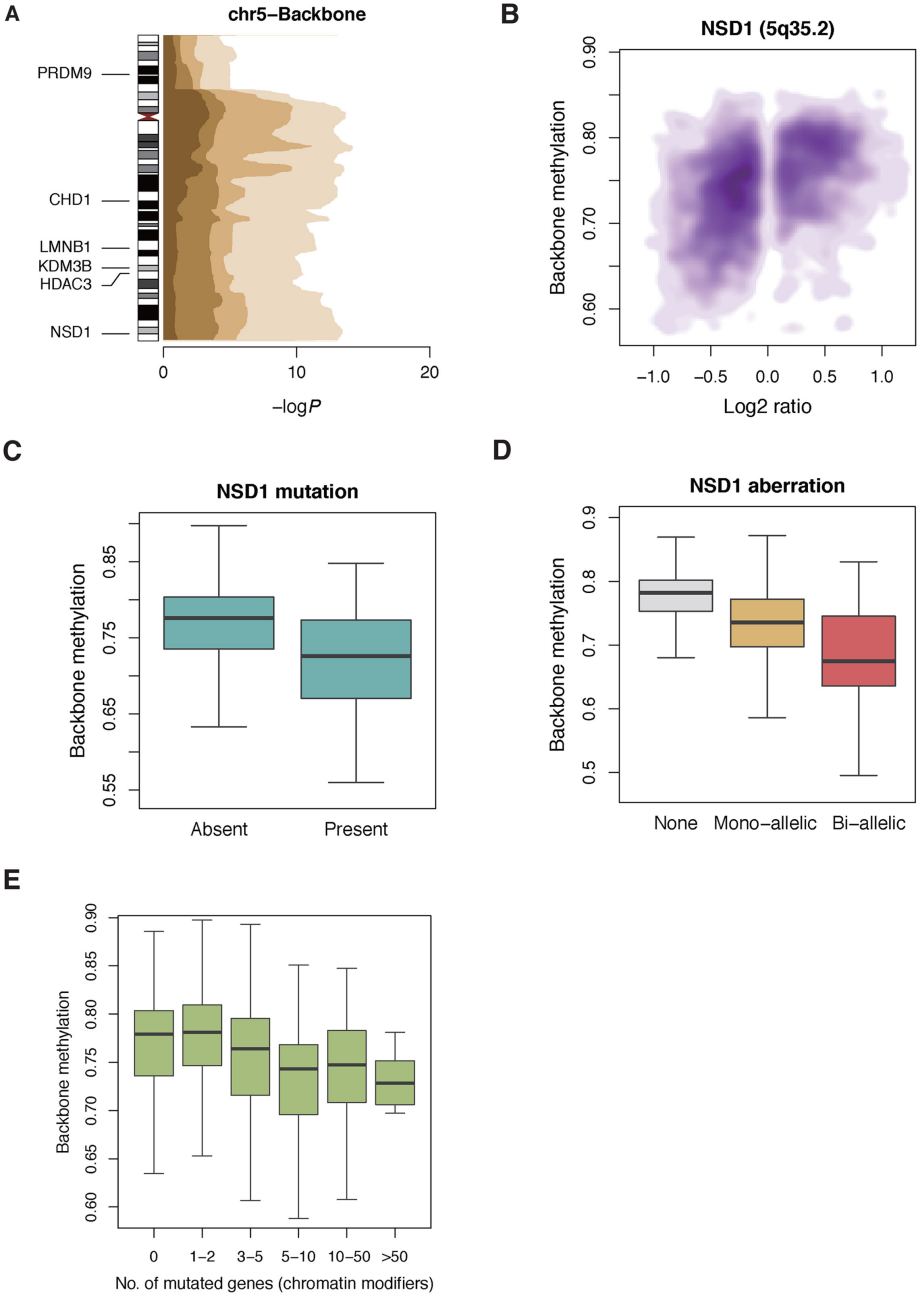
Relation of copy number and somatic mutation with methylation change. (**A**) By concatenating gene-level associations, copy change at 5q is found to be the most recurrently associated region with backbone demethylation. Color gradients are according to the percentiles of 21 tumor types. Chromatin modifier genes in chromosome 5 are presented. (**B**) Copy loss of *NSD1* at 5q35.2 is significantly correlated with backbone demethylation. All types of tumors analyzed together and copy-neutral tumors were removed from the plot. (**C**) *NSD1*-mutated tumors show significant genome-wide backbone demethylation (*P* = 1.9 × 10^−11^). (**D**) Bi-allelic *NSD1* aberration by mutation and/or copy loss shows more profound demethylation (*P* < 2.2 × 10^−16^). (**E**) With increasing number of chromatin modifier mutation, more backbone demethylation is apparent (*P* < 2.2 × 10^−16^).

*TP53* mutation was associated with backbone demethylation in STAD and PRAD (*P* = 5.1 × 10^−5^ and 4.9 × 10^−3^, respectively) (Figure 2C), and *NSD1* mutation was so in HNSC (*P* = 3.2 × 10^−9^) (Supplementary Figure S17B). We also observed that a high number of mutations in chromatin modifier genes is related with more backbone demethylation (Figure 3E).

### Somatic copy number alteration

High number of SCNA significantly correlated with low backbone methylation in LGG, PRAD, COADREAD, STAD, BRCA, LUAD, HNSC, SKCM, LUSC, BLCA and LIHC (Figure 2C and D). In Supplementary Figures S4–25, we presented chromosomal bands and arms showing significant correlations with methylation in each tumor. The most remarkable was the association of chr10 loss and chr7 gain with backbone demethylation (*P* < 1.0 × 10^−35^ and 3.5 × 10^−13^, respectively) and 1p loss and 19q loss with CGI methylation (*P* = 2.1 × 10^−28^ and 7.6 × 10^−26^, respectively) in LGG (Supplementary Figure S9).

To narrow down loci showing significant impact on methylation repeatedly in different tumor types, we performed a Kendall rank correlation test between gene-level copy number (log2ratio) and methylation for each tumor type. Corrected *P* values of individual genes were plotted as ordered by chromosomal positions of the genes, and the 10th, 20th, 30th and 40th percentile *P* values among the 21 tumor types were illustrated in the plot (Supplementary Figures S26 and S27). The most remarkable *P* value peak was in chromosome 5q showing recurrent correlations between deletion and backbone demethylation in many tumors (Figure 3A and B). Candidate genes in this region include *CHD1, LMNB1, KDM3B, HDAC3* and *NSD1*, and among them, *NSD1* was of particular interest in that mutation in this gene also showed the most significant association with backbone demethylation in HNSC and other tumors (Figure 3C). Bi-allelic aberration of *NSD1* either by mutation or copy loss showed more demethylation (Figure 3D). Chromosome 19p was also intriguing in that loss of this region was associated with backbone demethylation and gain was so with CGI methylation (Supplementary Figure S28). Chromatin modifier genes are densely located at 19p, and candidate genes include *SIRT6, KDM4B, LMNB2, MBD3* and many others.

### Gene expression and biological pathways

In contrast to the negative correlation between CGI methylation in promoter and gene expression, backbone methylation in gene body was positively correlated with expression suggesting a role of hypomethylation in gene suppression (Supplementary Figure S29). Such correlations were less prominent in relatively silent tumors such as THCA, KICH, KIRP, KIRC and LAML.

In Supplementary Figures S4–S24, we illustrated the top 100 PARADIGM pathways significantly correlated with methylation along with the name of the pathway superfamily having a large proportion in the top pathways. Among the pathways recurrently associated in several tumors, the bone morphogenic protein (BMP) receptor pathway was significantly suppressed among backbone-demethylated tumors in LGG, PRAD and LIHC and CGI-methylated tumors in LUAD. Significant associations of low lysophosphatidic acid (LPA) receptor pathway activities with backbone demethylation were noted in UCEC, SKCM, BLCA and LIHC, while with CGI methylation in BRCA. The Ephrin B pathway was suppressed in association with backbone demethylation (PRAD, BRCA, SKCM and LIHC) or CGI methylation (HNSC and UCEC). The FoxM1 pathway activity was low in association with backbone demethylation (STAD and LUAD) or CGI methylation (KIRP and KIRC), whereas its activity was high in CGI-methylated KICH tumors. Mitotic kinase pathways including polo-like kinase 1 (PLK1), Aurora A and B pathways are suppressed in association with backbone demethylation (KIRP and PRAD) and CGI methylation (KIRC), while they were active in backbone demethylated LUAD and CGI-methylated KICH tumors. Neurotrophic factor and p75^NTR^ pathways were significantly suppressed only among CGI-methylated tumors in THCA, LAML, STAD, BRCA and HNSC.

### Transcription factors

We also analyzed differential methylation in 12 tumor types with the availability of normal control data. In all tumor types, differentially methylated CpGs were highly enriched in binding sites of polycomb-related SUZ12, EZH2 and CTBP2, and the enrichment rate strongly correlated with the degree of CGI methylation (Supplementary Figure S30). Demethylated CpGs were often enriched in IKZF1, BATF and ZNF217 binding sites but the enrichment rate usually did not correlate with the degree of backbone demethylation, suggesting a minor role of transcription factors in the genome-wide demethylation (Supplementary Figure S31).

### Large demethylation domains

**Figure 4.**
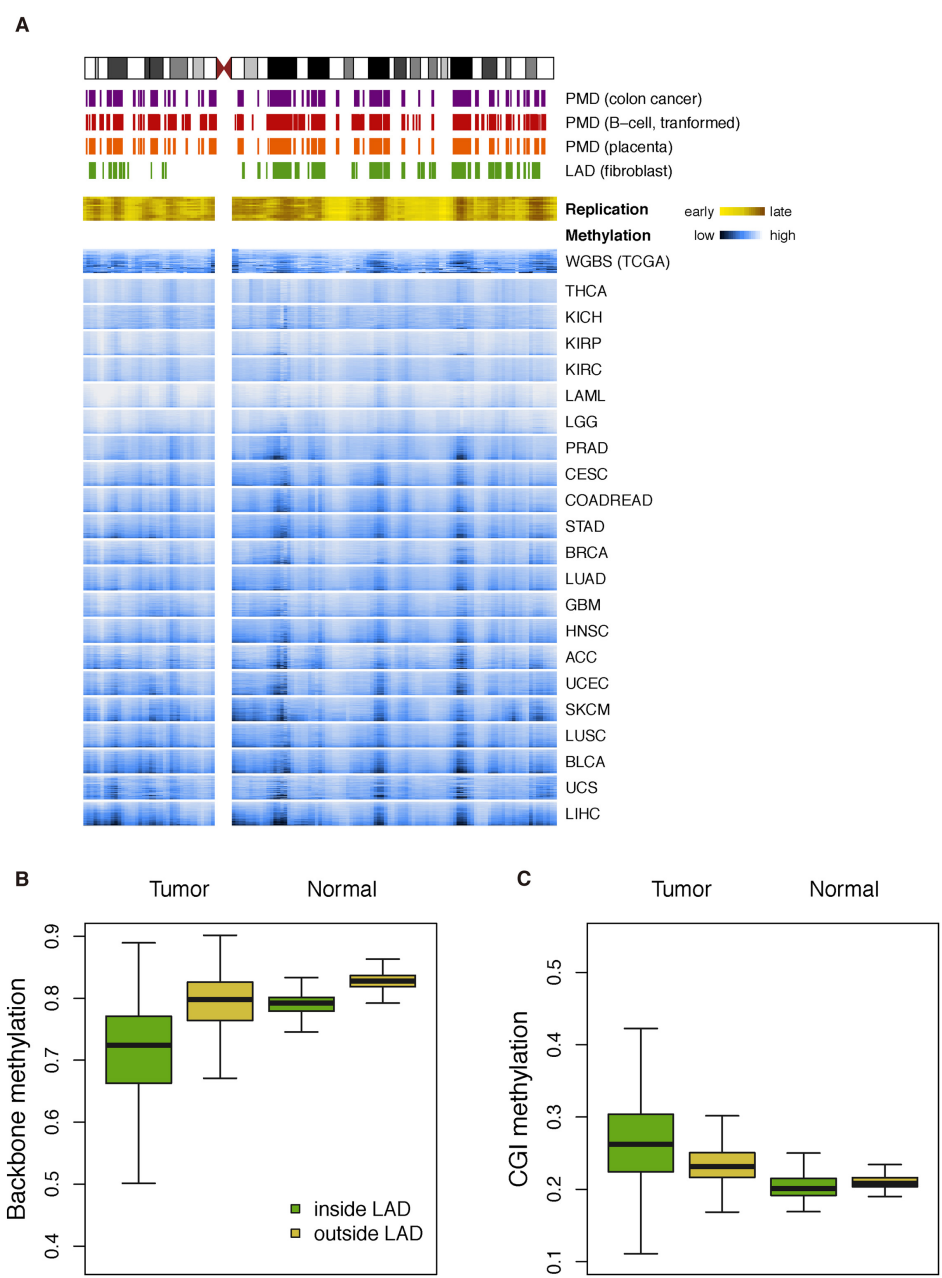
Backbone demethylation in large genomic regions. (**A**) In an exemplary chromosome (chr10) demethylation is profound in large genomic regions that overlap with partially-methylated domains (PMDs), lamina-associated domains (LADs) and late-replicating regions. Values are averaged within each sliding window (2 Mb bin and 1 Mb step). (**B** and **C**) When all tumors are analyzed together, high degrees of backbone demethylation and CGI methylation are observed inside LADs.

In most tumors, profound demethylation was noted in genomic regions that largely overlap with long repressive domains, including PMDs, LADs and late-replicating regions, as previously identified in cancer and normal cultured cells (10,11,16). These regions were generally coincident among different tumor types (Figure 4A). When LADs were used as surrogates for these regions, both backbone demethylation and CGI methylation were more pronounced within LADs (Figure 4B and C; Supplementary Figures S32 and S33).

### Clinical outcome

As to primary treatment success, backbone demethylation was associated with poor response in LGG (*P* = 1.6 × 10^−3^) and CGI methylation was so in ACC (*P* = 6.7 × 10^−3^). Intriguingly, CGI-methylated tumors showed better primary responses in STAD and LUAD (*P* = 7.5 × 10^−3^ and 1.8 × 10^−2^, respectively). In an unadjusted survival analysis, the HC group defined above was associated with poor survival in THCA, KICH, KIRP, KIRC and ACC. The LB group was associated with poor survival in THCA, KICH, KIRP and LGG, whereas intriguingly with favorable outcome in COADREAD (Supplementary Figures S34–S41). However, the associations may be largely confounded by the frequent connection of abnormal methylation with stages and other histopathological features, as mostly insignificant in the Cox proportional hazard models (Supplementary Figures S34–S41). In addition to the well-known fact that LGGs with CGI methylation shows better survival, we found that those with backbone demethylation show worse survival even though they had high CGI methylation (i.e. HC-LB tumors in Figure 1D and Supplementary Figure S38).

## DISCUSSION

Previous studies on cancer epigenetics have mostly focused on CpGs in promoters and CGI where methylation has great impact on gene expression. The definition of CIMP and its associated CpGs varied among different studies without clear consensus (3,4). Our approach was to average all CGIs in nearly 20 thousand genes wherever applicable while finding that direct comparison with the previous CIMP classification was impossible due to the wide heterogeneity of CIMP definition and assay method. For pan-cancer comparison, we rather set a fixed cutoff (0.24 for CGI) inferred from methylation ranges of normal tissues and then defined HC and NC that simply indicate two groups ‘outside’ and ‘within’ the normal range. Even so, the HC-tumors may largely overlap with the CIMP tumors by other previous definitions. As supporting evidence, we could observe correlation of HC-tumors with many CIMP-associated features such as MSI-H and *IDH1* mutation (3,4).

A possible link between CGI methylation and high mutation rate has been suggested with a theory that methylcytosine can be a site for the C-to-T transition (3,4,17). In this study, we found this could be extended and applied to many other tumors. As some studies showed associations of CIMP with stage, often with advanced and occasionally with earlier stages (18,19), we could observe associations between CGI methylation and stage in many previously uninvestigated tumors. The association with histological type and grade may be intriguing since it remained unexplored in many tumors.

Polycomb site methylation has now been accepted as a hallmark of cancers, and so the enrichment of polycomb proteins, SUZ12 and EZH2, should be an innate quality. The co-enrichment of CTBP2 is quite new and interesting in that it is linked to the bivalent mark of ESC (7) and could be a novel candidate for targeted therapy in addition to other polycomb proteins.

Global demethylation in repetitive elements has been suggested in many cancers and yet a systematical analysis is lacking (20,21). We removed repeat regions in the backbone definition due to technical and statistical issues, and nonetheless, the methylation in the backbone may at least in part reflect that in repeat regions since the two regions share many epigenetic properties (20,22). The examination of backbone methylation using array data was supposed to be valid since we observed a good concordance between the array and WGBS calculations and also found a study adopting a similar strategy with ours (12).

Associations of demethylation with histological grade and stage have been suggested in several tumors (23), and we could find such an association in an expanded set of tumors. Associations with histological type in THCA, KIRP, LAML, LGG, UCEC, and STAD have not been described before. The association of backbone demethylation with cigarette smoking in LUAD is intriguing since many previous studies on smoking have focused on promoter CpGs and have shown conflicting results (24–26). We have investigated non-promoter regions, and in line with our results, associations of smoking with demethylation in LINE elements of aero-digestive mucosa (27), esophageal mucosa (28) and blood cells (29) have been reported.

Although it has been experimentally shown that demethylation is linked to mitotic dysfunction and genomic rearrangement and anticipated to cause chromosome instability, it remained uninvestigated in clinical series (21,23,30–32). Here we have presented strong statistical evidences that higher number of SCNA correlates with backbone demethylation in many tumors. The correlation with SCNA was often remarkable in specific chromosomal bands and arms, and we could find some previous literature showing consistent results with ours such as associations with chromosome 9 loss in BLCA (33,34), 8q gain in PRAD (35) and 16p gain in BRCA (36).

In both gene-level copy number and mutation analyses, *NSD1* abnormalities were significantly associated with backbone demethylation. *NSD1* is a SET domain histone methyltransferase that primarily dimethylates histone H3K36, implicated in Sotos and Weaver overgrowth syndromes. Interestingly, a methylome study on patients with Sotos syndrome observed very pronounced demethylation in the *NSD1*-mutated group (37). Although its carcinogenic function is largely unknown, *NSD1* is presumed to have roles in tumorigenesis (6,38). Other candidate genes, *LMNB1* (lamin B1) at 5q23 and *LMNB2* (lamin B2) at 19p13.3, are main components of nuclear lamina and play significant roles in maintaining LAD (39). As an experimental support, lamin B1 is shown to be depleted in senescent cells leading to chromatin reorganization in LAD and possibly linking to aging and cancer development (40). *TP53* was also among the most significant genes, and this can be understood on the basis that *TP53* mutations can directly cause DNA demethylation (41). Moreover, *TP53* plays critical roles DNA repair and maintaining genomic stability (42) and its mutation may exhibit communal pathways with DNA demethylation leading to chromosome instability.

It is often hypothesized that demethylation in cancer cells can activate proto-oncogenes and thus contribute to tumorigenesis (23). This should be true in a specific set of genes, but as a whole, we observed that demethylation of the backbone especially in gene body is more likely to suppress gene expression (Supplementary Figure S29). It may be in line with the perspective that gene body in a highly expressed gene should be maintained hypermethylated to dampen transcriptional noise and help efficient transcription (43–45). As more supporting evidence, we found that many pathways such as BMP receptor, LPA receptor, Ephrin B, PLK1, Aurora A and B, FoxM1, neurotrophic factor and p75^NTR^ pathways tended to be suppressed rather than activated in demethylated tumors.

As the name ‘bone morphogenic protein’ already signifies, BMP pathway has been implicated in the osteoblastic phenotype of bone metastases in PRAD (46,47). We observed that low BMP, especially BMP-7, activity is associated with backbone demethylation and such demethylated tumors show aggressive features like high Gleason score and prostate specific antigen level (Supplementary Figure S10B). This is concordant with many previous studies showing the protective role of BMP-7 and association of its loss with invasive and migratory properties (46,48,49). We also noticed that the demethylated tumors also tended to have worse outcome although the significance varied according to the cutoffs for average backbone methylation (data not shown). BMP signaling pathway also has been implicated in LGG, often with its beneficial effect on tumor inhibition and clinical course (50). We observed low BMP activity in demethylated LGGs which showed higher histological grade and worse treatment response and survival (Supplementary Figure S9B). BMP is also implicated in liver and lung cancers (51,52) and we observed associations of low BMP activities with backbone demethylation and CGI methylation in LIHC and LUAD, respectively.

LPA is a bioactive phospholipid which through G protein-coupled receptors induce various cellular responses including cell proliferation, differentiation, morphogenesis, cell migration, cytokine production and many others (53). Accordingly, LPA pathway is implicated in a number of cancers, often as pro-tumorigenic and sometimes as anti-tumorigenic (54). In our analysis, low LPA receptor pathway was often associated with backbone demethylation. Interestingly, these tumors showed more profound demethylation than other tumors (Figure 1C) with relatively weak or negligible associations of the demethylation with tumor aggressiveness. Eph receptors and their Eph receptor-interacting (Ephrin) ligands have been implicated in a number of tumors with dichotomic effects that both increased and decreased activities are linked to tumor progression (55). We observed that Ephrin pathways are epigenetically suppressed in a set of tumors preferentially by backbone demethylation.

Mitotic kinases including PLK1 and Aurora kinase have unique roles in mitosis and their overexpression has been suggested to be associated with invasiveness and chromosome instability in many tumors (56). We found that PLK1 and Aurora pathways are often suppressed and sometimes activated in backbone-demethylated or CGI-methylated tumors. It is notable that the pathways also act as mitotic bodyguards for confident cell division (57) and a low activity can also lead to chromosome instability (56). The FoxM transcription factors, targeting cell cycle regulators like cyclins, PLK1 and Aurora kinases, are also crucial for cell cycle phase progression and mitosis (58). Either loss or gain of FoxM function can alter cell fate and promote tumorigenesis (59), and we observed epigenetic suppression of FoxM1 pathway in a set of tumors. Inversely, FoxM1 has been shown to directly reshape the epigenomic landscape of tumor cells (60). Neurotrophin and its receptor p75^NTR^ have critical roles in ESC pluripotency and their dysregulation has been implicated in many tumors (61). They were specifically dysregulated in CGI-methylated tumors in our analysis, and this may be understood in parallel with the high enrichment of methylated CpGs in ESC-related TFs (Supplementary Figure S30).

Through advanced genomic technologies, recent literature reports have noted that both normal and cancer cells have long repressive domains largely overlapping with LADs and having unique epigenetic properties (16,62). These domains have been recognized as PMDs in EBV-transformed cell-lines (11), normal placenta (12) and cultured cancer cell-lines (10,63). Timp *et al*. suggested that these regions are largely concordant across breast, colon, lung, pancreas and thyroid cancers (8), and here we confirm that it is true in almost all tumors but the degrees of demethylation in these regions vary among different tumors (Figure 3). Consistent with previous studies (10,12), we observed a paradoxically high CGI methylation in those demethylated domains, which may in part reflect the high overlap between backbone demethylation and CGI methylation in the vast majority of tumors (Figure 1). Such high overlaps may also explain the concomitant associations of some parameters with both backbone demethylation and CGI methylation.

We also observed that intra-tumoral heterogeneity may exist with some clinical implications. For example, most THCA tumors belonged to NC-NB tumors by our pan-cancer cutoffs, and however, two clusters with slightly-high and slightly-low CGI methylation existed even within the normal range. The slightly-high tumors showed poor survival compared to slightly-low tumors. Likewise, a cluster of LGGs with very high CGI methylation in the HC-NB group existed and showed worse survival. The data are not shown because the current study is focused on pan-cancer comparison and confounding variables are not perfectly controlled. Furthermore, some tumors like KICH, GBM, ACC and UCS had limited number of cases. Therefore such heterogeneity may be pursued in subsequent studies with tumor-specific considerations and with more thorough investigation and control of covariates.

**Figure 5.**
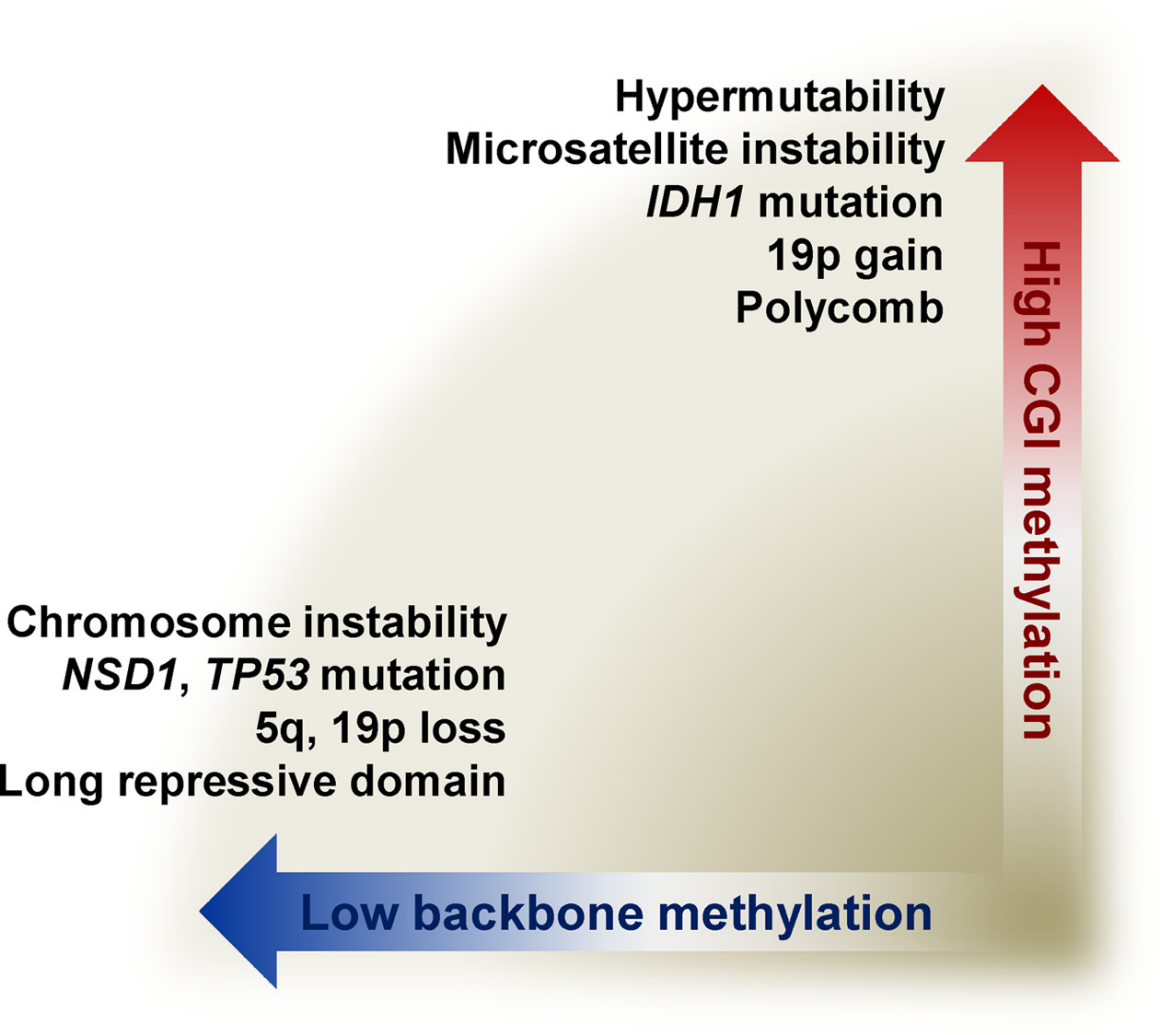
Comprehensive model for CGI methylation and backbone demethylation.

Collectively, we present a pan-cancer model connecting CGI methylation with hypermutability, MSI-H, *IDH1* mutation, 19p gain and polycomb proteins and backbone demethylation with chromosomal instability, *NSD1* and *TP53* mutations, 5q and 19p loss and long repressive domains (Figure 5). For therapeutic implications, one could surmise that demethylating agents could be applied by considering degrees of CGI methylation and backbone demethylation in each tumor. Since many pathways are suppressed in methylated and demethylated tumors, thoughtful usage of new targeted drugs inhibiting such pathways is warranted.

## Supplementary Material

SUPPLEMENTARY DATA

## References

[1] Lee S.T., Xiao Y., Muench M.O., Xiao J., Fomin M.E., Wiencke J.K., Zheng S., Dou X., de Smith A., Chokkalingam A. (2012). A global DNA methylation and gene expression analysis of early human B-cell development reveals a demethylation signature and transcription factor network. Nucleic Acids Res..

[2] Baylin S.B., Jones P.A. (2011). A decade of exploring the cancer epigenome - biological and translational implications. Nat. Rev. Cancer.

[3] Rideout W.M. 3rd, Coetzee G.A., Olumi A.F., Jones P.A. (1990). 5-Methylcytosine as an endogenous mutagen in the human LDL receptor and p53 genes. Science.

[4] Schmutte C., Yang A.S., Nguyen T.T., Beart R.W., Jones P.A. (1996). Mechanisms for the involvement of DNA methylation in colon carcinogenesis. Cancer Res..

[5] Jones P.A., Baylin S.B. (2007). The epigenomics of cancer. Cell.

[6] Shen H., Laird P.W. (2013). Interplay between the cancer genome and epigenome. Cell.

[7] Lee S.T., Muench M.O., Fomin M.E., Xiao J., Zhou M., de Smith A., Martin-Subero J.I., Heath S., Houseman E.A., Roy R. (2015). Epigenetic remodeling in B-cell acute lymphoblastic leukemia occurs in two tracks and employs embryonic stem cell-like signatures. Nucleic Acids Res..

[8] Timp W., Bravo H.C., McDonald O.G., Goggins M., Umbricht C., Zeiger M., Feinberg A.P., Irizarry R.A. (2014). Large hypomethylated blocks as a universal defining epigenetic alteration in human solid tumors. Genome Med..

[9] Carter S.L., Cibulskis K., Helman E., McKenna A., Shen H., Zack T., Laird P.W., Onofrio R.C., Winckler W., Weir B.A. (2012). Absolute quantification of somatic DNA alterations in human cancer. Nat. Biotechnol..

[10] Berman B.P., Weisenberger D.J., Aman J.F., Hinoue T., Ramjan Z., Liu Y., Noushmehr H., Lange C.P., van Dijk C.M., Tollenaar R.A. (2012). Regions of focal DNA hypermethylation and long-range hypomethylation in colorectal cancer coincide with nuclear lamina-associated domains. Nat. Genet..

[11] Hansen K.D., Sabunciyan S., Langmead B., Nagy N., Curley R., Klein G., Klein E., Salamon D., Feinberg A.P. (2014). Large-scale hypomethylated blocks associated with Epstein-Barr virus-induced B-cell immortalization. Genome Res..

[12] Schroeder D.I., Blair J.D., Lott P., Yu H.O., Hong D., Crary F., Ashwood P., Walker C., Korf I., Robinson W.P. (2013). The human placenta methylome. Proc. Natl. Acad. Sci. U.S.A..

[13] Gonzalez-Perez A., Jene-Sanz A., Lopez-Bigas N. (2013). The mutational landscape of chromatin regulatory factors across 4,623 tumor samples. Genome Biol..

[14] Cancer Genome Atlas Research Network (2014). Comprehensive molecular characterization of gastric adenocarcinoma. Nature.

[15] Hsiao S.J., Nikiforov Y.E. (2014). Molecular approaches to thyroid cancer diagnosis. Endocr. Relat. Cancer.

[16] Guelen L., Pagie L., Brasset E., Meuleman W., Faza M.B., Talhout W., Eussen B.H., de Klein A., Wessels L., de Laat W. (2008). Domain organization of human chromosomes revealed by mapping of nuclear lamina interactions. Nature.

[17] Cancer Genome Atlas Research Network (2012). Comprehensive molecular characterization of human colon and rectal cancer. Nature.

[18] Hughes L.A., Melotte V., de Schrijver J., de Maat M., Smit V.T., Bovee J.V., French P.J., van den Brandt P.A., Schouten L.J., de Meyer T. (2013). The CpG island methylator phenotype: what's in a name?. Cancer Res..

[19] Suzuki H., Yamamoto E., Maruyama R., Niinuma T., Kai M. (2014). Biological significance of the CpG island methylator phenotype. Biochem. Biophys. Res. Commun..

[20] Ehrlich M. (2009). DNA hypomethylation in cancer cells. Epigenomics.

[21] Cheishvili D., Boureau L., Szyf M. (2015). DNA demethylation and invasive cancer: implications for therapeutics. Br. J. Pharmacol..

[22] Su J., Shao X., Liu H., Liu S., Wu Q., Zhang Y. (2012). Genome-wide dynamic changes of DNA methylation of repetitive elements in human embryonic stem cells and fetal fibroblasts. Genomics.

[23] Wilson A.S., Power B.E., Molloy P.L. (2007). DNA hypomethylation and human diseases. Biochim. Biophys. Acta.

[24] Huang T., Chen X., Hong Q., Deng Z., Ma H., Xin Y., Fang Y., Ye H., Wang R., Zhang C. (2015). Meta-analyses of gene methylation and smoking behavior in non-small cell lung cancer patients. Sci. Rep..

[25] Lyn-Cook L., Word B., George N., Lyn-Cook B., Hammons G. (2014). Effect of cigarette smoke condensate on gene promoter methylation in human lung cells. Tobacco Induced Dis..

[26] Liu H., Zhou Y., Boggs S.E., Belinsky S.A., Liu J. (2007). Cigarette smoke induces demethylation of prometastatic oncogene synuclein-gamma in lung cancer cells by downregulation of DNMT3B. Oncogene.

[27] Hsiung D.T., Marsit C.J., Houseman E.A., Eddy K., Furniss C.S., McClean M.D., Kelsey K.T. (2007). Global DNA methylation level in whole blood as a biomarker in head and neck squamous cell carcinoma. Cancer Epidemiol. Biomarkers Prev..

[28] Shigaki H., Baba Y., Watanabe M., Iwagami S., Miyake K., Ishimoto T., Iwatsuki M., Baba H. (2012). LINE-1 hypomethylation in noncancerous esophageal mucosae is associated with smoking history. Ann. Surg. Oncol..

[29] Choi J.Y., James S.R., Link P.A., McCann S.E., Hong C.C., Davis W., Nesline M.K., Ambrosone C.B., Karpf A.R. (2009). Association between global DNA hypomethylation in leukocytes and risk of breast cancer. Carcinogenesis.

[30] Thomas J.H. (1995). Genomic imprinting proposed as a surveillance mechanism for chromosome loss. Proc. Natl. Acad. Sci. U.S.A..

[31] Eden A., Gaudet F., Waghmare A., Jaenisch R. (2003). Chromosomal instability and tumors promoted by DNA hypomethylation. Science.

[32] Karpf A.R., Matsui S. (2005). Genetic disruption of cytosine DNA methyltransferase enzymes induces chromosomal instability in human cancer cells. Cancer Res..

[33] Moore L.E., Pfeiffer R.M., Poscablo C., Real F.X., Kogevinas M., Silverman D., Garcia-Closas R., Chanock S., Tardon A., Serra C. (2008). Genomic DNA hypomethylation as a biomarker for bladder cancer susceptibility in the Spanish Bladder Cancer Study: a case-control study. Lancet Oncol..

[34] Nakagawa T., Kanai Y., Ushijima S., Kitamura T., Kakizoe T., Hirohashi S. (2005). DNA hypomethylation on pericentromeric satellite regions significantly correlates with loss of heterozygosity on chromosome 9 in urothelial carcinomas. J. Urol..

[35] Schulz W.A., Elo J.P., Florl A.R., Pennanen S., Santourlidis S., Engers R., Buchardt M., Seifert H.H., Visakorpi T. (2002). Genomewide DNA hypomethylation is associated with alterations on chromosome 8 in prostate carcinoma. Genes Chromosomes Cancer.

[36] Tsuda H., Takarabe T., Kanai Y., Fukutomi T., Hirohashi S. (2002). Correlation of DNA hypomethylation at pericentromeric heterochromatin regions of chromosomes 16 and 1 with histological features and chromosomal abnormalities of human breast carcinomas. Am. J. Pathol..

[37] Chonufani S., Cytrynbaum C., Chung B., Turinsky A., Grafodatskaya D., Chen Y., Luk H., Lo I., Lam S., Stavropoulos D. (2014). The 64th Annual Meeting of the American Society of Human Genetics (ASHG 2014).

[38] Morishita M., Luccio E. (2011). Cancers and the NSD family of histone lysine methyltransferases. Biochim. Biophys. Acta.

[39] Butin-Israeli V., Adam S.A., Goldman A.E., Goldman R.D. (2012). Nuclear lamin functions and disease. Trends Genet..

[40] Shah P.P., Donahue G., Otte G.L., Capell B.C., Nelson D.M., Cao K., Aggarwala V., Cruickshanks H.A., Rai T.S., McBryan T. (2013). Lamin B1 depletion in senescent cells triggers large-scale changes in gene expression and the chromatin landscape. Genes Dev..

[41] Nasr A.F., Nutini M., Palombo B., Guerra E., Alberti S. (2003). Mutations of TP53 induce loss of DNA methylation and amplification of the TROP1 gene. Oncogene.

[42] Hanel W., Moll U.M. (2012). Links between mutant p53 and genomic instability. J. Cell. Biochem..

[43] Suzuki M.M., Bird A. (2008). DNA methylation landscapes: provocative insights from epigenomics. Nat. Rev. Genetics.

[44] Ball M.P., Li J.B., Gao Y., Lee J.H., LeProust E.M., Park I.H., Xie B., Daley G.Q., Church G.M. (2009). Targeted and genome-scale strategies reveal gene-body methylation signatures in human cells. Nat. Biotechnol..

[45] Hon G.C., Hawkins R.D., Caballero O.L., Lo C., Lister R., Pelizzola M., Valsesia A., Ye Z., Kuan S., Edsall L.E. (2012). Global DNA hypomethylation coupled to repressive chromatin domain formation and gene silencing in breast cancer. Genome Res..

[46] Buijs J.T., Petersen M., van der Horst G., van der Pluijm G. (2010). Bone morphogenetic proteins and its receptors; therapeutic targets in cancer progression and bone metastasis?. Curr. Pharm. Des..

[47] Kim I.Y., Lee D.H., Lee D.K., Ahn H.J., Kim M.M., Kim S.J., Morton R.A. (2004). Loss of expression of bone morphogenetic protein receptor type II in human prostate cancer cells. Oncogene.

[48] Ye L., Lewis-Russell J.M., Kynaston H., Jiang W.G. (2007). Endogenous bone morphogenetic protein-7 controls the motility of prostate cancer cells through regulation of bone morphogenetic protein antagonists. J. Urol..

[49] Buijs J.T., Rentsch C.A., van der Horst G., van Overveld P.G., Wetterwald A., Schwaninger R., Henriquez N.V., Ten Dijke P., Borovecki F., Markwalder R. (2007). BMP7, a putative regulator of epithelial homeostasis in the human prostate, is a potent inhibitor of prostate cancer bone metastasis in vivo. Am. J. Pathol..

[50] Gonzalez-Gomez P., Anselmo N.P., Mira H. (2014). BMPs as therapeutic targets and biomarkers in astrocytic glioma. Biomed. Res. Int..

[51] Herrera B., Dooley S., Breitkopf-Heinlein K. (2014). Potential roles of bone morphogenetic protein (BMP)-9 in human liver diseases. Int. J. Mol. Sci..

[52] Langenfeld E., Hong C.C., Lanke G., Langenfeld J. (2013). Bone morphogenetic protein type I receptor antagonists decrease growth and induce cell death of lung cancer cell lines. PloS One.

[53] Yung Y.C., Stoddard N.C., Chun J. (2014). LPA receptor signaling: pharmacology, physiology, and pathophysiology. J. Lipid Res..

[54] Lin M.E., Herr D.R., Chun J. (2010). Lysophosphatidic acid (LPA) receptors: signaling properties and disease relevance. Prostaglandins Other Lipid Mediat..

[55] Pasquale E.B. (2010). Eph receptors and ephrins in cancer: bidirectional signalling and beyond. Nat. Rev. Cancer.

[56] Li J.J., Li S.A. (2006). Mitotic kinases: the key to duplication, segregation, and cytokinesis errors, chromosomal instability, and oncogenesis. Pharmacol. Ther..

[57] Salaun P., Rannou Y., Prigent C. (2008). Cdk1, Plks, Auroras, and Neks: the mitotic bodyguards. Adv. Exp. Med. Biol..

[58] Raychaudhuri P., Park H.J. (2011). FoxM1: a master regulator of tumor metastasis. Cancer Res..

[59] Myatt S.S., Lam E.W. (2007). The emerging roles of forkhead box (Fox) proteins in cancer. Nat. Rev. Cancer.

[60] Teh M.T., Gemenetzidis E., Patel D., Tariq R., Nadir A., Bahta A.W., Waseem A., Hutchison I.L. (2012). FOXM1 induces a global methylation signature that mimics the cancer epigenome in head and neck squamous cell carcinoma. PloS One.

[61] Tomellini E., Lagadec C., Polakowska R., Le Bourhis X. (2014). Role of p75 neurotrophin receptor in stem cell biology: more than just a marker. Cell. Mol. Life Sci..

[62] Luperchio T.R., Wong X., Reddy K.L. (2014). Genome regulation at the peripheral zone: lamina associated domains in development and disease. Curr. Opin. Genet. Dev..

[63] Ziller M.J., Gu H., Muller F., Donaghey J., Tsai L.T., Kohlbacher O., De Jager P.L., Rosen E.D., Bennett D.A., Bernstein B.E. (2013). Charting a dynamic DNA methylation landscape of the human genome. Nature.

