# Supplementary material for: Genome-wide CpG island methylation and intergenic demethylation propensities vary among different tumor sites
Source: Nucleic Acids Res. 2015 Oct 12;44(3):1105–17. doi: 10.1093/nar/gkv1038 (PMC4756811; doi:10.1093/nar/gkv1038)
Supplement: SUPPLEMENTARY DATA [file supp_44_3_1105__index.html]

Genome-wide CpG island methylation and intergenic demethylation propensities vary among different tumor sites — SUPPLEMENTARY DATA 

# Genome-wide CpG island methylation and intergenic demethylation propensities vary among different tumor sites

## SUPPLEMENTARY DATA

- SUPPLEMENTARY DATA
- SUPPLEMENTARY DATA
