# Supplementary material for: Genome-wide CpG island methylation and intergenic demethylation propensities vary among different tumor sites
Source: Nucleic Acids Res. 2015 Oct 12;44(3):1105–17. doi: 10.1093/nar/gkv1038 (PMC4756811; doi:10.1093/nar/gkv1038)
Supplement: SUPPLEMENTARY DATA [file supp_gkv1038_nar-02676-x-2015-File007.pdf]

Supplementary Data

Table S1 Patient demographics and data analyzed

Group	Tumor (n)	Normal (n)	Female/Male (n/n)	Age (Mean ± SD)	Mutation analyzed (n)	SCNA analyzed (n)	Gene expression analyzed (n)	miRNA analyzed (n)
**ACC**	80	0	46/28	46.6 ± 16.0	79	78	79	80
**BLCA**	253	21	51/159	67.7 ± 10.7	130	247	241	252
**BRCA**	731	96	661/7	57.9 ± 13.1	648	709	709	711
**CESC**	210	3	168/0	47.6 ± 13.3	39	192	185	200
**COADREAD**	378	45	167/201	64.8 ± 12.9	8	355	366	337
**GBM**	123	1	49/65	60.9 ± 12.2	108	120	47	0
**HNSC**	516	50	120/297	61.0 ± 12.2	306	511	497	511
**KICH**	66	0	27/39	51.5 ± 14.3	66	66	66	66
**KIRC**	318	160	98/192	61.5 ± 11.9	243	297	303	304
**KIRP**	211	45	51/107	60.2 ± 12.9	153	181	182	182
**LAML**	194	0	89/105	55.1 ± 16.0	191	185	170	185
**LGG**	511	0	170/197	43.3 ± 13.5	285	458	458	434
**LIHC**	237	50	61/95	61.5 ± 14.1	59	198	190	199
**LUAD**	435	32	217/186	65.4 ± 10.0	110	421	426	433
**LUSC**	359	42	70/208	67.6 ± 8.9	74	356	358	354
**PRAD**	336	49	0/240	60.4 ± 7.1	261	331	333	326
**SKCM**	385	2	135/211	56.9 ± 15.6	31	297	289	282
**STAD**	325	2	104/170	65.4 ± 10.6	176	303	231	275
**THCA**	496	56	358/130	47.2 ± 15.6	402	494	494	495
**UCEC**	416	46	372/0	63.9 ± 11.4	135	406	414	405
**UCS**	57	0	57/0	69.7 ± 9.3	18	56	57	56
**Total**	**6,637**	**700**	**3,072/2,637**	**58.7 ± 14.6**	**3,522**	**6,261**	**6,095**	**6,087**

ACC, adrenocortical carcinoma ; BLCA, bladder urothelial carcinoma ; BRCA, breast invasive carcinoma ; CESC, cervical squamous cell carcinoma and endocervical adenocarcinoma ; COADREAD, colorectal adenocarcinoma; GBM, glioblastoma multiforme ; HNSC, head and neck squamous cell carcinoma ; KICH, kidney chromophobe ; KIRC, kidney renal clear cell carcinoma ; KIRP, kidney renal papillary cell carcinoma ; LAML, acute myeloid leukemia ; LGG, brain lower grade glioma ; LIHC, liver hepatocellular carcinoma ; LUAD, lung adenocarcinoma ; LUSC, lung squamous cell carcinoma ; PRAD, prostate adenocarcinoma ; SKCM, skin cutaneous melanoma ; STAD, stomach adenocarcinoma ; THCA, thyroid carcinoma ; UCEC, uterine corpus endometrial carcinoma ; UCS, uterine carcinosarcoma ;

Table S6 Methylation levels according to gender

Tumor type	Backbone methylation			CGI methylation		
	Female	Male	*P*	Female	Male	*P*
**ACC**	0.75 ± 0.06	0.75 ± 0.05	$5.7×10^{-1}$	0.23 ± 0.04	0.24 ± 0.03	$6.3×10^{-1}$
**BLCA**	0.71 ± 0.06	0.70 ± 0.06	$2.0×10^{-1}$	0.23 ± 0.02	0.23 ± 0.03	$8.7×10^{-1}$
**COADREAD**	0.74 ± 0.04	0.75 ± 0.05	$1.6×10^{-1}$	0.26 ± 0.03	0.27 ± 0.03	$6.6×10^{-1}$
**GBM**	0.76 ± 0.05	0.77 ± 0.05	$1.7×10^{-1}$	0.23 ± 0.02	0.24 ± 0.02	$6.5×10^{-2}$
**HNSC**	0.74 ± 0.05	0.74 ± 0.05	$3.7×10^{-1}$	0.24 ± 0.02	0.24 ± 0.02	$5.0×10^{-1}$
**KICH**	0.78 ± 0.02	0.77 ± 0.04	$1.2×10^{-2}$	0.20 ± 0.01	0.21 ± 0.02	$3.4×10^{-1}$
**KIRC**	0.79 ± 0.02	0.79 ± 0.03	$9.5×10^{-1}$	0.22 ± 0.01	0.22 ± 0.02	$9.2×10^{-3}$
**KIRP**	0.81 ± 0.04	0.83 ± 0.02	$3.0×10^{-2}$	0.24 ± 0.04	0.22 ± 0.02	$1.4×10^{-2}$
**LAML**	0.84 ± 0.02	0.84 ± 0.03	$5.0×10^{-1}$	0.24 ± 0.02	0.25 ± 0.04	$1.3×10^{-1}$
**LGG**	0.81 ± 0.03	0.82 ± 0.03	$5.6×10^{-2}$	0.27 ± 0.03	0.27 ± 0.03	$2.2×10^{-1}$
**LIHC**	0.71 ± 0.09	0.68 ± 0.08	$7.7×10^{-2}$	0.24 ± 0.04	0.23 ± 0.03	$2.9×10^{-1}$
**LUAD**	0.75 ± 0.03	0.73 ± 0.04	$8.9×10^{-7}$	0.25 ± 0.02	0.25 ± 0.02	$5.4×10^{-2}$
**LUSC**	0.74 ± 0.05	0.73 ± 0.06	$1.5×10^{-1}$	0.22 ± 0.02	0.22 ± 0.02	$4.8×10^{-1}$
**SKCM**	0.72 ± 0.06	0.72 ± 0.06	$7.4×10^{-1}$	0.23 ± 0.03	0.23 ± 0.03	$9.8×10^{-1}$
**STAD**	0.73 ± 0.05	0.73 ± 0.05	$1.9×10^{-1}$	0.26 ± 0.04	0.27 ± 0.05	$9.7×10^{-2}$
**THCA**	0.80 ± 0.02	0.80 ± 0.02	$4.4×10^{-1}$	0.21 ± 0.01	0.21 ± 0.01	$5.6×10^{-1}$

Supplementary Figure Legends

Figure S1

Comparison of tumor purity with methylation in 4,724 tumors (those could be estimated by the ABSOLUTE algorithm, Broad Institute). Purity does not correlate significantly with backbone or CGI methylation, suggesting a minor effect of any cell purity bias.

Figure S2

Backbone and CGI methylation in each tumor and normal control; x-axis displays average backbone methylation level and y-axis displays the average CGI methylation level. Normal adjacent tissues consistently show low CGI methylation (~0.2) and high backbone methylation (~0.8), whereas tumor tissues have variable *de novo* methylation of CGIs and demethylation of backbones. The degree and distribution of CGI or backbone changes differ according to tumor type. THCA, KICH, KIRP and KIRC have methylation levels mostly similar to those of normal adjacent tissues. LAML and LGG have mostly higher methylation levels than the normal tissue range. The remaining tumors are heterogeneous with variable degrees of CGI and backbone methylation in each tumor. Medians of BLCA, UCS and LIHC are more skewed to backbone demethylation.

Figure S3

Correlation of methylation with age. (**A**) In general, CGI are slightly more methylated with increasing age (r=0.040; P=2.4×10^{-3}). The large smooth scatter plots in left upper side indicate the association among all tumors combined. (**B**) The backbone is gradually demethylated with increasing age (r=−0.241; P=1.5×10^{-75}) and the correlations are different according to tumor type.

Figure S4

Correlation of methylation with clinical, pathological and molecular variables in thyroid cancer (THCA). (**A**) High CGI methylation is associated with follicular histological subtype and previous history of lymphocytic thyroiditis. Methylation, expression and miRNA clusters (best clusters defined by the Firehose GDAC pipeline) are very significantly correlated with the degree of CGI methylation. CGI-methylated tumors have increased number of mutations. From the PARADIGM pathway enrichment scores using the SCNA and RNA expression data (also generated by the Firehose pipeline), it is observed that low activities of ErbB4, $p75^{NTR}$, Fc-epsilon receptor and interleukin-1 (IL-1) pathways are related with CGI methylation. (**B**) Backbone demethylation is associated with increased N and T

stages, and papillary and non-follicular histology. Methylation, expression and miRNA clusters are correlated with the degree of demethylation with high significance. *NRAS* mutant tumors are less demethylated while *BRAF* mutants are more demethylated. It is noted that low activities of c-Kit, Hedgehog, syndecan-2 and c-Met pathways and high activity of FAS pathway were more frequently noted in demethylated tumors

Figure S5

Correlation of methylation with clinical, pathological and molecular variables in kidney chromophobe carcinoma (KICH). (**A**) CGI methylation is correlated with advanced stages. Molecular clusters were marginally significant. CGI-methylated tumors show higher activities of FoxM1 and PLK1 pathways. (**B**) Backbone demethylation is associated with sarcomatoid features and high white blood cell (WBC) counts. Association with molecular clusters is not evident in KICH, probably in part due to the small number of cases. Demethylated tumors have a high mutation rate. No pathway was significant.

Figure S6

Correlation of methylation with clinical, pathological and molecular variables in kidney renal papillary cell carcinoma (KIRP). (**A**) Higher CGI methylation is related with advanced stage and type 2 tumors. Methylation, RNA expression and SCNA clusters are also significantly related. CGI-methylated tumors have higher number of SCNAs, especially loss of chromosomes 22q, 13q, 18 and 1p36. Gain of chromosomes 7 and 17p are more frequently observed in non-methylated tumors. CGI methylators show low activities of c-Kit, and FoxM1 pathways. (**B**) Backbone demethylation is associated with advanced stages, type 2 tumor, high platelet count and low Karnofsky performance score (poor general condition). Associations with methylation and RNA expression clusters are significant but those with miRNA and SCNA clusters are borderline or negative. Demethylated tumors have small number of somatic mutations and low activities of Aurora B and PLK1 pathways.

Figure S7

Correlation of methylation with clinical, pathological and molecular variables in kidney renal clear cell carcinoma (KIRC). (**A**) CGI methylation is related with advanced stage, high grade and left laterality. All molecular clusters are significantly associated with methylation status. CGI-methylated tumors frequently have loss of chromosomes 4, 9 and 14q and gain of chromosomes 20 and 7q36. Those also have higher number of somatic mutations, especially in *SETD2*, *BAP1* and *PBRM1*, and low activities of PLK1, Aurora B,

FoxM1 and interleukin-4 pathways. (**B**) Backbone demethylation is associated with advanced T stage and the low activity of erythropoietin pathway. Associations with molecular clusters are negative or borderline.

Figure S8

Correlation of methylation with clinical, pathological and molecular variables in acute myeloid leukemia (LAML). (**A**) High CGI methylation is associated with FAB M0, M1 and M2 subtypes. Methylation, RNA expression and miRNA clusters are also correlated. *DNMT3A*-mutated tumors have less CGI methylation while *IDH2*-mutated tumors have higher CGI methylation. Low activities of SMAD2/3 and $p75^{NTR}$ pathways are also notable in highly methylated tumors. (**B**) Backbone demethylation is associated with FAB M5 subtype, methylation and miRNA clusters, and low activity of ErbB4 pathway.

Figure S9

Correlation of methylation with clinical, pathological and molecular variables in brain lower grade glioma (LGG). (**A**) High CGI methylation is associated with low histological grade, oligodendroglioma histology, frontal lobe location and slightly low Karnofsky score. All molecular clusters are associated significantly. CGI-methylated tumors have small number of SCNAs but frequently have loss of chromosomes 4, 19q and 1p. Mutation rates are high in those tumors, especially insertion/deletion mutations, with frequent *IDH1*, *CIC*, *NOTCH1* and *FUBP1* mutations and infrequent *EGFR* and *PTEN* mutations. Low activity of syndecan-3 pathway and high activities of Ret and BMP receptor pathways are noted with increased methylation. (**B**) Backbone demethylation is associated high histological grade, astrocytoma histology, temporal lobe location, low Karnofsky score and poor treatment response. All molecular clusters are associated very significantly. Demethylated tumors are also related with high number of SCNAs with loss of chromosomes 10, 9p21 and 22q13 and gain of chromosomes 7, 20 and 4q12. Co-deletion of 1p/19q deletion, a common abnormality in LGG, is less frequent in demethylated tumors. Those also have slightly high number of somatic mutations, especially in *EGFR*, *PTEN* and *STK19*, but harbor less mutations in *IDH1*, *CIC* and *FUBP1*. Low activities of EGFR, BMP receptor and Ret pathways are noted with demethylation.

Figure S10

Correlation of methylation with clinical, pathological and molecular variables in prostate adenocarcinoma (PRAD). (**A**) High CGI methylation is correlated with high Gleason score, high PSA level and advanced T stage. All molecular clusters are significantly associated. CGI-methylated tumors have high number of SCNAs, with frequent loss of 13q14, 8p21 and 16q22, and high number of mutations. Low activities of endothelin, angiotensin receptor, c-Kit, c-Met, ErbB2/ErbB3 and S1P1 pathways are noted with increasing methylation. (**B**) Backbone demethylation is associated with high Gleason score, high preoperative prostate-specific antigen (PSA) level and extra-capsular extension. All molecular clusters are significantly associated. Demethylated tumors have high number of SCNAs with frequent gain of chromosomes 7, 8q, 3q and 8q24 and loss of 17p, 6q14 and 5q21. They also have frequent *TP53* mutation and low activities of Ephrin B, Aurora A, Hedgehog and BMP receptor pathways.

Figure S11

Correlation of methylation with clinical, pathological and molecular variables in cervical squamous cell carcinoma and endocervical adenocarcinoma (CESC). (**A**) High CGI methylation is correlated with low grade and adenocarcinoma histology. Methylation, RNA expression and miRNA clusters are significantly associated. Dysregulations of glucocorticoid receptor, caspase apoptosis and nectin adhesion pathways are noted in methylated tumors. (**B**) Backbone demethylation is associated with higher histological grade, squamous cell carcinoma histology, frequent lymphovascular invasion, and history of fewer abortions. Methylation, RNA expression and miRNA clusters are significantly associated.

Figure S12

Correlation of methylation with clinical, pathological and molecular variables in colorectal adenocarcinoma (COADREAD). (**A**) High CGI methylation is correlated with low N stage, low pathological stage, proximal location (for further illustration on anatomical location, see Supplementary Figure S25A), negative MSH6/PMS2 stain, absence of non-nodal tumor deposit, and high microsatellite instability (MSI-H). Gain of chromosomes 18 and 8p23 are frequently noted in methylated tumors. Low activities of RXR/RAR, interleukin-6 and ErbB4 pathways are noted with increasing methylation. (**B**) Backbone demethylation is not correlated significantly with any clinical parameters. Methylation, miRNA and SCNA clusters are significantly associated. BMP receptor and BCR pathways are frequently suppressed in demethylated tumors.

Figure S13

Correlation of methylation with clinical, pathological and molecular variables in stomach adenocarcinoma (STAD). (**A**) Higher CGI methylation is associated with high histological grade, negative helicobacter infection, better response to primary treatment and MSI-H status. Those with very high CGI methylation were not MSI-H and supposed to be Epstein-Barr virus type suggested by a previous TCGA study. CGI-methylated tumors have low number of SCNAs and near-diploidy, with frequent loss of 18p and gain of 19p and 9p. Those tumors have high number of mutations, especially in *PIK3CA*, *ARID1A*, *CBWD1*, *PGM5* and *PTEN*. Low activities of Ret, Reelin, $p75^{NTR}$ and calcium/CD4+TCR pathways are noted with increasing methylation. (**B**) Backbone demethylation is associated with low histological grade, non-diffuse type and low pathological stage. All molecular clusters are significantly associated. Demethylated tumors have higher number of SCNAs and increased ploidy, with frequent loss of 19p, 17p and 5q11 and gain of chromosomes 8q, 20 and 1q21. *TP53*-mutated tumors are more demethylated. Low activities of c-Kit, FoxM1 and p53 pathways are noted with decreasing methylation.

Figure S14

Correlation of methylation with clinical, pathological and molecular variables in breast invasive carcinoma (BRCA). (**A**) High CGI methylation is correlated with high frequency of estrogen receptor and HER2/neu expression, and accordingly, with luminal B subtype. All molecular clusters are significantly associated. CGI-methylated tumors tend to have high number of SCNAs, with frequent loss of chromosomes 11, 1p and 6q and gain of 17q23 and 8p11. Low activities of interleukin-23, LPA receptor, glucocorticoid receptor, EGFR and neurotrophic factor pathways are noted with increasing methylation. (**B**) Backbone demethylation is associated with ductal breast carcinoma and advanced stages. All molecular clusters are significantly associated. Demethylated tumors show high number of SCNAs, with frequent gain of chromosomes 20 and 16p and loss of 8p. They also have higher mutation rate and low activities of calcineurin/NFAT, EGFR and Ephrin B pathways.

Figure S15

Correlation of methylation with clinical, pathological and molecular variables in lung adenocarcinoma (LUAD). (**A**) High CGI methylation is correlated with advanced T stage but good response to primary treatment. Methylation, mRNA and SCNA clusters were significantly correlated. CGI-methylated tumors frequently have loss of chromosomes 6 and 20p and low activities of interleukin-6, reelin and BMP receptor pathways. (**B**) Backbone

demethylation is associated with smoking history. All molecular clusters are significantly associated. Demethylated tumors have higher number of SCNAs, higher ploidy, with frequent loss of 19p, 5q, 4q, 9q, 22q and 3p21 and gain of chromosomes 20, 6p, 8q and 12q24. Low activities of Fc-epsilon receptor I and FoxM pathways and high activities of PLK1 and Aurora B pathways are found with decreasing methylation.

Figure S16

Correlation of methylation with clinical, pathological and molecular variables in glioblastoma multiforme (GBM). (**A**) Associations of CGI methylation with methylation and SCNA clusters are observed. High CGI methylation is correlated with loss of chromosome 10 and gain of chromosomes 19 and 7p11. Mutation rates are high in methylated tumors. No pathway was significant after adjusting for multiple testing. (**B**) No significant association between backbone demethylation and clinical parameters is noted. Methylation, mRNA expression and SCNA clusters are significantly associated. Information on miRNA was unavailable from TCGA. Demethylated tumors tend to have loss of chromosomes 10, 15q, 19q and 13q and gain of 3q. *NF1* mutations are found in less demethylated tumors. No pathway was significant after adjusting for multiple testing.

Figure S17

Correlation of methylation with clinical, pathological and molecular variables in head and neck squamous cell carcinoma (HNSC). (**A**) High CGI methylation is correlated with advanced N stage and upstream location of oropharangeal tract (see Supplementary Figure S25B for overview on anatomical location). CGI-methylated tumors have frequent gain of chromosome 9 and CASP8 and EPHA2 mutations. Low activities of glucocorticoid receptor, Ephrin B and neurotrophic factor pathways are noted with increasing methylation. (**B**) Backbone demethylation is borderline associated with advanced pathological stage and less perineural invasion. All molecular clusters are significantly associated. Demethylated tumors tend to have high number of SCNAs, with frequent loss of 9p, 5q, 3p, 13q12 and 18q23. They also have slightly higher mutation rate, with very significant association of *NSD1* mutation. Low activity of SMAD2/3 pathway is noted with decreasing methylation.

Figure S18

Correlation of methylation with clinical, pathological and molecular variables in adrenocortical carcinoma (ACC). (**A**) High CGI methylation is associated with advanced pathological stage, high mitotic rate and poor response to primary treatment. CGI-

methylated tumors have higher number of SCNAs, with frequent loss of chromosomes 17 and 22q. Mutation rates are also high in methylated tumors. No pathway was significant after correction. (**B**) Backbone demethylation is associated with high Weiss score. No pathway was significant after correction.

Figure S19

Correlation of methylation with clinical, pathological and molecular variables in uterine corpus endometrial carcinoma (UCEC). (**A**) High CGI methylation is correlated with non-serous histology and MSI-H status. All molecular clusters are significantly associated. CGI-methylated tumors tend to have low number of SCNAs, with the absence of chromosomal abnormalities. They have higher mutation rate, with frequent *PTEN* and *PIK3R1* mutations and infrequent *TP53* mutation. Low activities of IGF/insulin, reelin and Ephrin B pathways are noted with increasing methylation. (**B**) Backbone demethylation is associated with endometrioid histology. Methylation and miRNA clusters are correlated. Low activities of osteopontin and LPA receptor pathways are noted with decreasing methylation.

Figure S20

Correlation of methylation with clinical, pathological and molecular variables in skin cutaneous melanoma (SKCM). (**A**) High CGI methylation is marginally associated with M0 stage. CGI-methylated tumors frequently have gain of chromosomes 19 and 5q35 and low activities of HIF-1-alpha and FoxA2/FoxA3 pathways. (**B**) Backbone demethylation is marginally associated with M1 stage. All molecular clusters are significantly associated. Demethylated tumors tend to have high number of SCNAs and high ploidy, with frequent loss of chromosomes 9, 10q and 5q. Low activities of S1P1, BCR and LPA receptor pathways are noted with decreasing methylation.

Figure S21

Correlation of methylation with clinical, pathological and molecular variables in lung squamous cell carcinoma (LUSC). (**A**) High CGI methylation is correlated with gain of chromosomes 9, 2p, 17q, 19p and 1q21. Methylation, RNA expression and SCNA clusters are significantly associated. Low activity of TRAIL pathway and high activity of E-cadherin pathway are noted with increasing methylation. (**B**) Backbone demethylation is associated with high Eastern Cooperative Oncology Group (ECOG) performance grade (poor activity). All molecular clusters are significantly associated. Demethylated tumors tend to have high number of SCNAs, with frequent loss of chromosomes 9, 4p, 13q and 5q11. Low activities of

HIF-1-alpha, calcineurin/NFAT and calcium/CD4+TCR pathways are noted with decreasing methylation.

Figure S22

Correlation of methylation with clinical, pathological and molecular variables in bladder urothelial carcinoma (BLCA). (**A**) High CGI methylation is correlated with high histological grade. Gain of 5p and 18p are frequent and *FGFR3* and *STAG2* mutations are infrequent in CGI-methylated tumors. No pathway was significant after correction for multiple testing. (**B**) Backbone demethylation is associated with less extra-capsular extension. All molecular clusters are significantly associated. Demethylated tumors tend to have high number of SCNAs, with frequent loss of chromosomes 9, 11 and 5q and gain of 7q and 8q. They have low activities of interleukin-23, calcineurin/NFAT, BCR and LPA receptor pathways.

Figure S23

Correlation of methylation with clinical, pathological and molecular variables in uterine carcinosarcoma (UCS). (**A**) CGI methylation was not apparent in UCS and the correlation with CGI methylation was negligible. (**B**) Backbone demethylation is associated with high percentage of tumor invasion and advanced clinical stage. Methylation and mRNA expression clusters are significantly associated. No pathway was significant after correction.

Figure S24

Correlation of methylation with clinical, pathological and molecular variables in liver hepatocellular carcinoma (LIHC). (**A**) CGI methylation is associated with methylation, mRNA expression and miRNA clusters. CGI-methylated tumors frequently have gain of 17p and 19p and loss of 1p36. Low activities of syndecan-4 and HIF-1-alpha pathways are noted with increasing methylation. (**B**) No clinical parameter was significantly associated with backbone demethylation. Rather, all molecular clusters are significantly associated. Demethylated tumors tend to have high number of SCNAs, with frequent loss of 17p and 8p. Low activities of Ephrin B, PDGFR-alpha, BMP receptor and LPA receptor pathways are noted with decreasing methylation.

Figure S25

CGI methylation according to anatomical location. (**A**) CGI methylation is the highest in cecum (number 1) and the median methylation levels gradually decrease along with the

direction of food passage in lower digestive tract. (**B**) CGI methylation is highest in oral cavity and decreases in the caudal direction in the oropharangeal tract.

Figure S26

Correlation of between SCNAs and CGI methylation according to chromosomal loci. By concatenating gene-level associations, -log*P* values (x-axis) are plotted according to chromosomal location. Color gradients are according to the 10th, 20th, 30th and >40th percentiles of the 21 tumor types.

Figure S27

Correlation of between SCNAs and backbone methylation according to chromosomal loci. By concatenating gene-level associations, -log*P* values (x-axis) are plotted according to chromosomal location. Color gradients are according to the percentiles of 21 tumor types.

Figure S28

Loci showing significant correlation of copy number with methylation. (**A**) 19p region showing recurrent association with backbone demethylation. (**B**) 19p region showing recurrent association with CGI methylation. (**C**) Copy loss of an exemplary gene (*SIRT6*) at 19p13.3 correlates significantly with backbone demethylation. All types of tumors are analyzed and copy-neutral tumors are excluded from the plot. (**D**) Copy gain of an exemplary gene (*SIRT6*) at 19p13.3 correlates significantly with CGI methylation.

Figure S29

Correlation of methylation and expression according to genic location of each CpG. CGI methylation around promoter and 5´-body is negatively correlated with gene expression in all tumor types. In many tumors, backbone methylation level is positively correlated with gene expression, indicating that demethylation is related with low gene expression. Correlation coefficients are plotted by local regression.

Figure S30

TF enrichment of *de novo* methylated CpGs in 12 tumor types. (**A**) Among the 161 ENCODE TFs, binding sites of polycomb proteins, SUZ12 and EZH2, and polycomb-associated CTBP2 are enriched in all tumor types. (**B**) Enrichment rates of SUZ12, EZH2 and CTBP2 are usually correlated with the degree of CGI methylation.

Figure S31

TF enrichment of demethylated CpGs in 12 tumor types. (**A**) Among the 161 ENCODE TFs, binding sites of IKZF1, BATF and ZNF217 are most commonly enriched but the enrichments are different according to tumor types. (**B**) Enrichment rates of IKZF1, BATF and ZNF217 are not correlated with backbone demethylation.

Figure S32

CGI methylation inside and outside of lamina-associated domains (LADs). *De novo* methylation of CGI is predominantly inside LADs. The trend is subtle in relatively silent tumors like THCA and KICH.

Figure S33

Backbone methylation inside and outside of LADs. Decreased levels of backbone is predominantly inside LADs, especially in more demethylated tumors. The trend is subtle in less demethylated tumors like THCA, KICH, KIRP, KIRC, LAML and LGG.

Figure S34

Association with clinical outcome in methylation groups of THCA. (**A**) Normal and high CGI methylation groups (NC and HC, respectively) are defined by a cutoff of 0.24 for average CGI methylation. Normal and low backbone methylation groups (NB and LB, respectively) are defined by a cutoff of 0.78 for average backbone methylation. (**B** and **C**) Kaplan-Meier survival analyses show worse overall survival (OS) and progression-free survival (PFS) in HC and LB tumors (*P* values are generated from a log-rank test). (**D** and **E**) Cox proportional hazard (CPH) models show that both HC and LB tumors have high hazard ratios (HRs) but the association was insignificant after correcting other parameters like age and pathological stage. Due to the small number of subjects, exact HRs for HC-LB group could not be calculated and the group was excluded from the CPH models. Age was omitted in models 2 and 3, because staging in THCA incorporates age (n=476, 476, 474 and 384 for unadjusted and models 1, 2 and 3 in OS, respectively; n=391, 391, 390 and 306 for unadjusted and models 1, 2 and 3 in PFS, respectively).

Figure S35

Association with clinical outcome in methylation groups of KICH. (**A**) Distribution of HC and LB group tumors. (**B** and **C**) Kaplan-Meier survival analyses show poor OS and PFS in

HC-LB tumors. (**D** and **E**) CPH models show that HC-LB tumors show poor OS and PFS still after correcting other prognostic factors. However, this may not be conclusive due to the small number of subjects (n=66, 66, 66 and 52 for unadjusted and models 1, 2 and 3 in OS, respectively; n=49, 49, 49 and 35 for unadjusted and models 1, 2 and 3 in PFS, respectively).

Figure S36

Association with clinical outcome in methylation groups of KIRP. (**A**) Distribution of HC and LB group tumors. (**B** and **C**) Kaplan-Meier survival analyses show poor OS and PFS in HC tumors. (**D** and **E**) CPH models show that HC tumors show poor OS and PFS but it was insignificant after correcting other prognostic factors. Due to the small numbers, the HC-LB group was excluded from the model (n=148, 142, 142, and 107 for unadjusted and models 1, 2 and 3 in OS, respectively; n=72, 72 and 72 for unadjusted and models 1 and 2 in PFS, respectively).

Figure S37

Association with clinical outcome in methylation groups of KIRC. (**A**) Distribution of HC and LB group tumors. (**B** and **C**) Kaplan-Meier survival analyses show poor OS and PFS in HC or LB tumors. (**D** and **E**) CPH models show that HC tumors show poor OS and PFS but it was insignificant after correcting other prognostic factors (n= 290, 290, 244 and 209 for unadjusted and models 1, 2 and 3 in OS, respectively; n=108, 108, 88 and 77 for unadjusted and models 1, 2 and 3 in PFS, respectively).

Figure S38

Association with clinical outcome in methylation groups of LGG. (**A**) Distribution of HC and LB group tumors. (**B** and **C**) Kaplan-Meier survival analyses show that HC-NB tumors show the most favorable prognosis and the others have worse OS and PFS. (**D** and **E**) CPH models show that LB or NC tumors show poor OS and PFS even when incorporating age, gender and histological type and grade. However, it was insignificant after incorporating mutations in *IDH1* and other genes (n=192, 192, 189 and 186 for unadjusted and models 1, 2 and 3 in OS, respectively; n=128, 128, 125 and 122 for unadjusted and models 1, 2 and 3 in PFS, respectively).

Figure S39

Association with clinical outcome in methylation groups of COADREAD. (**A**) Distribution of HC and LB group tumors. (**B** and **C**) Kaplan-Meier survival analyses show better survival in LB tumors. (**D** and **E**) CPH models show that LB tumors show better OS even when incorporating age, gender and stage. However, it was borderline after incorporating MSI status (n=368, 368, 355 and 339 for unadjusted and models 1, 2 and 3 in OS, respectively; n=297, 297, 293 and 277 for unadjusted and models 1, 2 and 3 in PFS, respectively).

Figure S40

Association with clinical outcome in methylation groups of GBM. (**A**) Distribution of HC and LB group tumors. (**B** and **C**) Kaplan-Meier survival analyses show that HC tumors show more favorable prognosis and LB have worse OS. (**D** and **E**) CPH models show that LB tumors show poor OS or PFS but it was insignificant after incorporating other factors (n=110, 110, 79 and 75 for unadjusted and models 1, 2 and 3 in OS, respectively; n=59, 59, 38 and 37 for unadjusted and models 1, 2 and 3 in PFS, respectively).

Figure S41

Association with clinical outcome in methylation groups of ACC. (**A**) Distribution of HC and LB group tumors. (**B** and **C**) Kaplan-Meier survival analyses show poor OS and PFS in HC tumors. (**D** and **E**) CPH models show that HC-LB tumors show poor PFS but it was insignificant after correcting other factors. The results may inconclusive due to the small number of subjects (n=74, 74, 69 and 68 for unadjusted and models 1, 2 and 3 in OS, respectively; n= 72, 72, 67 and 66 for unadjusted and models 1, 2 and 3 in PFS, respectively).

Supplementary Figure S1

A **B**

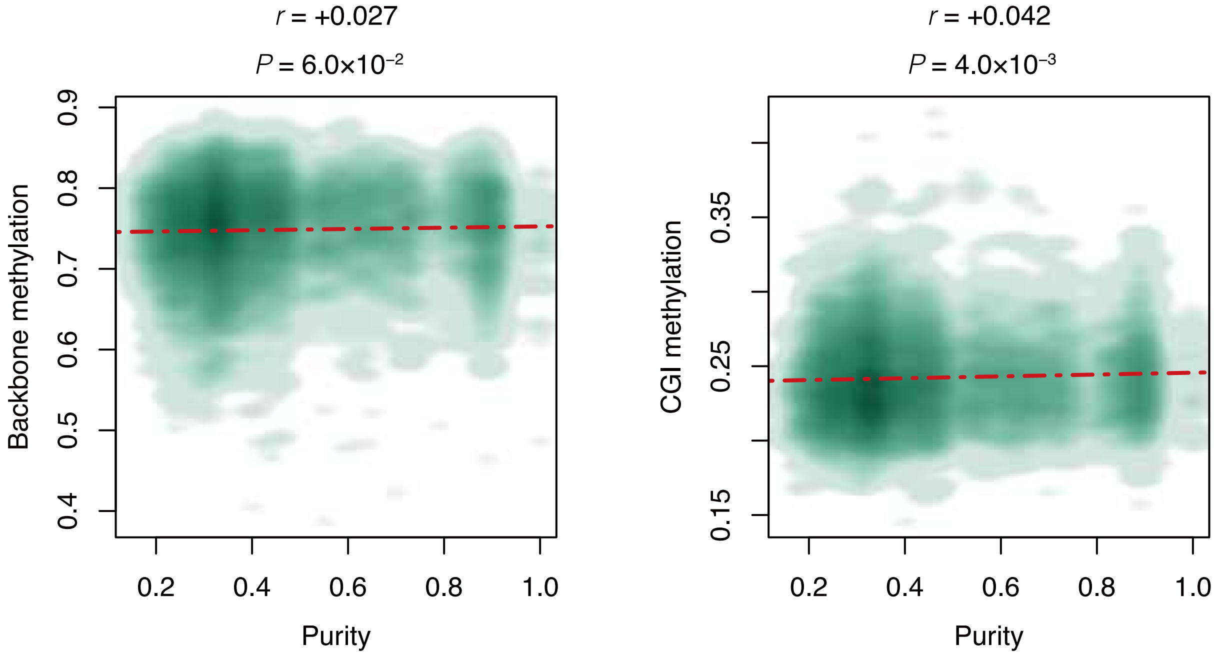

Supplementary Figure S2

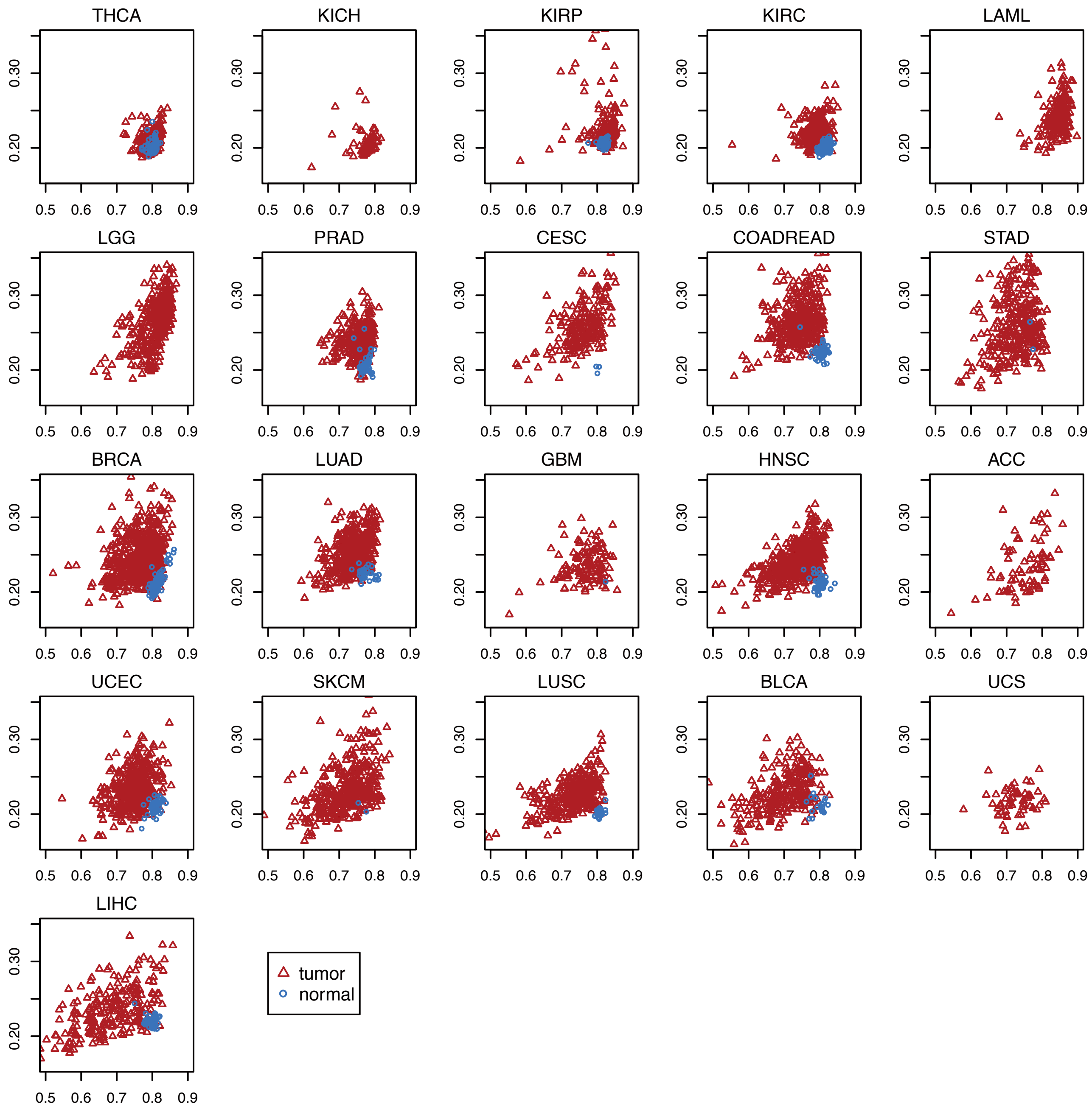

Supplementary Figure S3

A

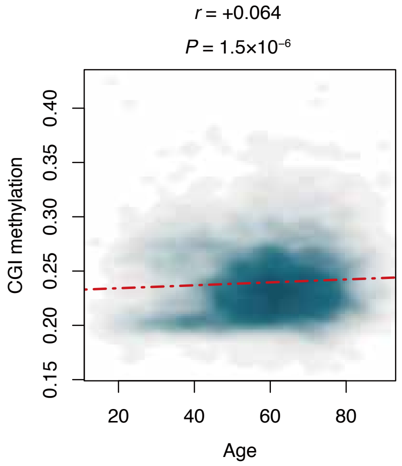

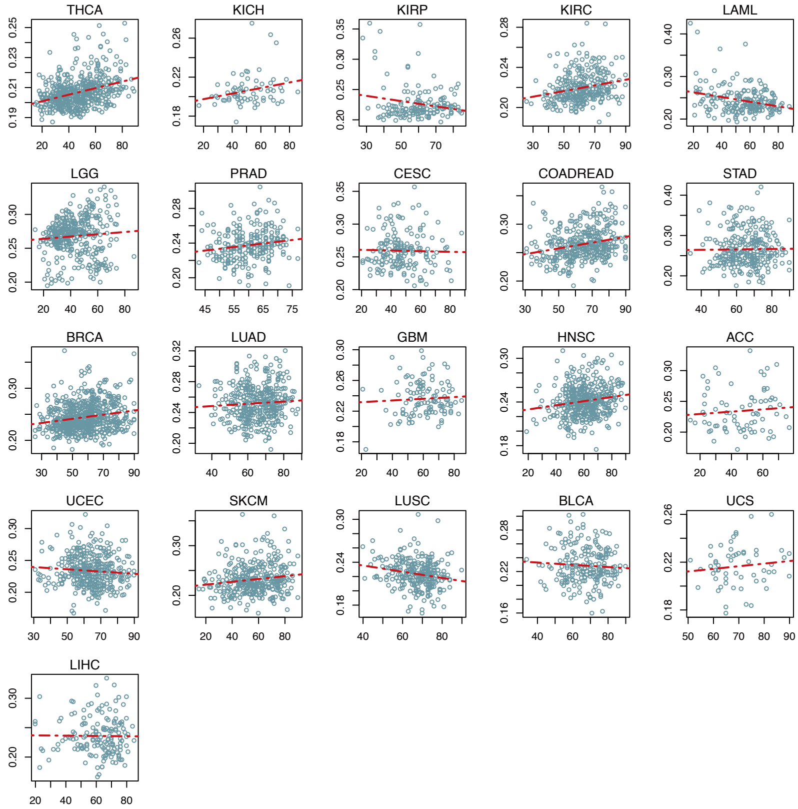

**Tumor type**	***r***	***P***
THCA	+0.346	4.0×10^{-15}
KICH	+0.250	4.3×10^{-2}
KIRP	−0.194	1.6×10^{-2}
KIRC	+0.228	9.0×10^{-5}
LAML	−0.279	8.0×10^{-5}
LGG	+0.082	1.2×10^{-1}
PRAD	+0.157	1.5×10^{-2}
CESC	−0.024	7.6×10^{-1}
COADREAD	+0.239	3.5×10^{-6}
STAD	+0.013	8.4×10^{-1}
BRCA	+0.198	2.7×10^{-7}
LUAD	+0.065	2.0×10^{-1}
GBM	+0.061	5.2×10^{-1}
HNSC	+0.167	6.3×10^{-4}
ACC	+0.088	4.6×10^{-1}
UCEC	−0.083	1.1×10^{-1}
SKCM	+0.146	6.7×10^{-3}
LUSC	−0.199	1.0×10^{-3}
BLCA	−0.066	3.4×10^{-1}
UCS	+0.119	3.8×10^{-1}
LIHC	−0.010	9.0×10^{-1}

B

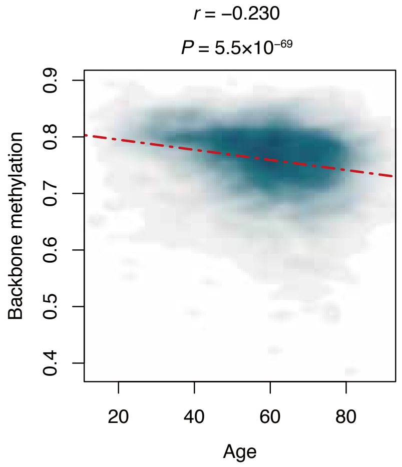

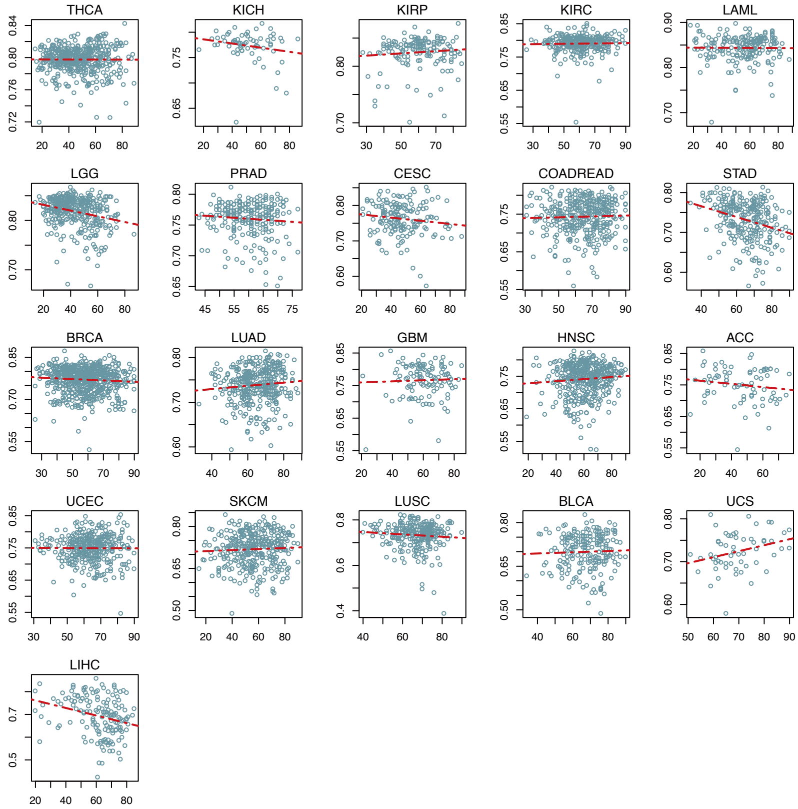

**Tumor type**	***r***	***P***
THCA	−0.004	9.3×10^{-1}
KICH	−0.192	1.2×10^{-1}
KIRP	+0.093	2.6×10^{-1}
KIRC	+0.024	6.8×10^{-1}
LAML	−0.008	9.1×10^{-1}
LGG	−0.262	3.5×10^{-7}
PRAD	−0.083	2.0×10^{-1}
CESC	−0.130	9.2×10^{-2}
COADREAD	+0.034	5.2×10^{-1}
STAD	−0.290	1.3×10^{-6}
BRCA	−0.075	5.2×10^{-2}
LUAD	+0.094	6.7×10^{-2}
GBM	+0.040	6.7×10^{-1}
HNSC	+0.079	1.1×10^{-1}
ACC	−0.145	2.2×10^{-1}
UCEC	−0.007	8.9×10^{-1}
SKCM	+0.050	3.6×10^{-1}
LUSC	−0.083	1.7×10^{-1}
BLCA	+0.037	6.0×10^{-1}
UCS	+0.294	2.6×10^{-2}
LIHC	−0.272	6.1×10^{-4}

Supplementary Figure S4

A

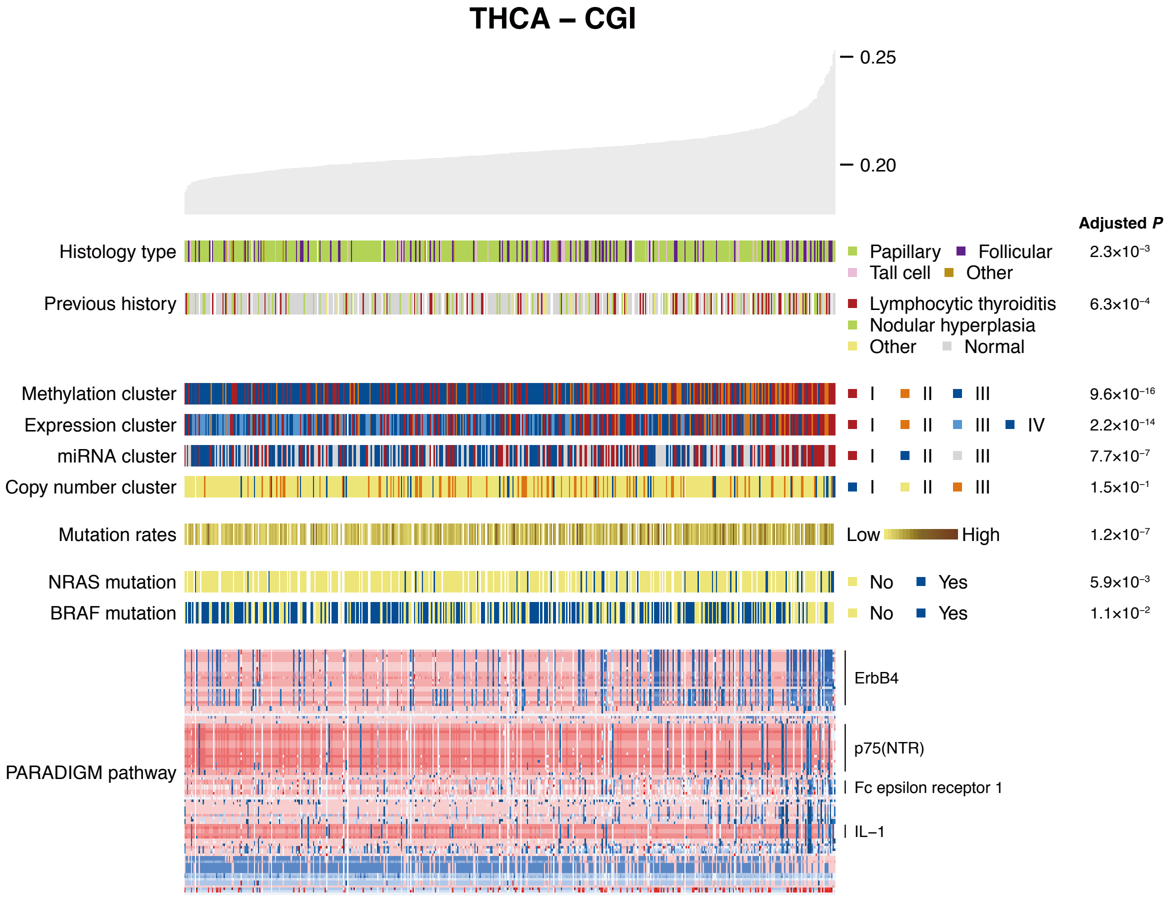

B

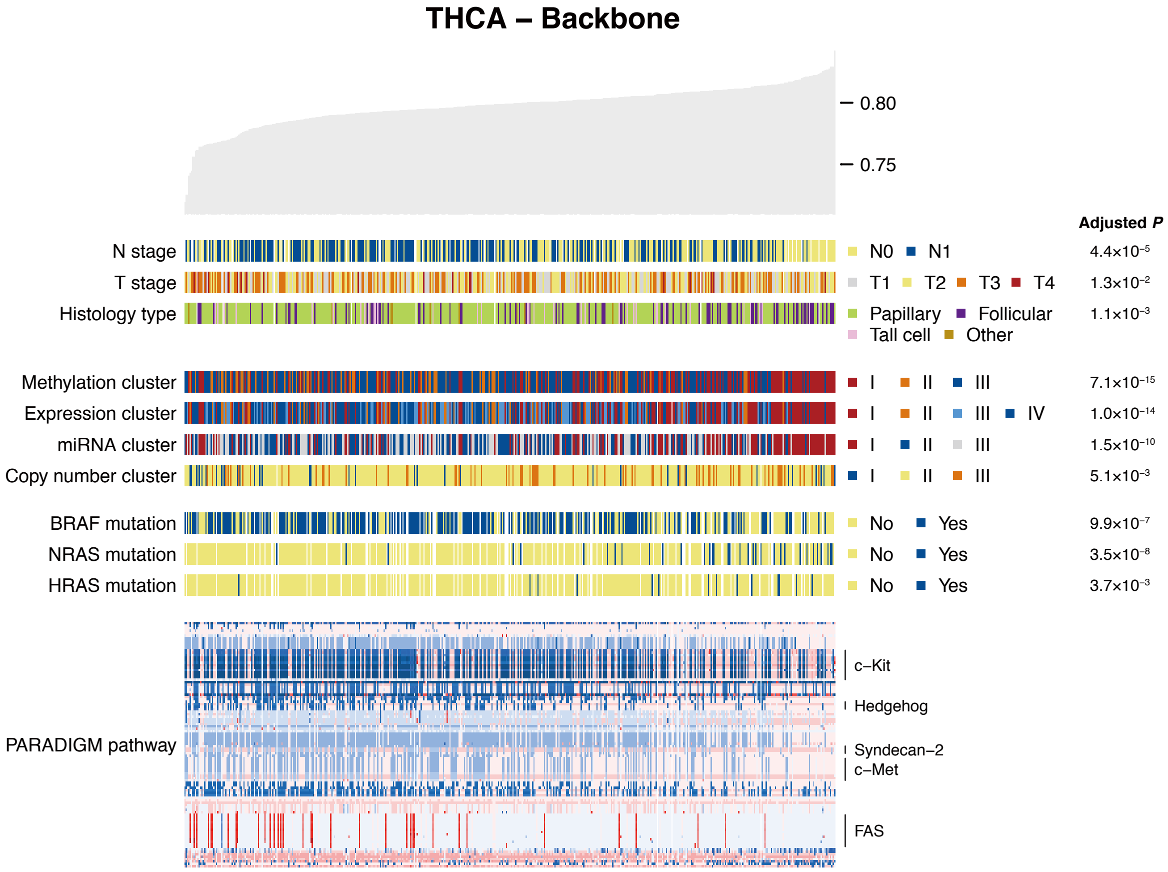

Supplementary Figure S5

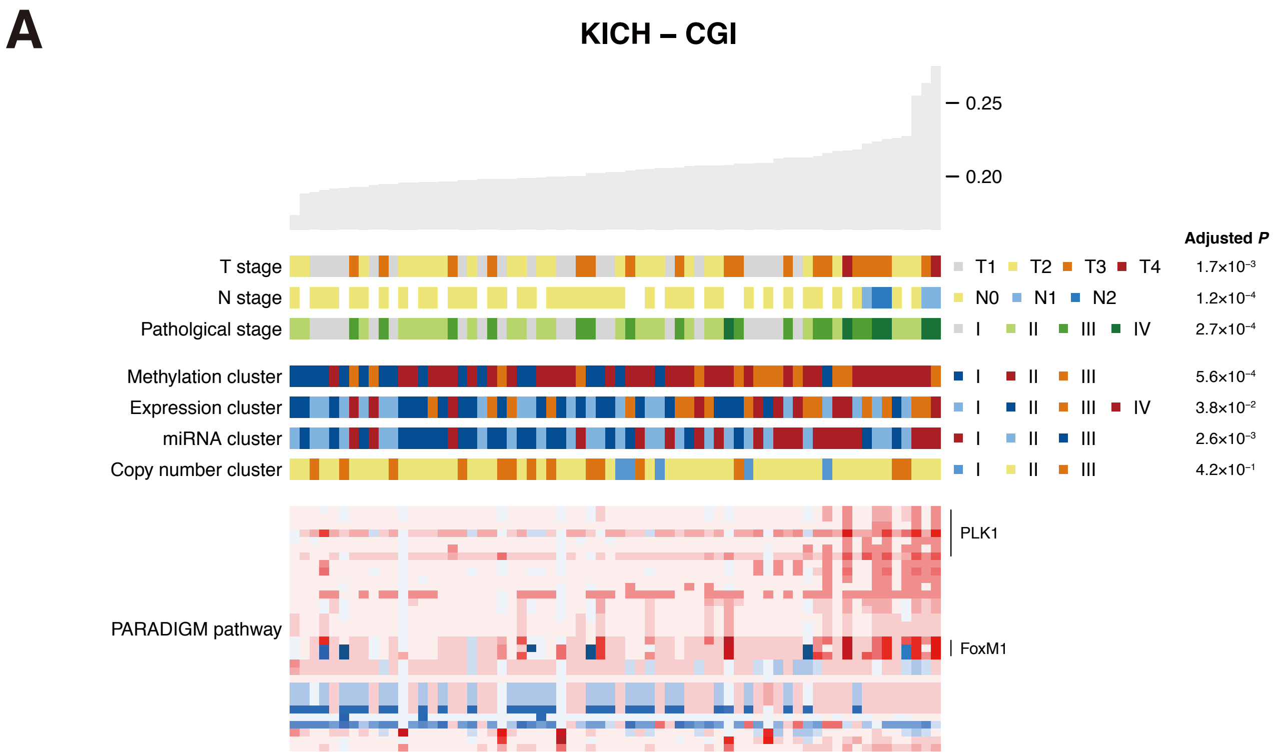

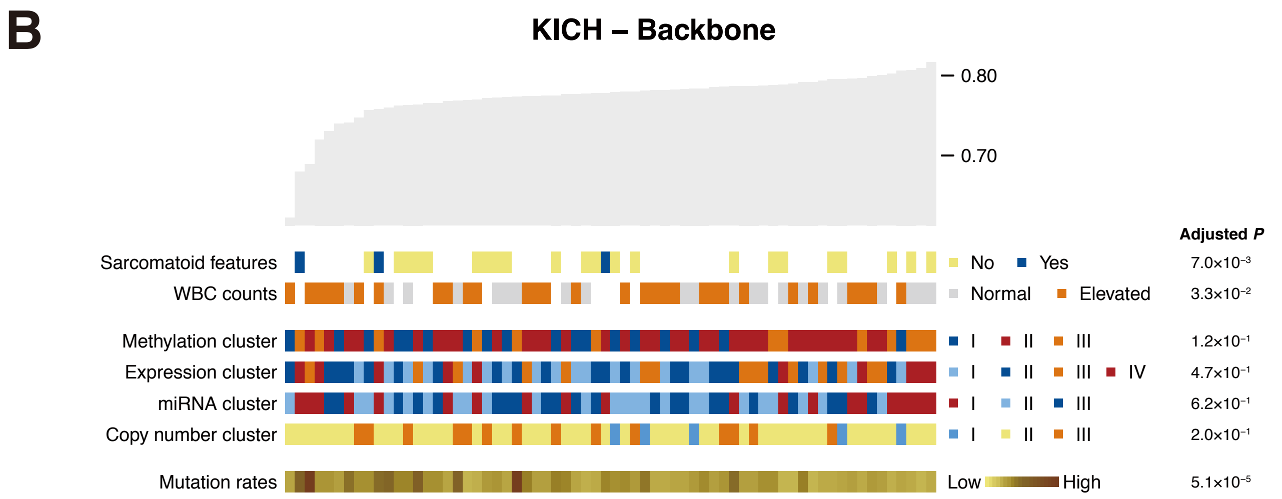

Supplementary Figure S6

A

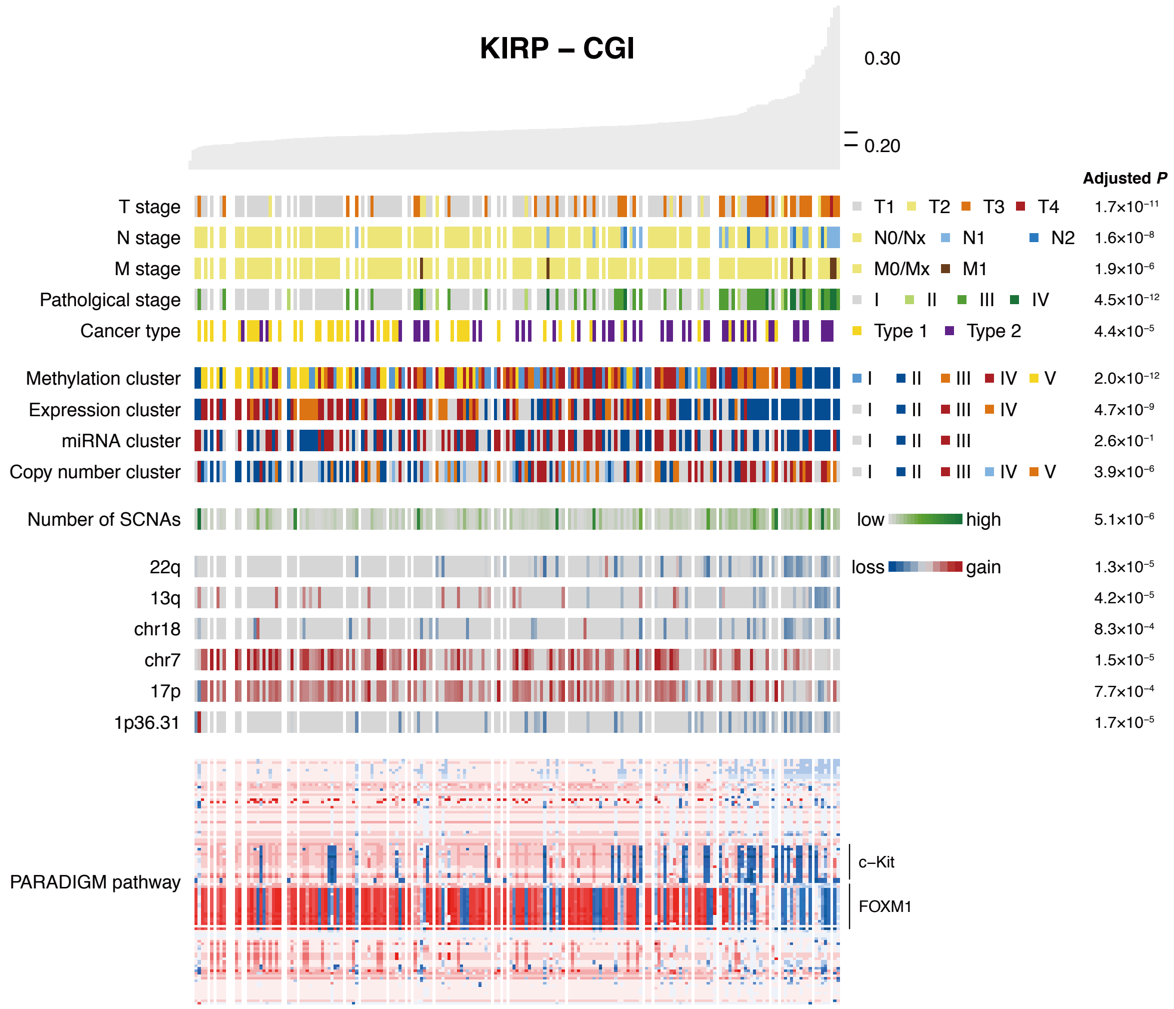

B

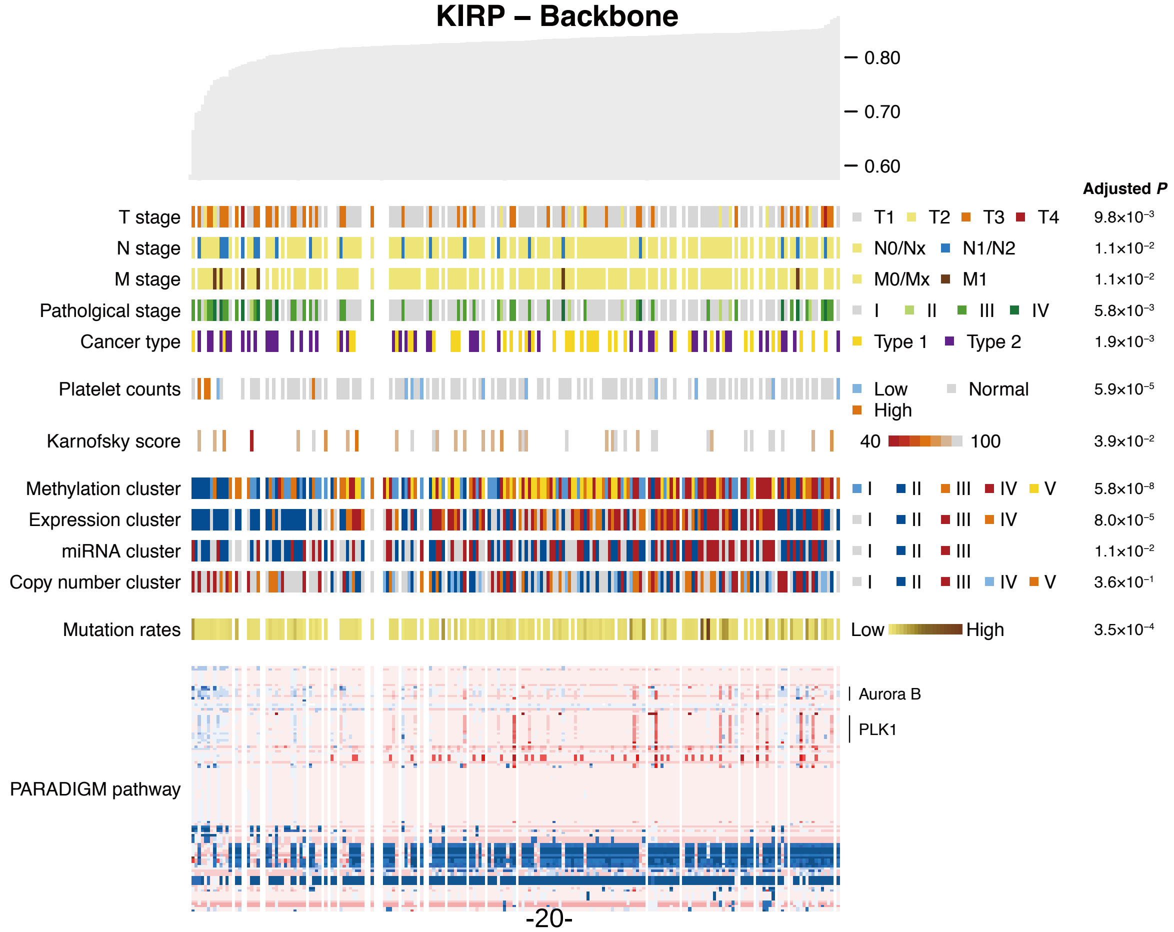

Supplementary Figure S7

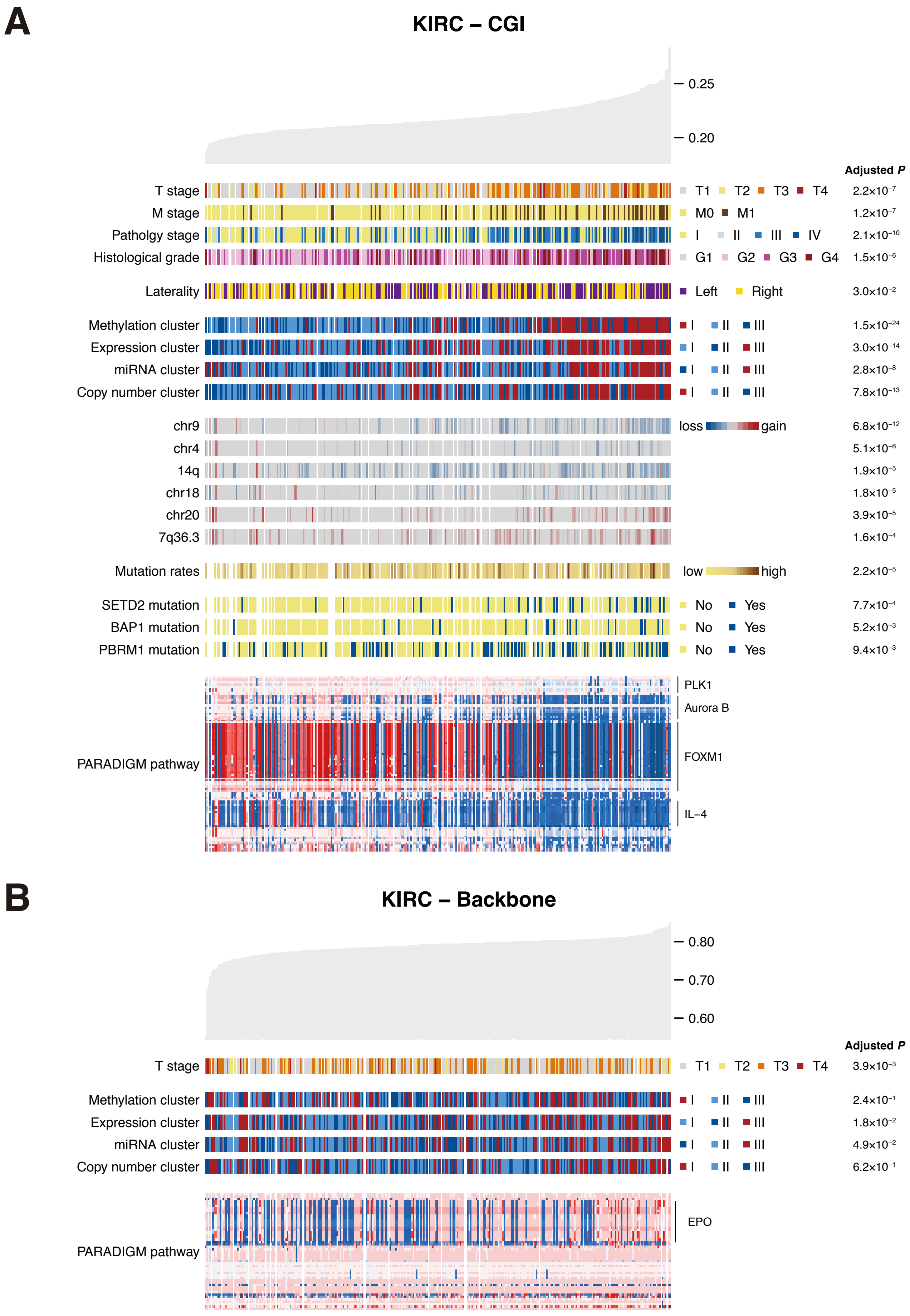

Supplementary Figure S8

A

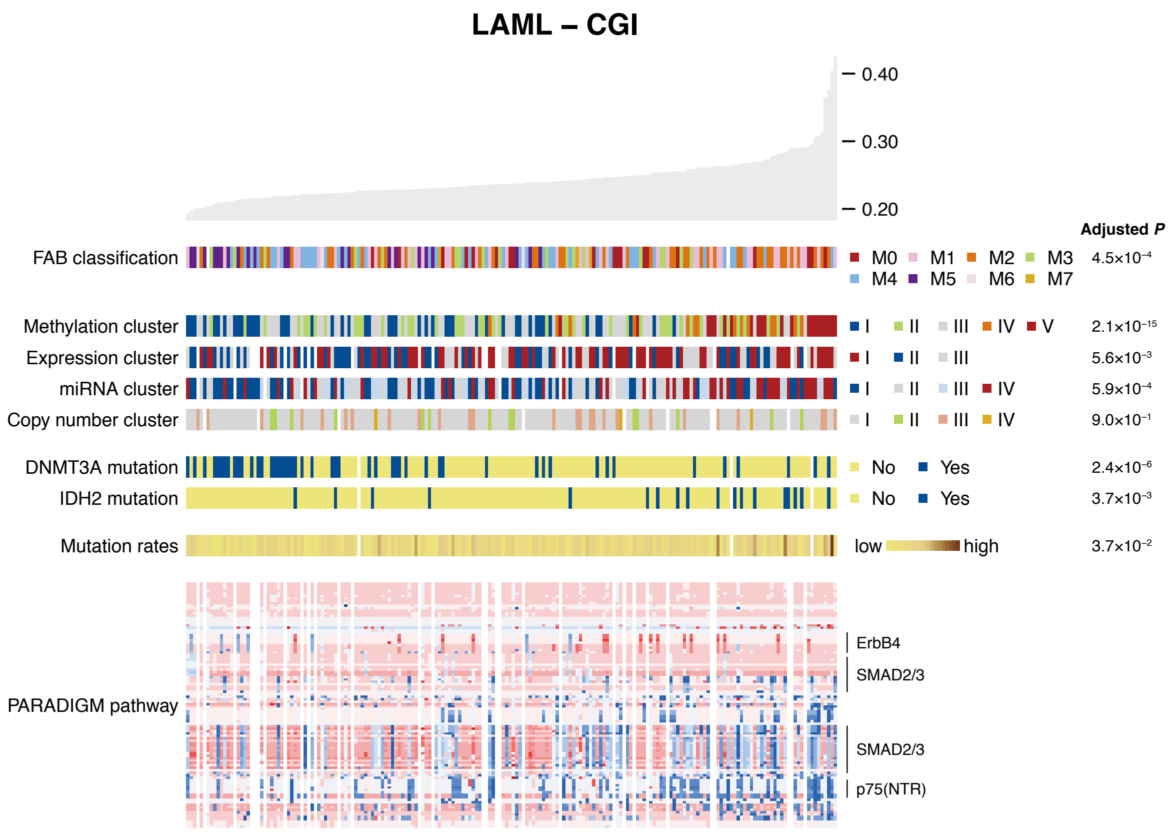

B

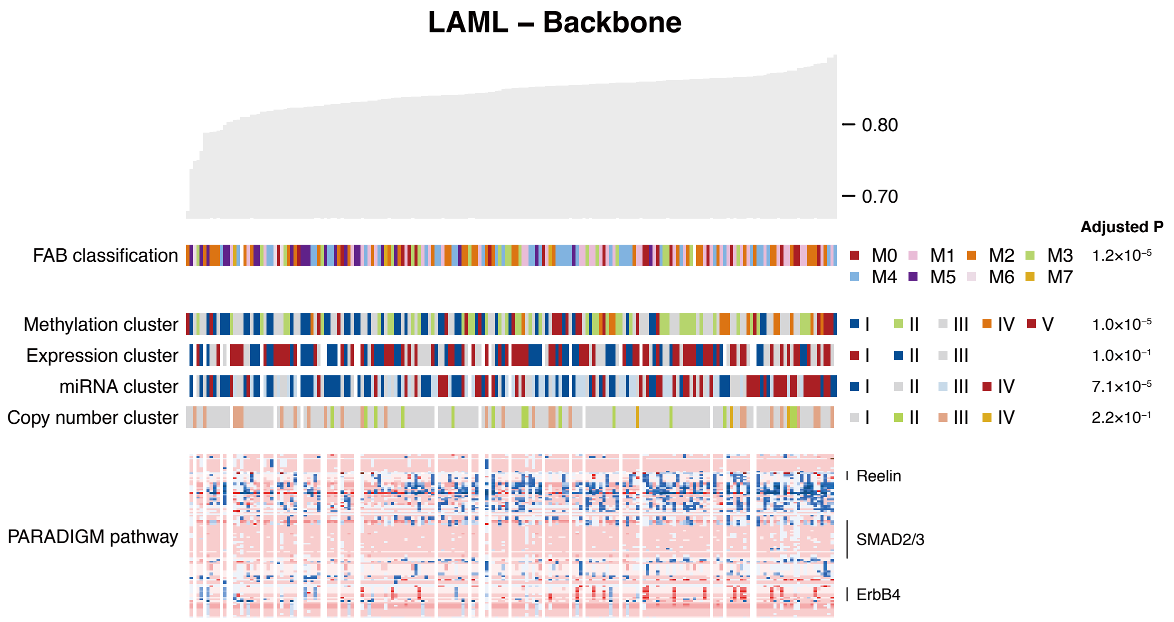

Supplementary Figure S9

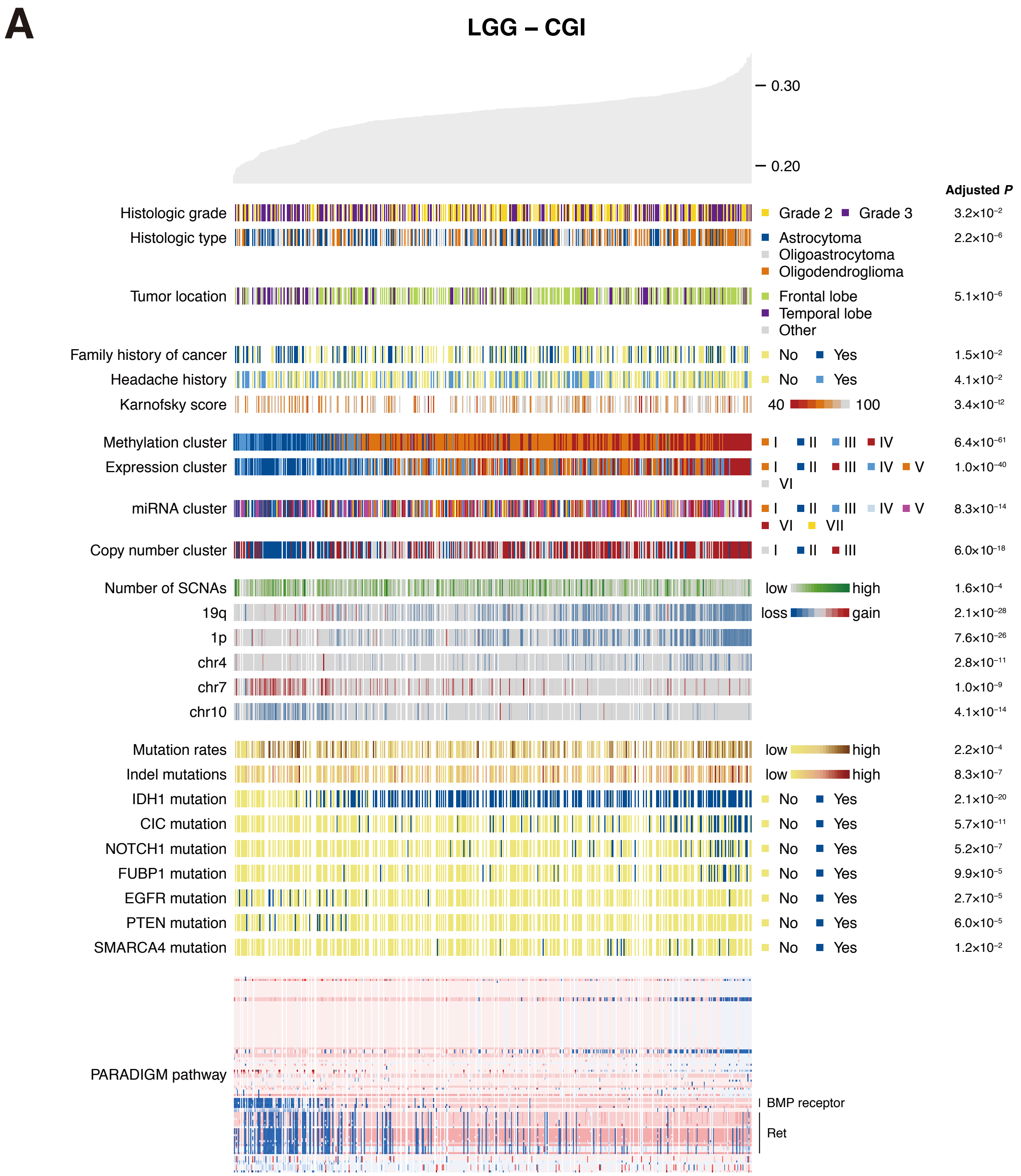

Supplementary Figure S9

B

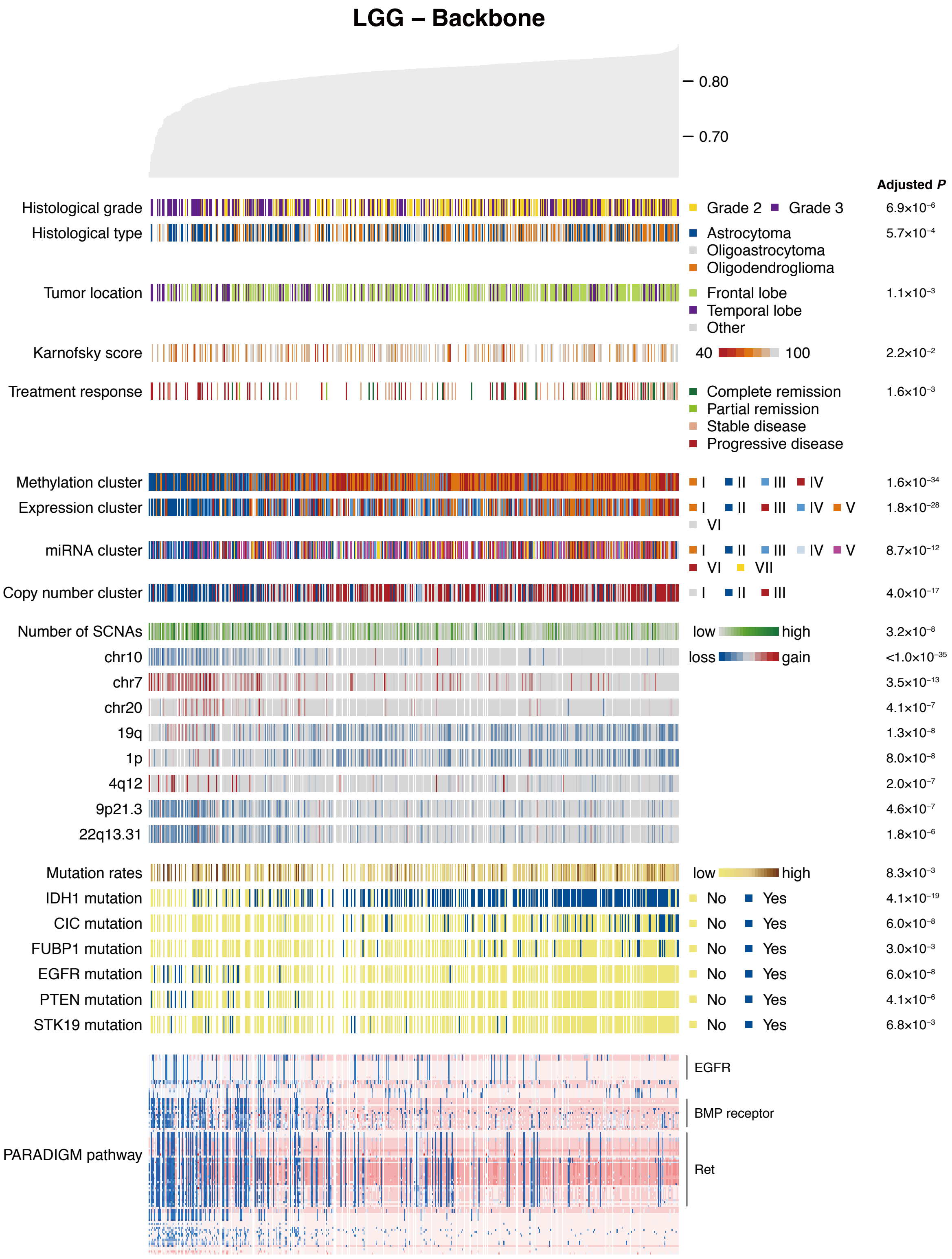

Supplementary Figure S10

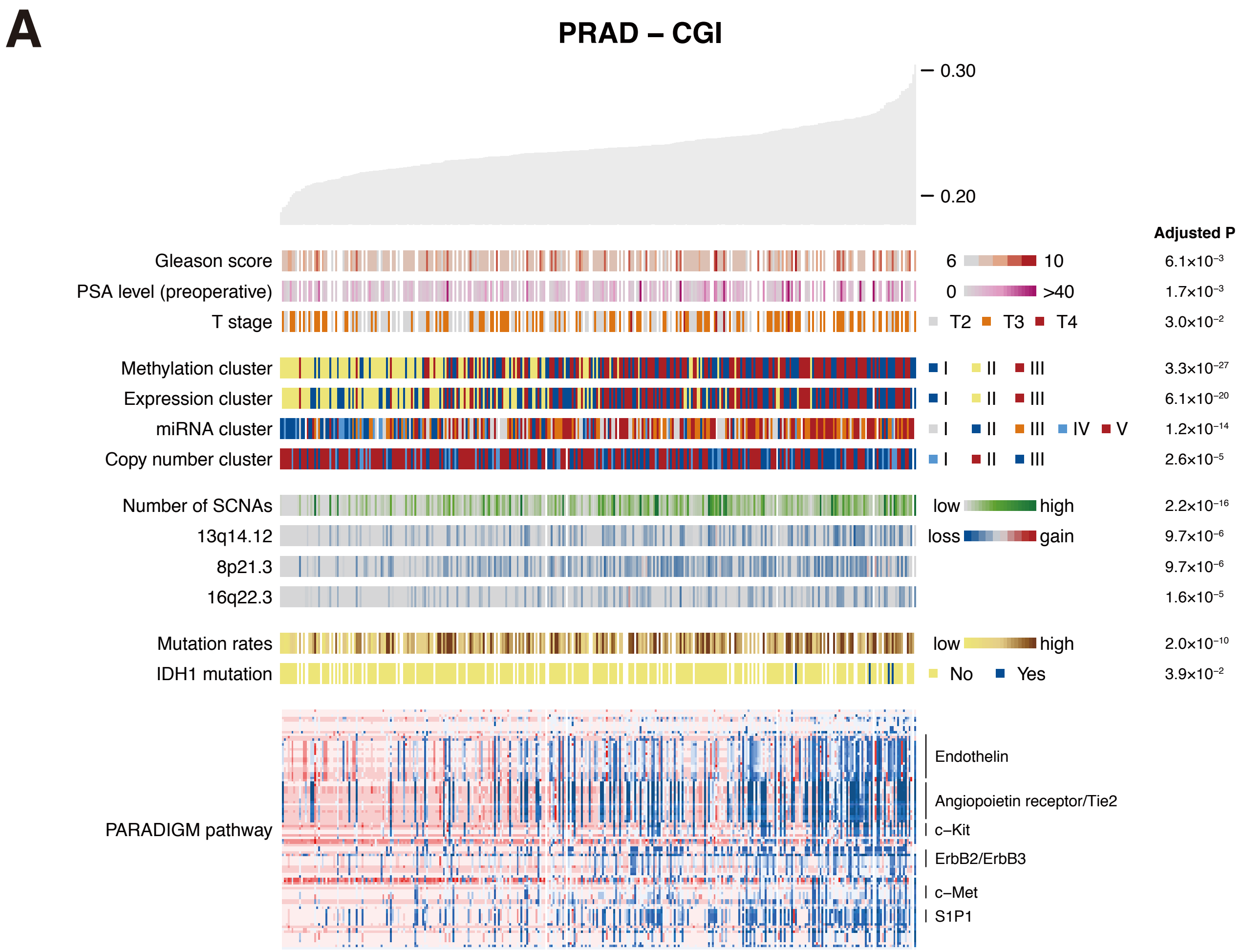

Supplementary Figure S10

B

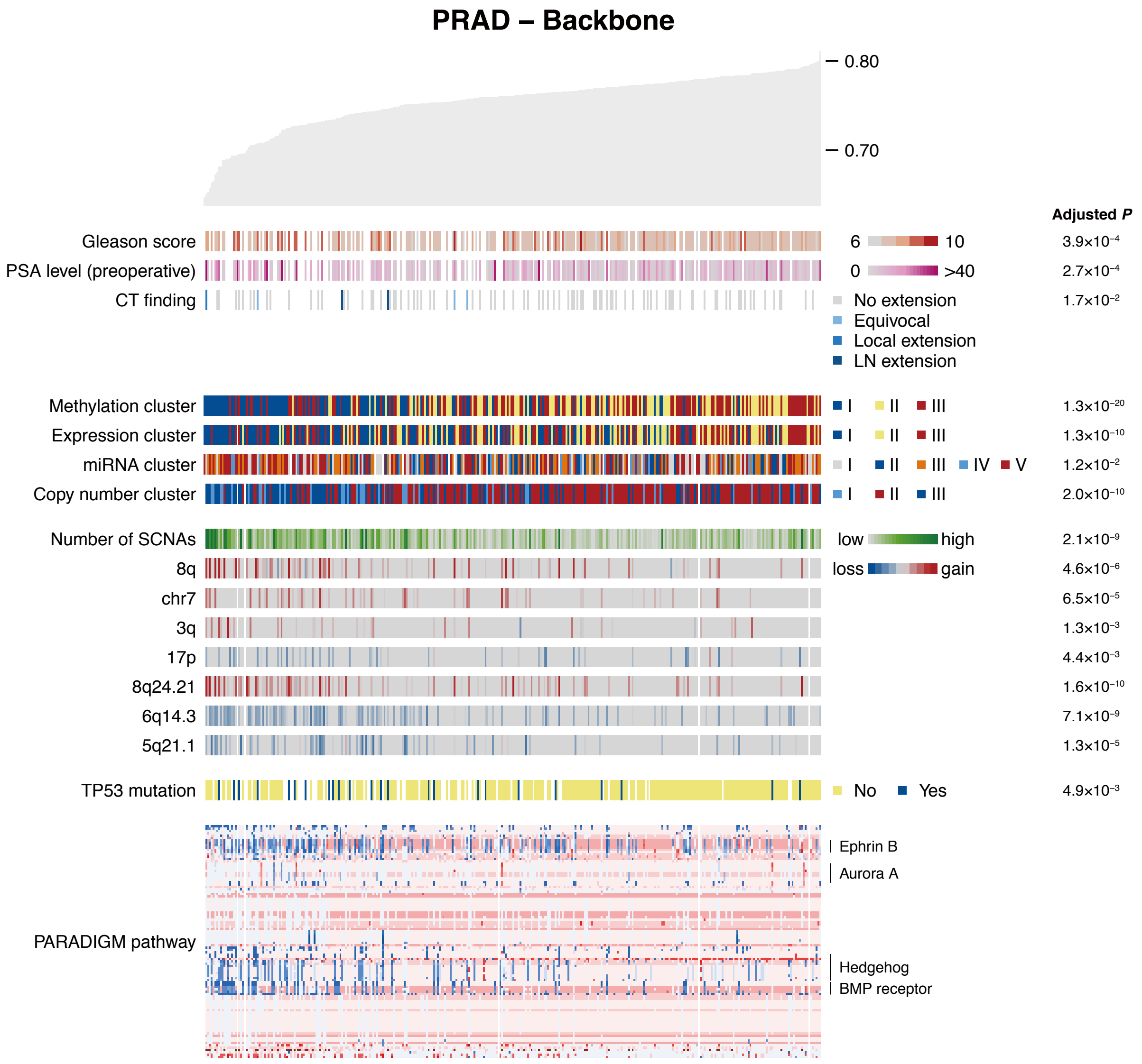

Supplementary Figure S11

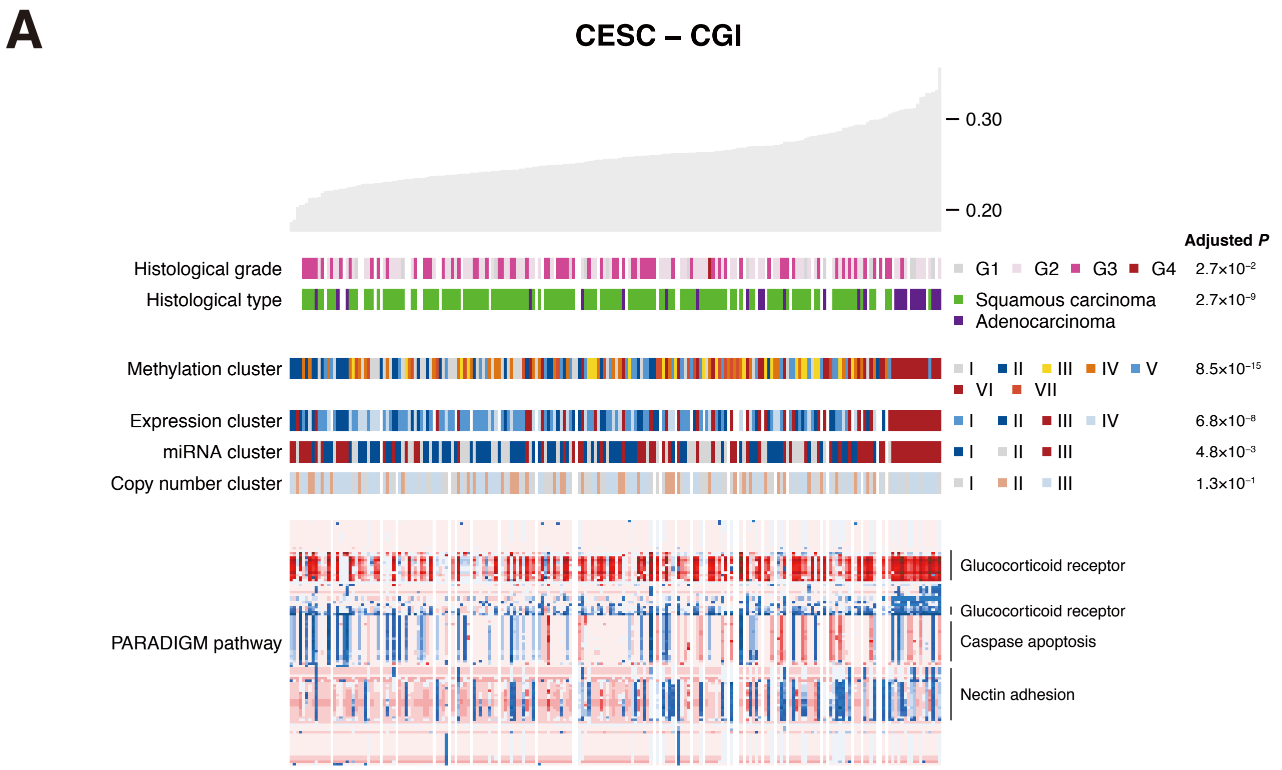

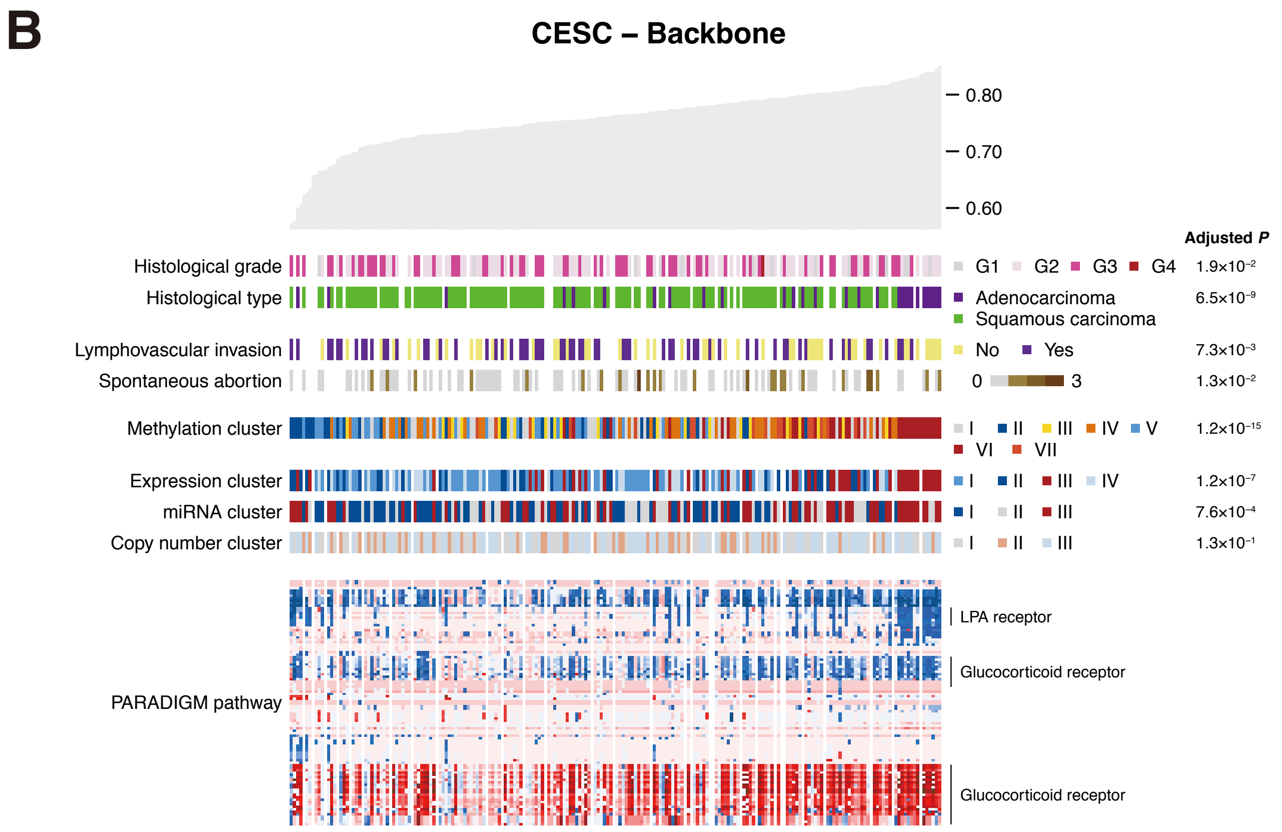

Supplementary Figure S12

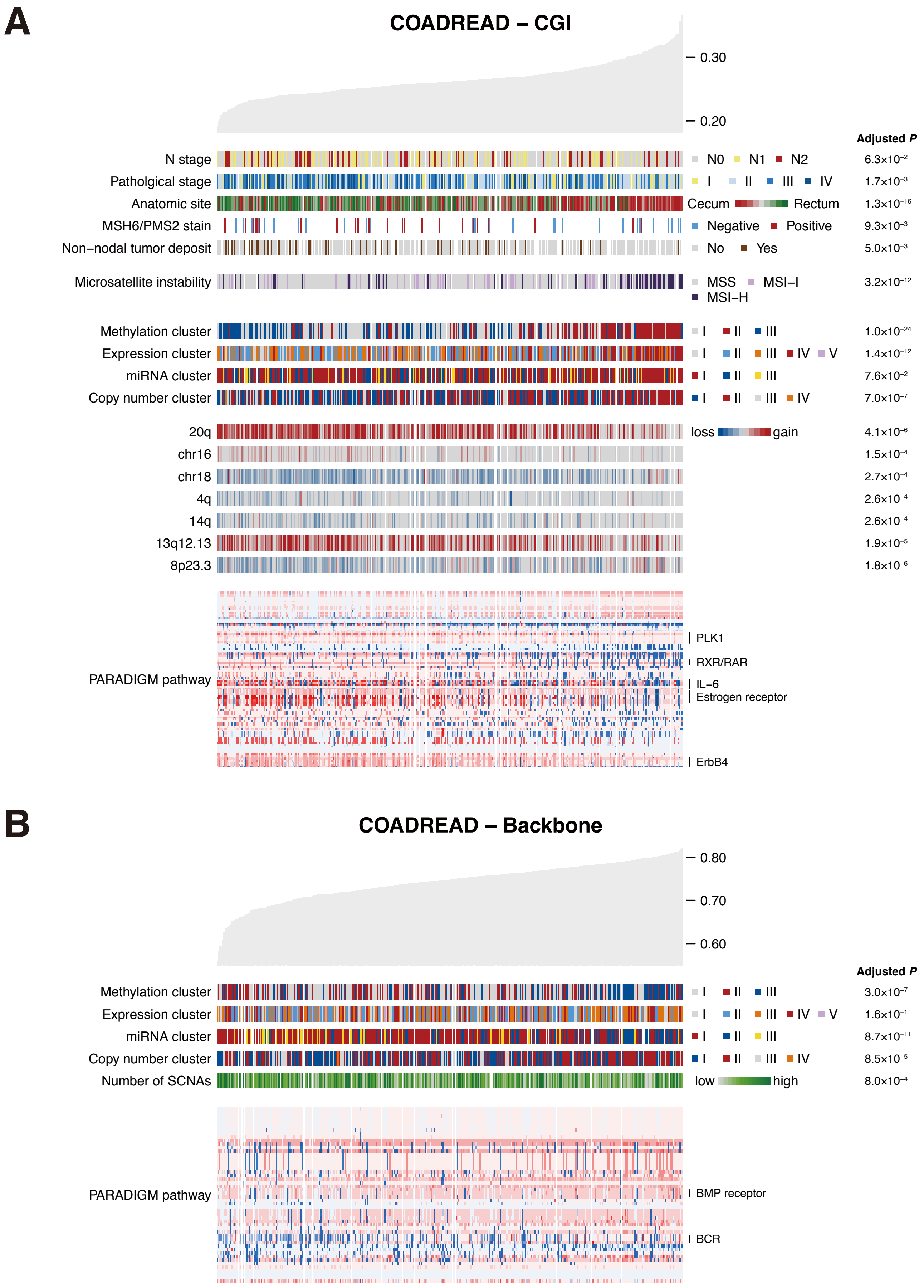

Supplementary Figure S13

A

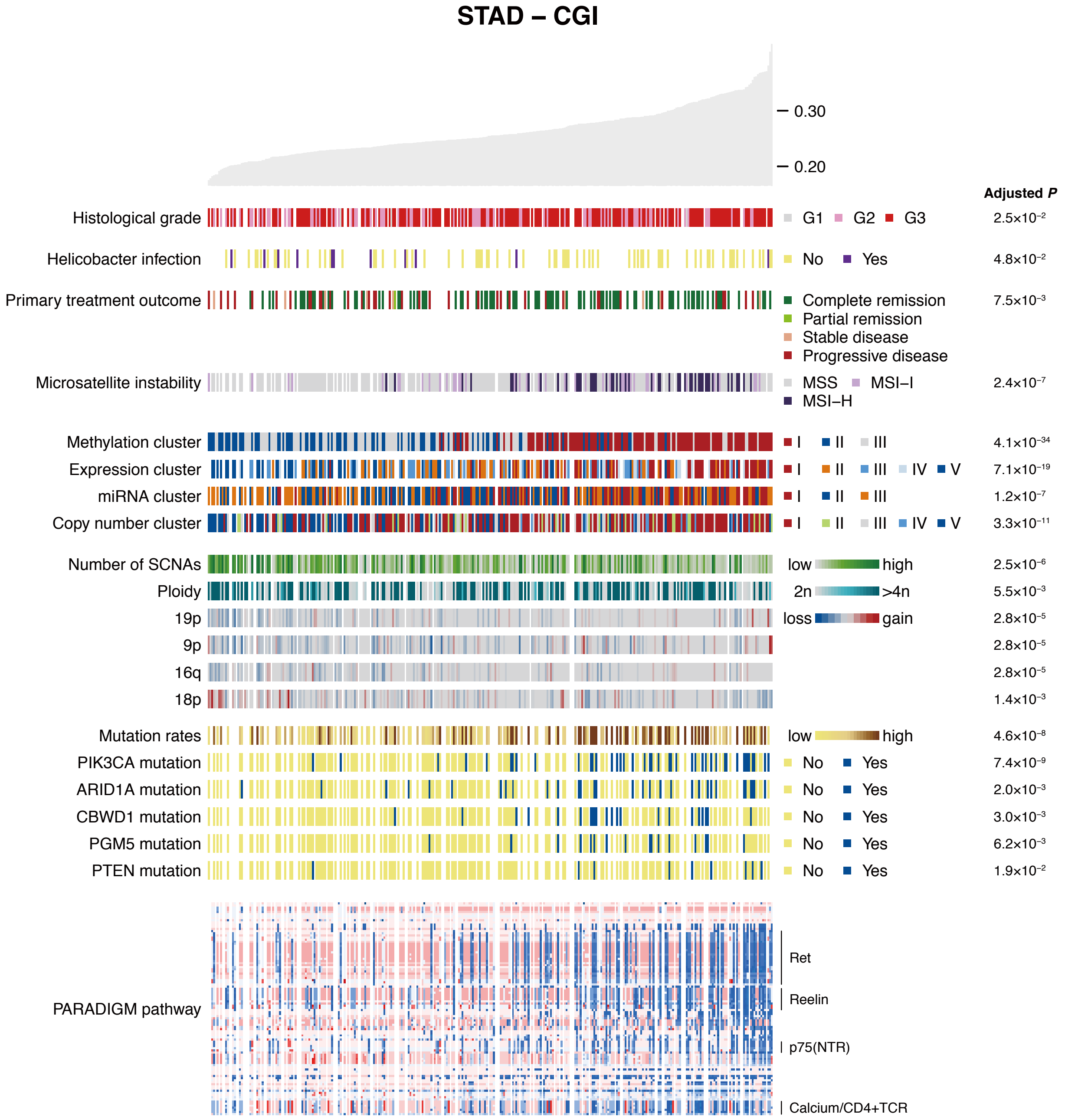

Supplementary Figure S13

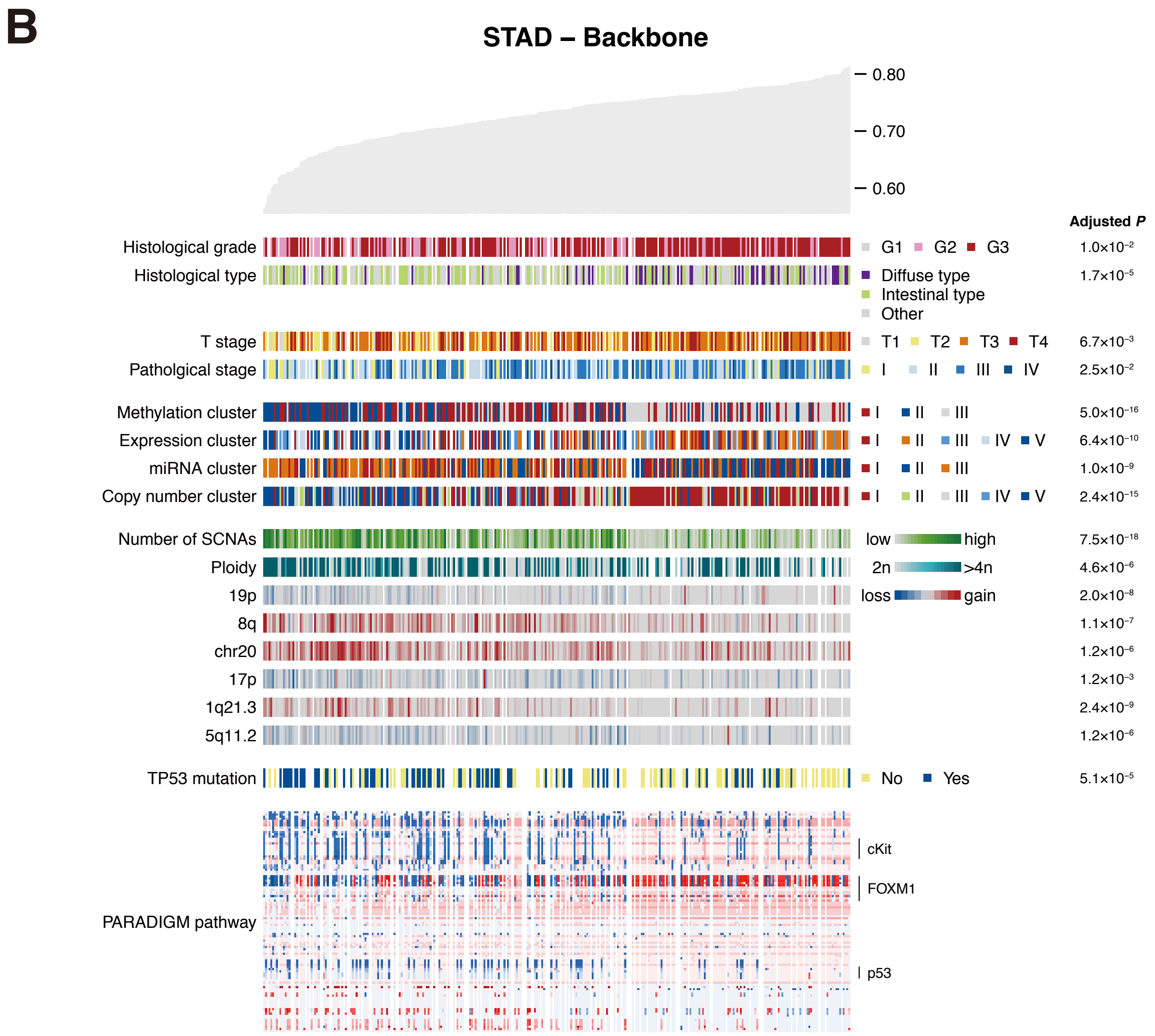

Supplementary Figure S14

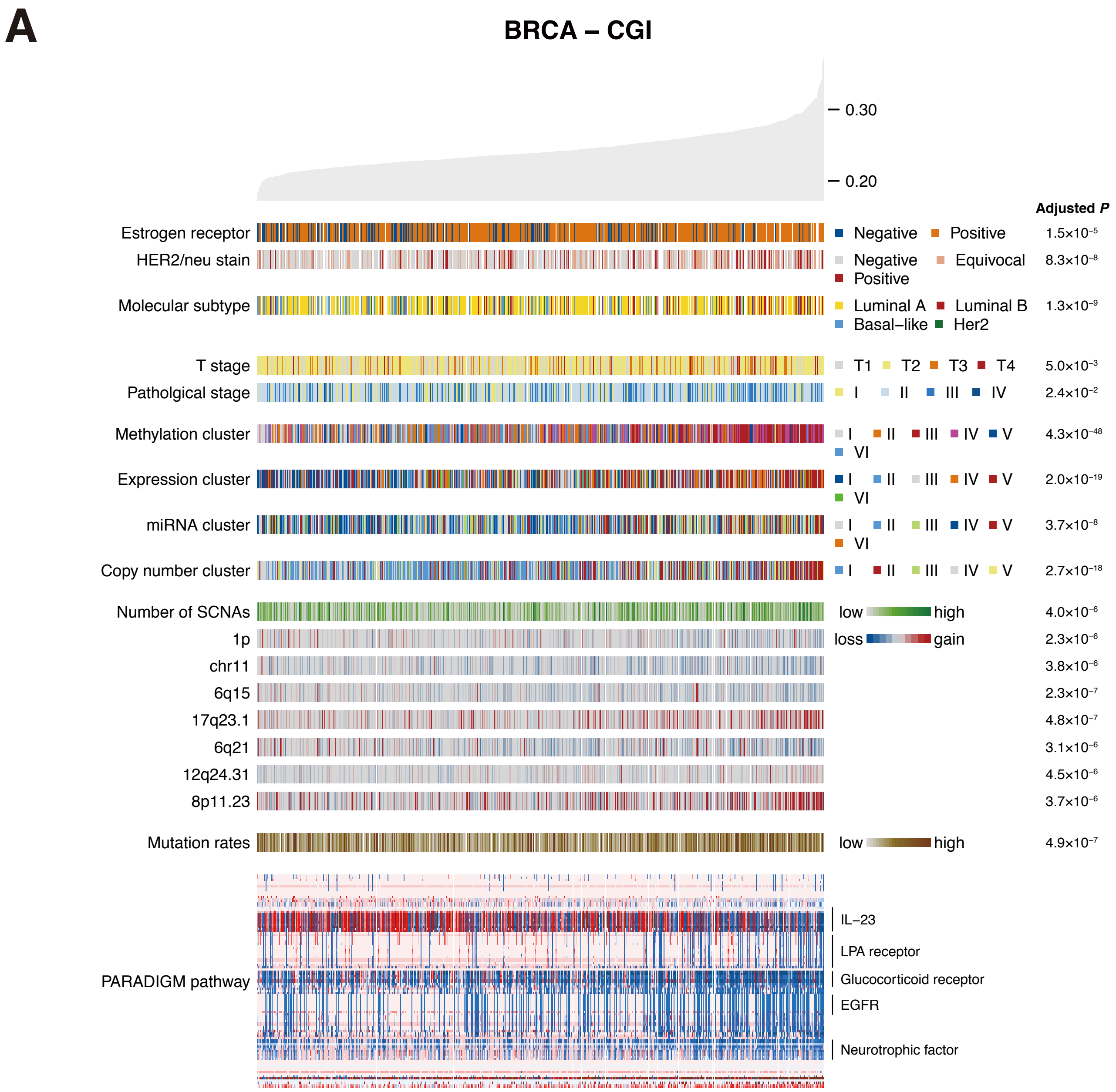

Supplementary Figure S14

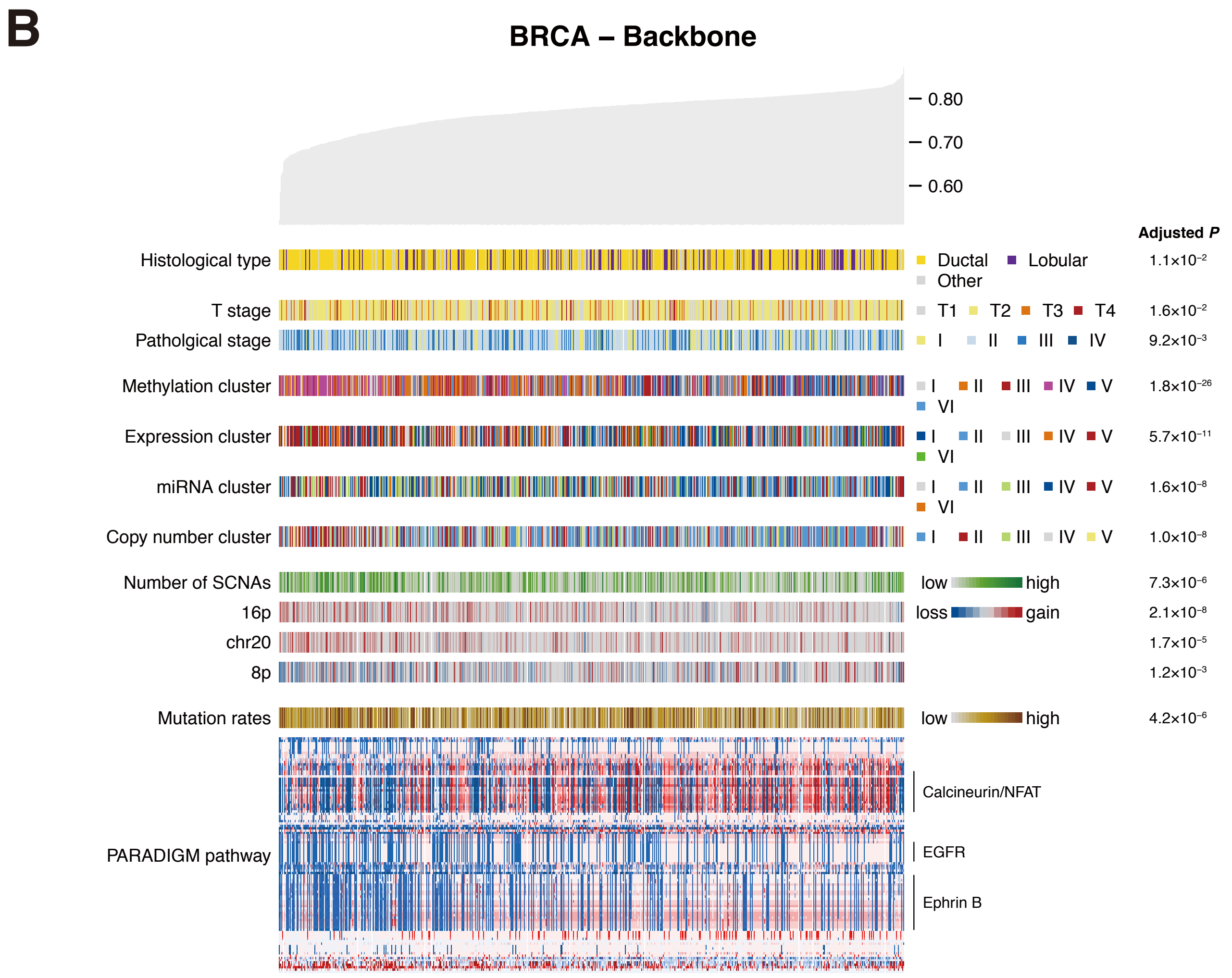

Supplementary Figure S15

A

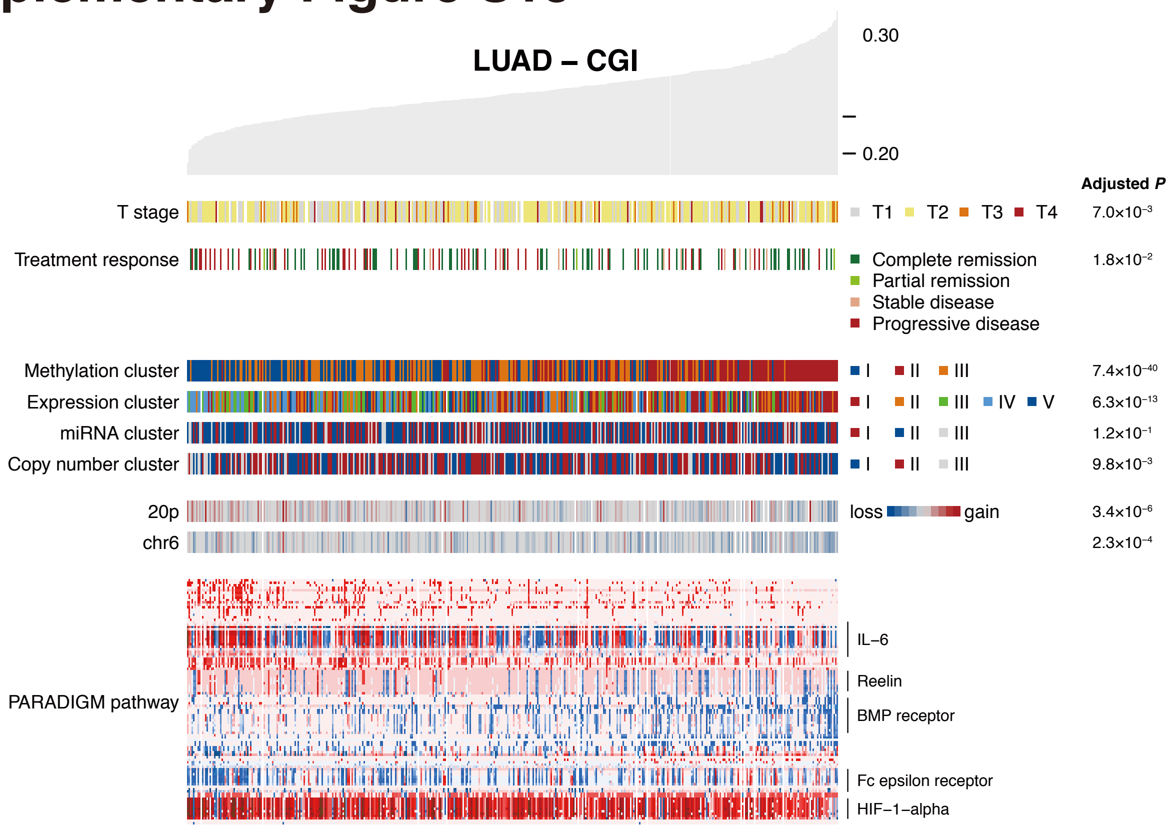

B

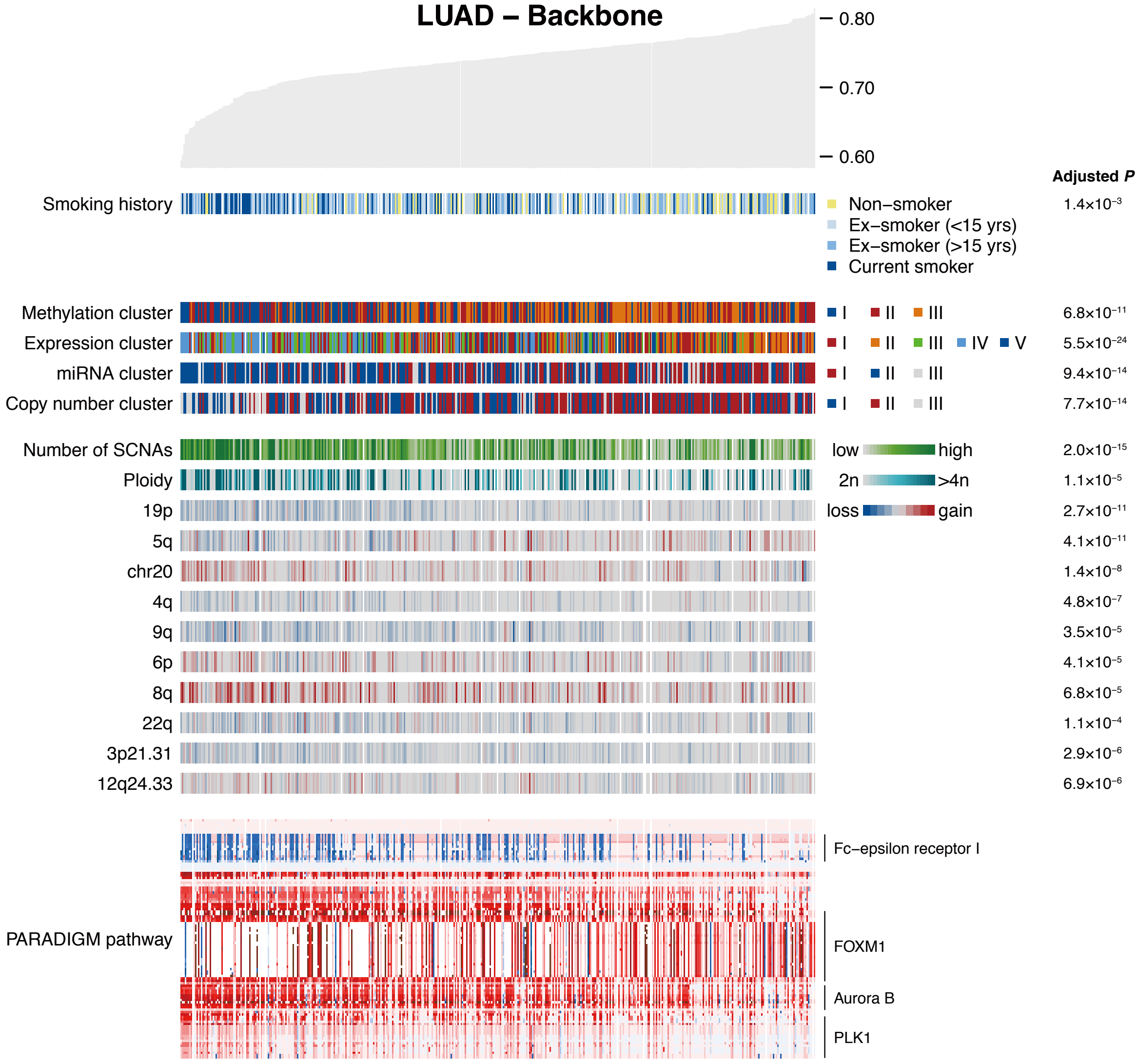

Supplementary Figure S16

A

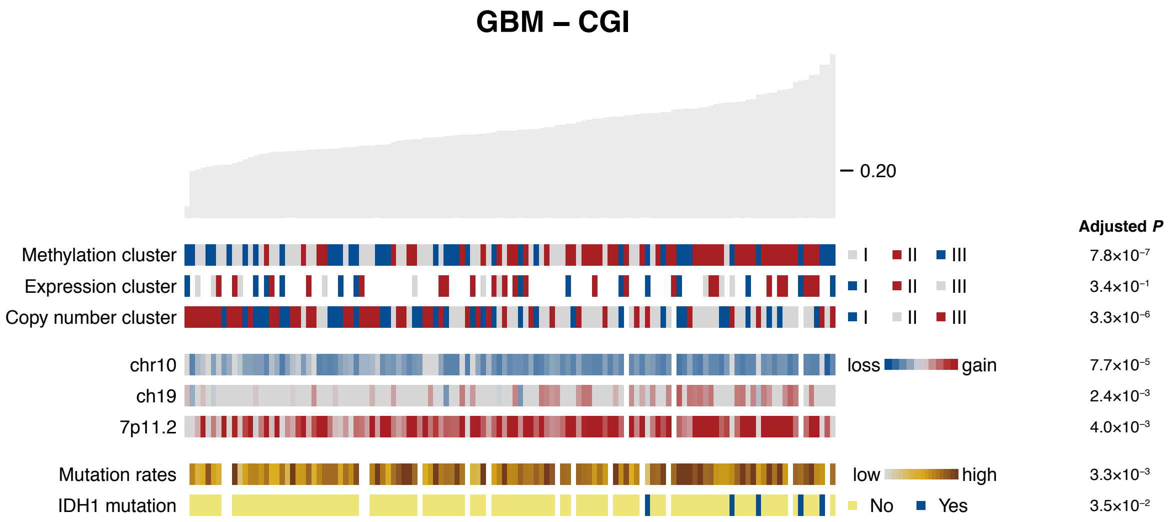

B

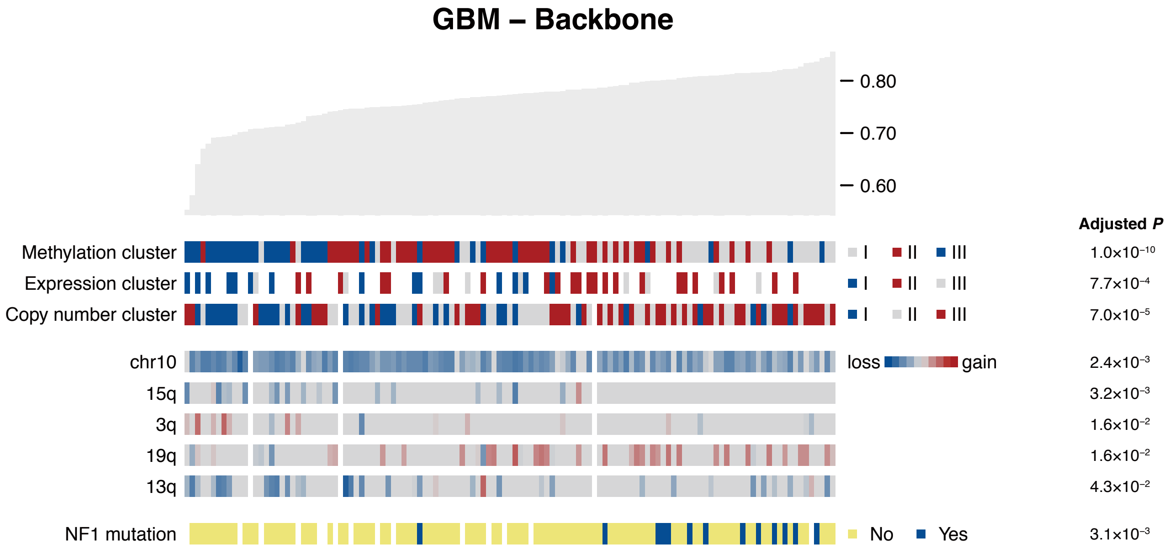

Supplementary Figure S17

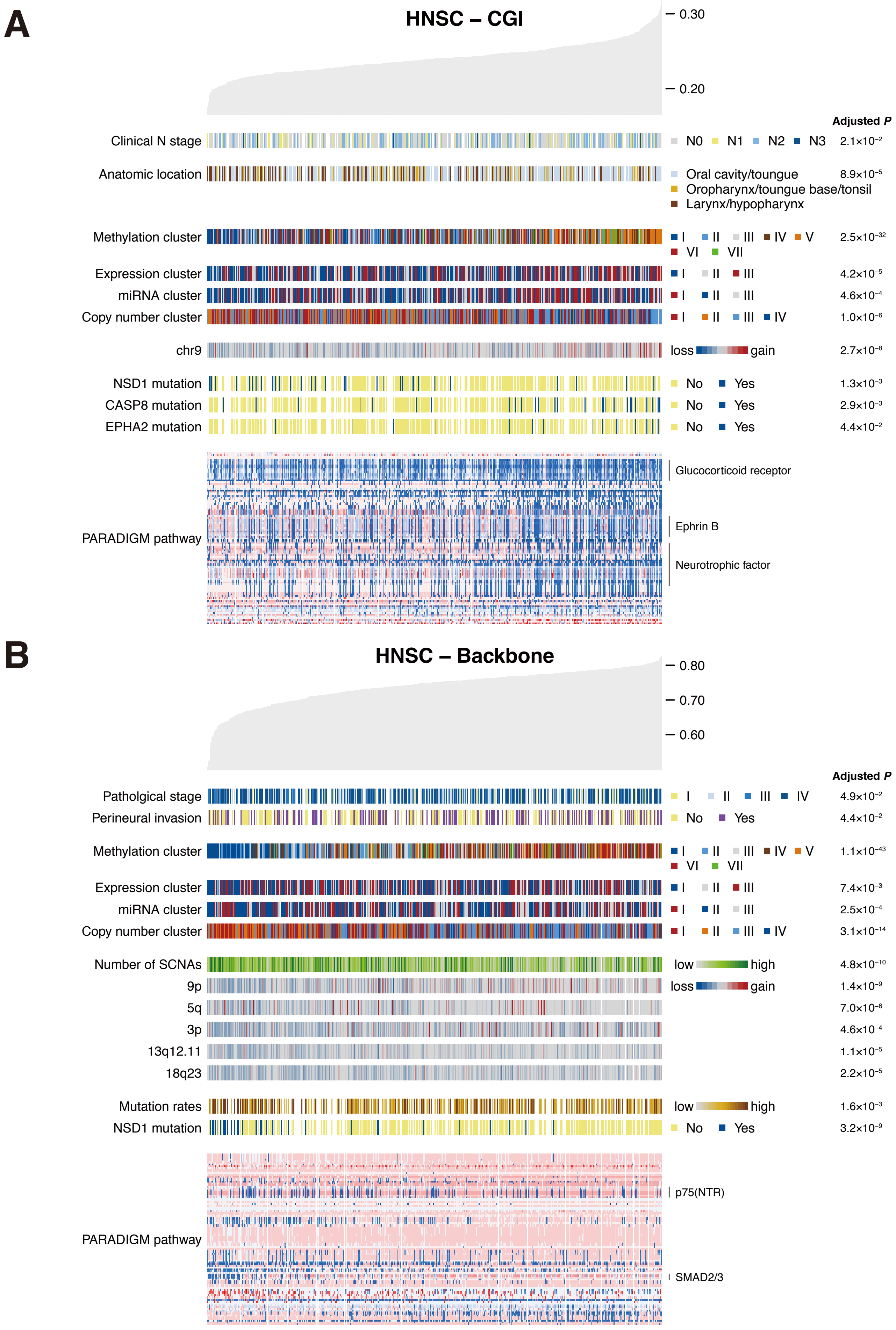

Supplementary Figure S18

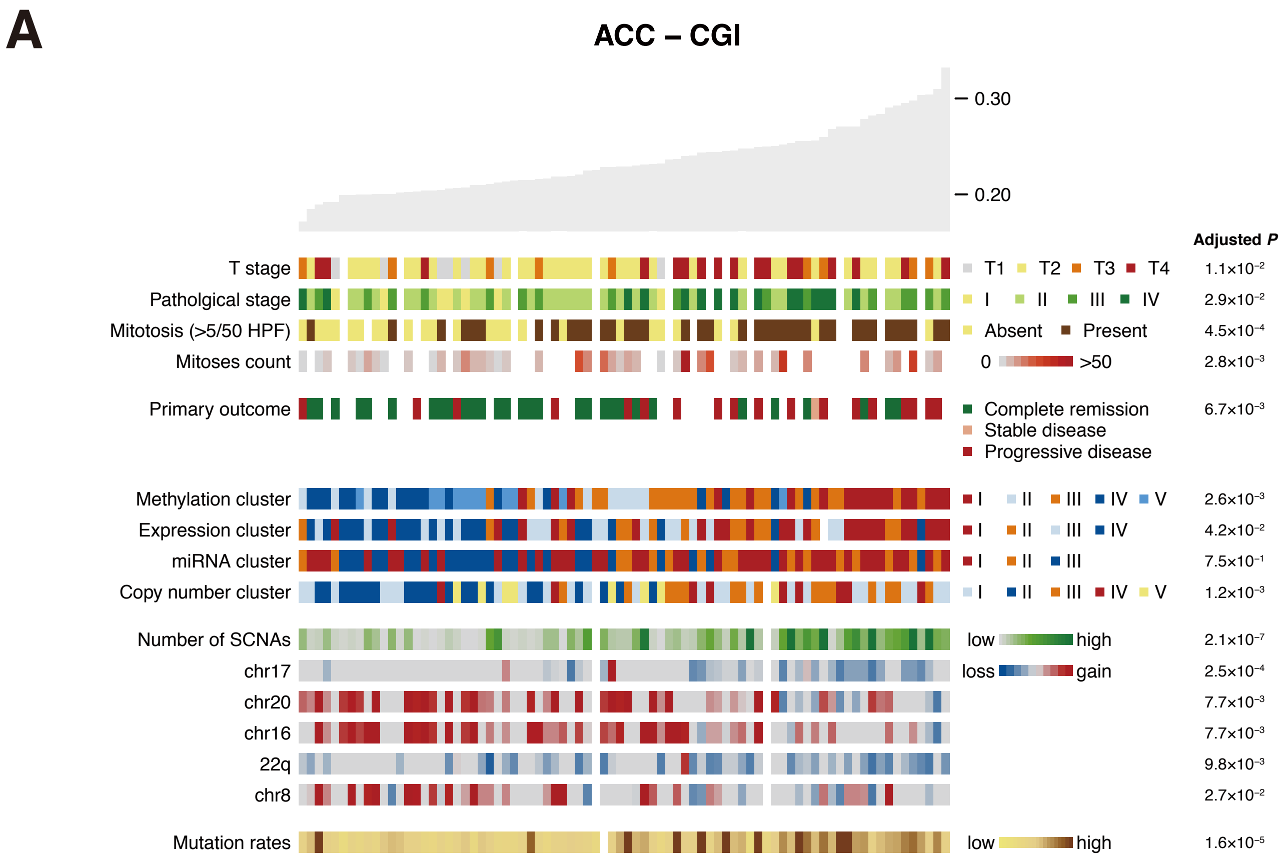

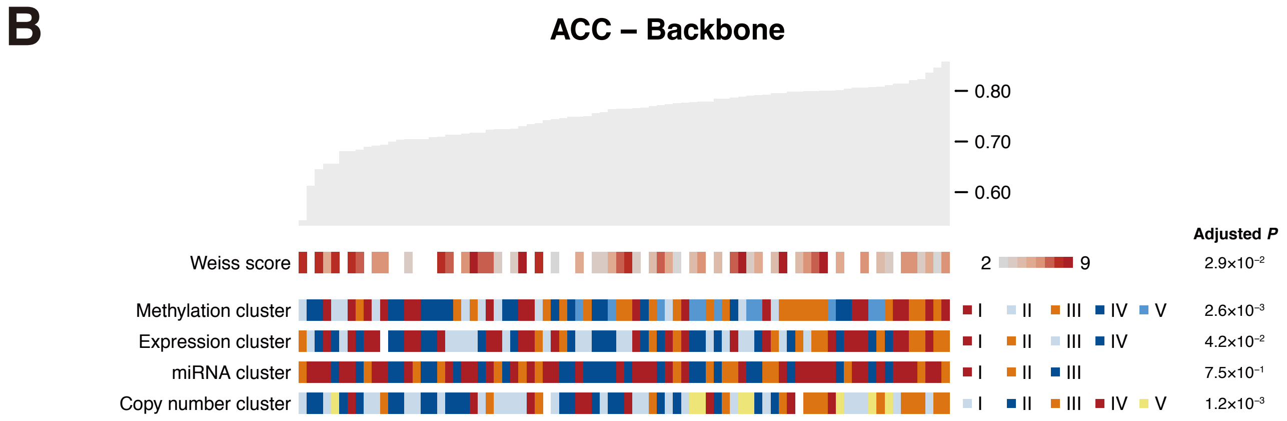

Supplementary Figure S19

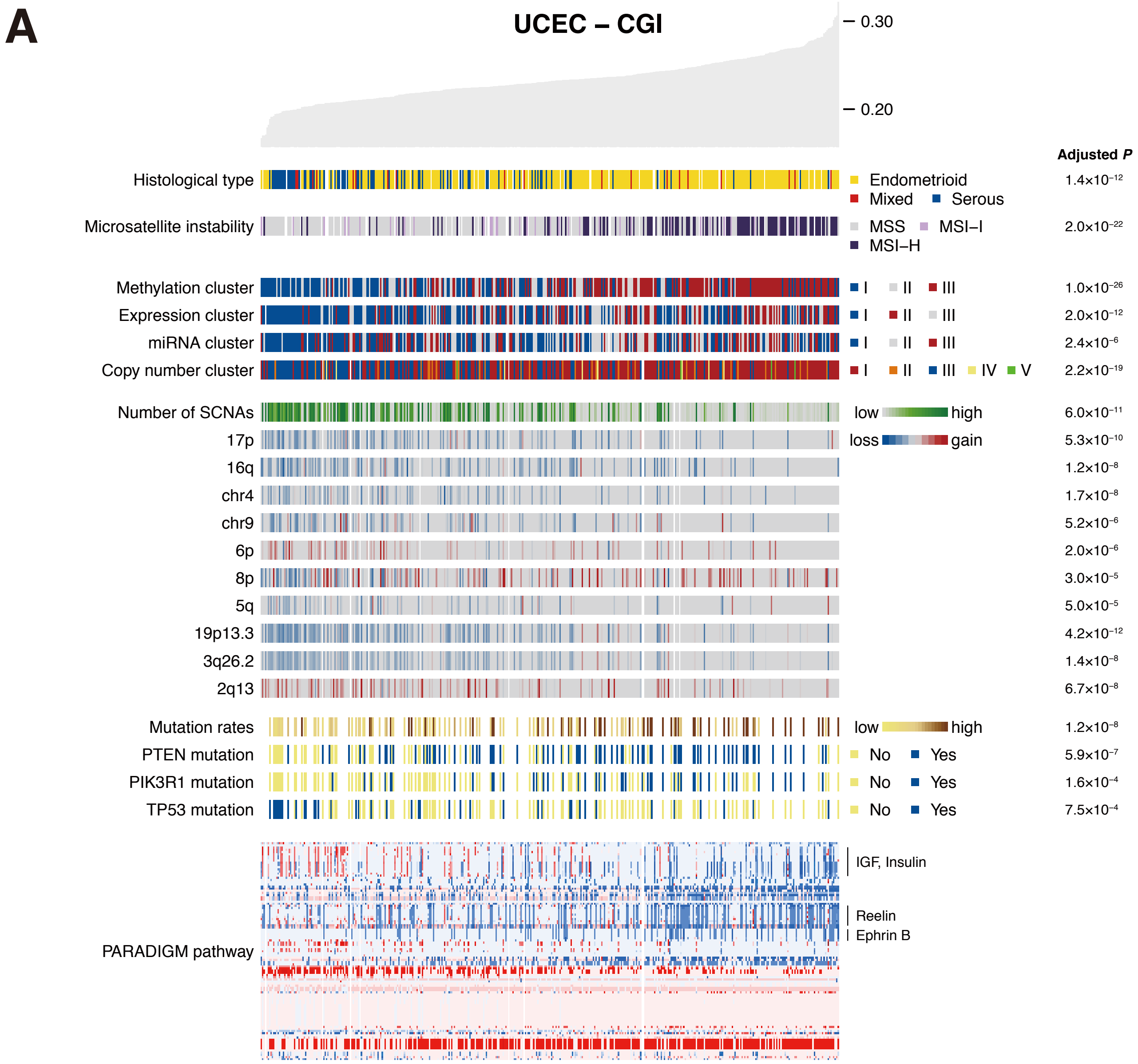

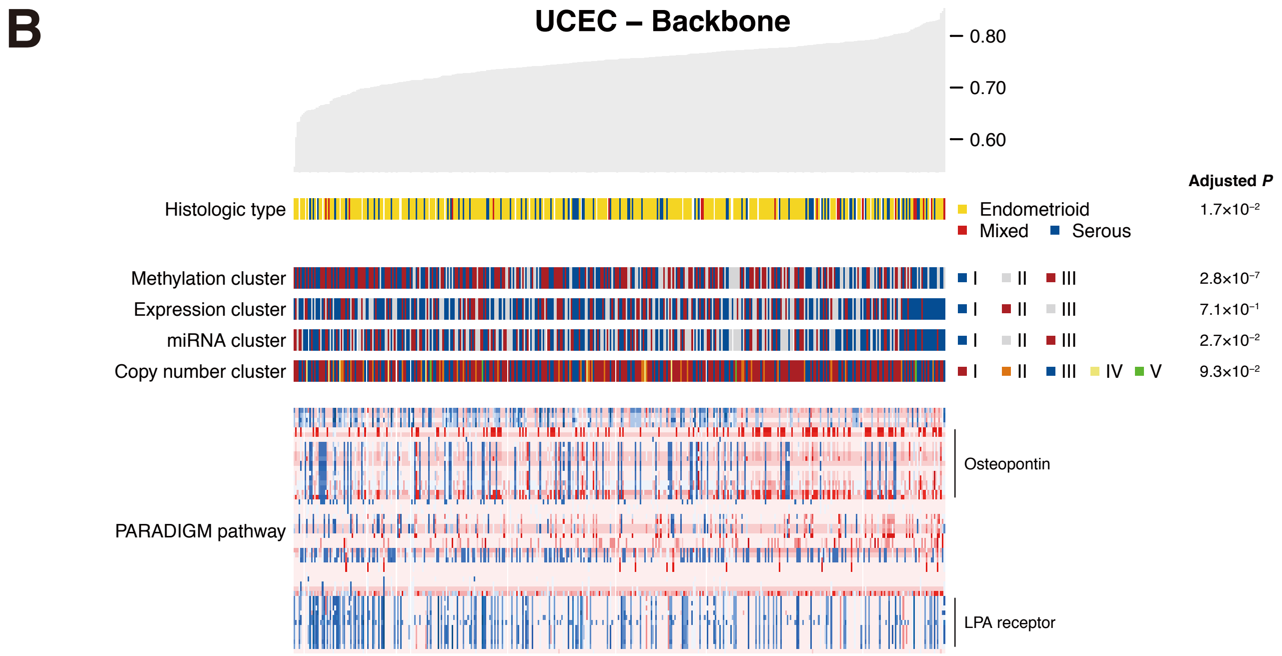

Supplementary Figure S20

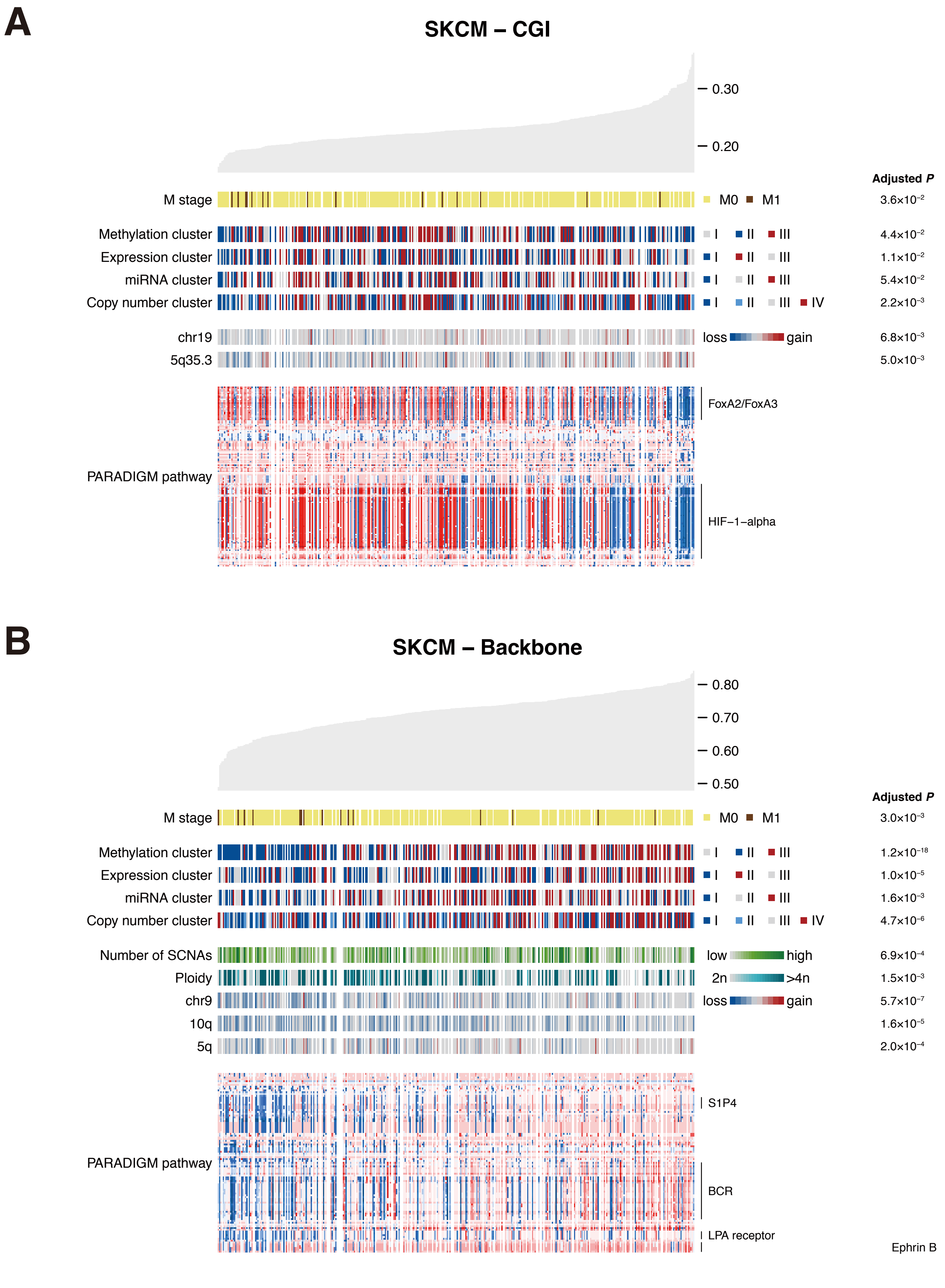

Supplementary Figure S21

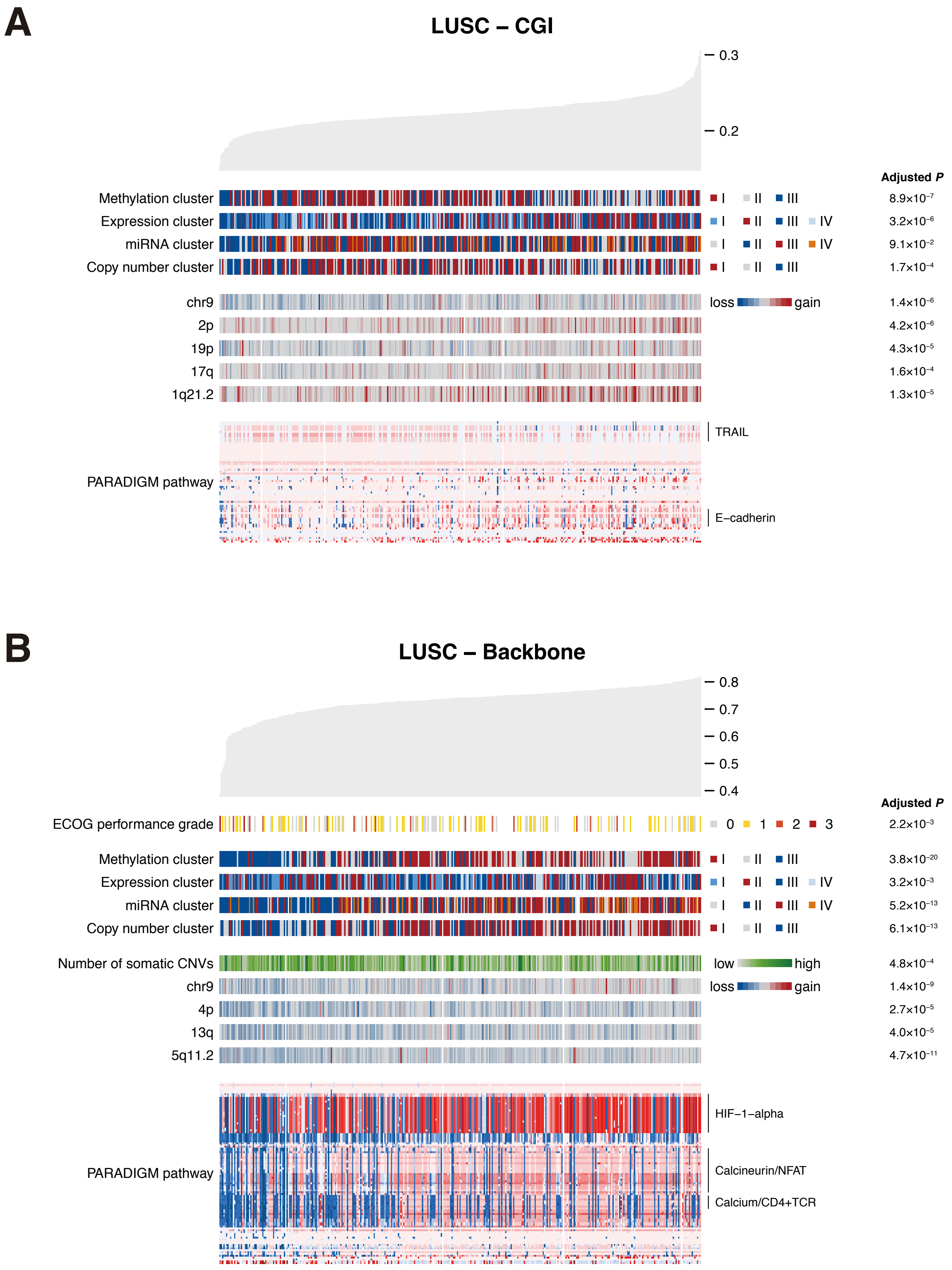

Supplementary Figure S22

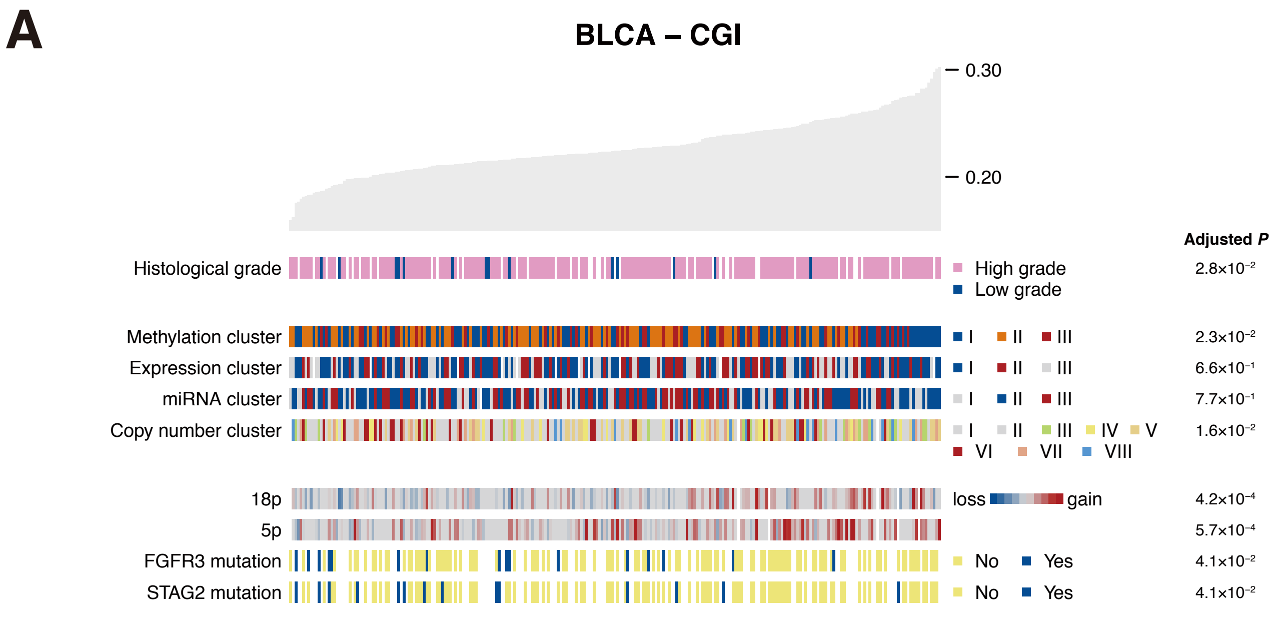

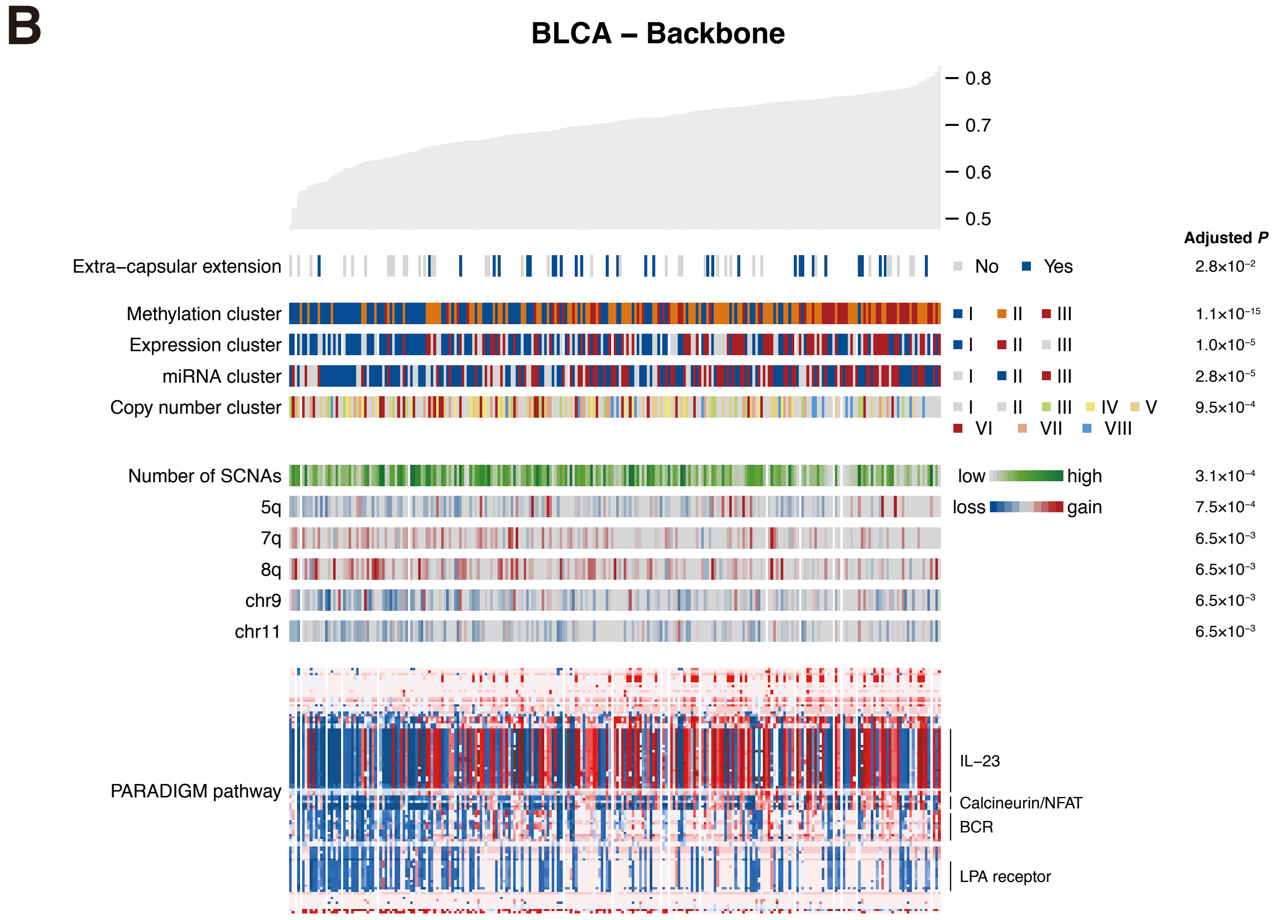

Supplementary Figure S23

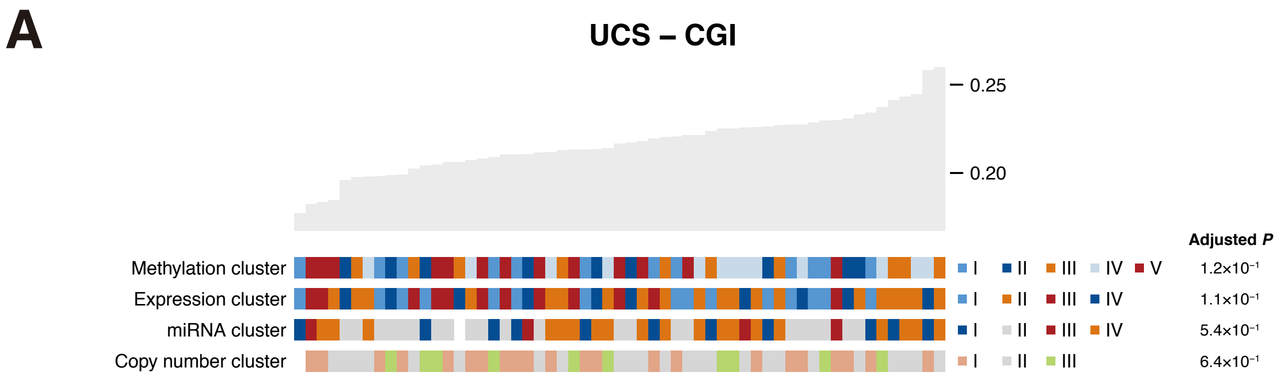

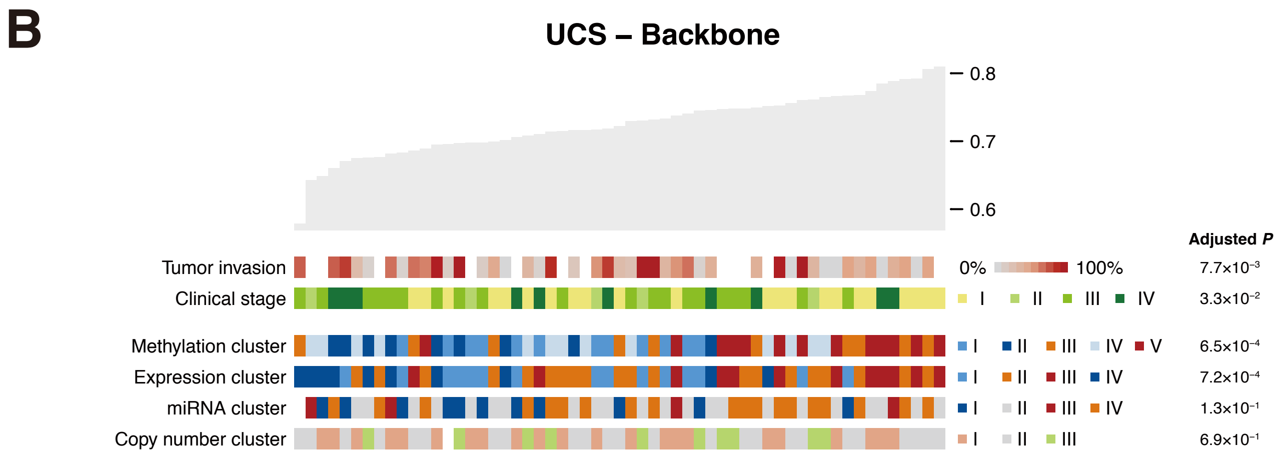

Supplementary Figure S24

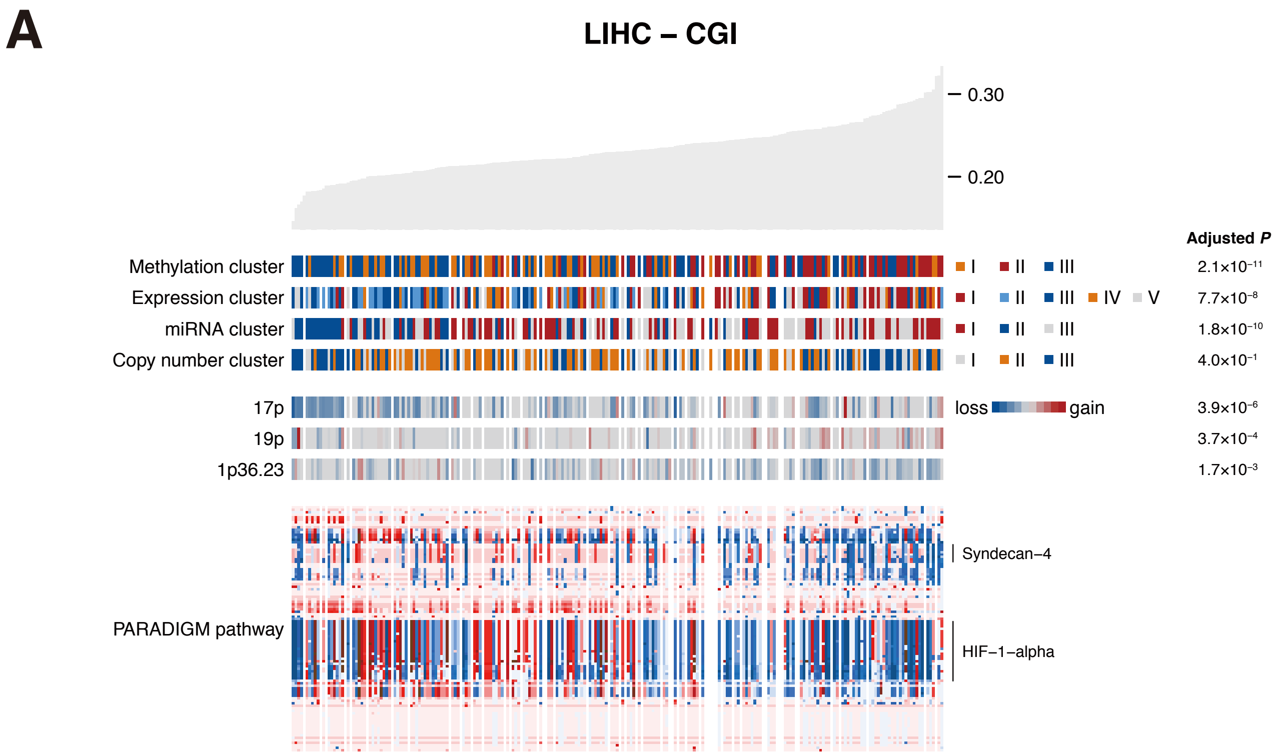

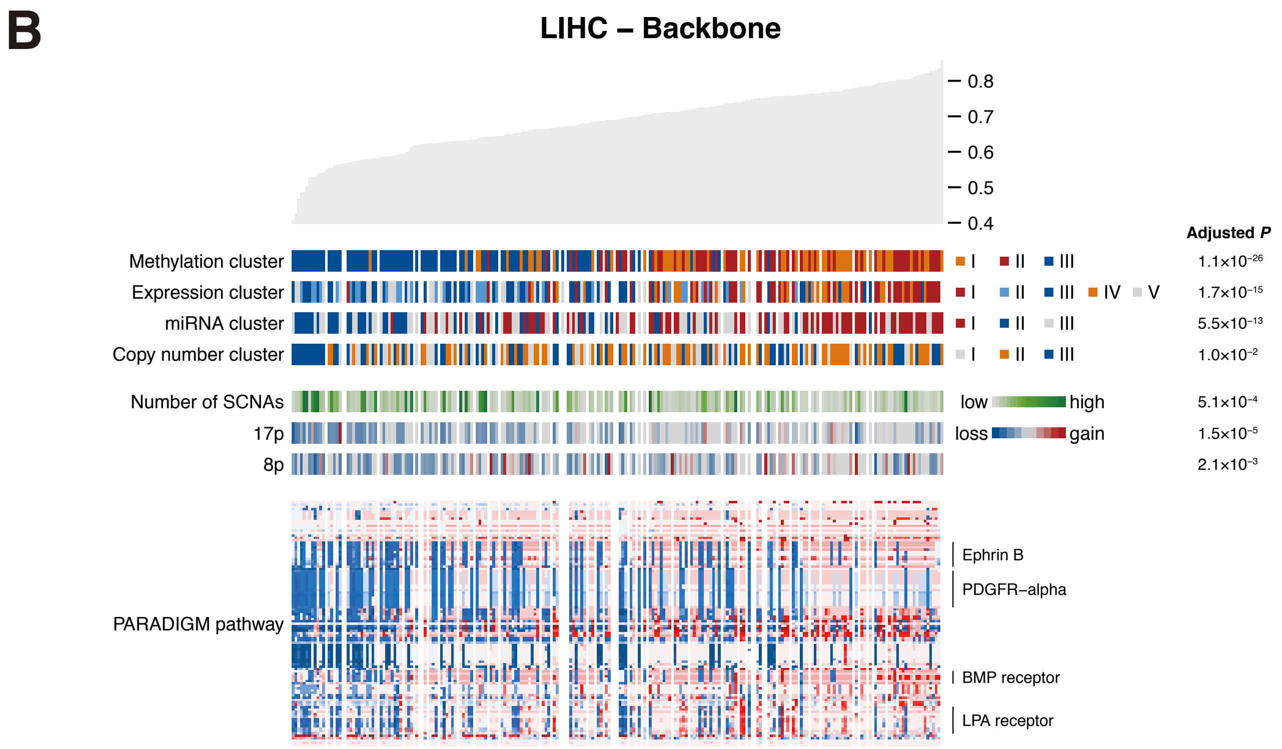

Supplementary Figure S25

A

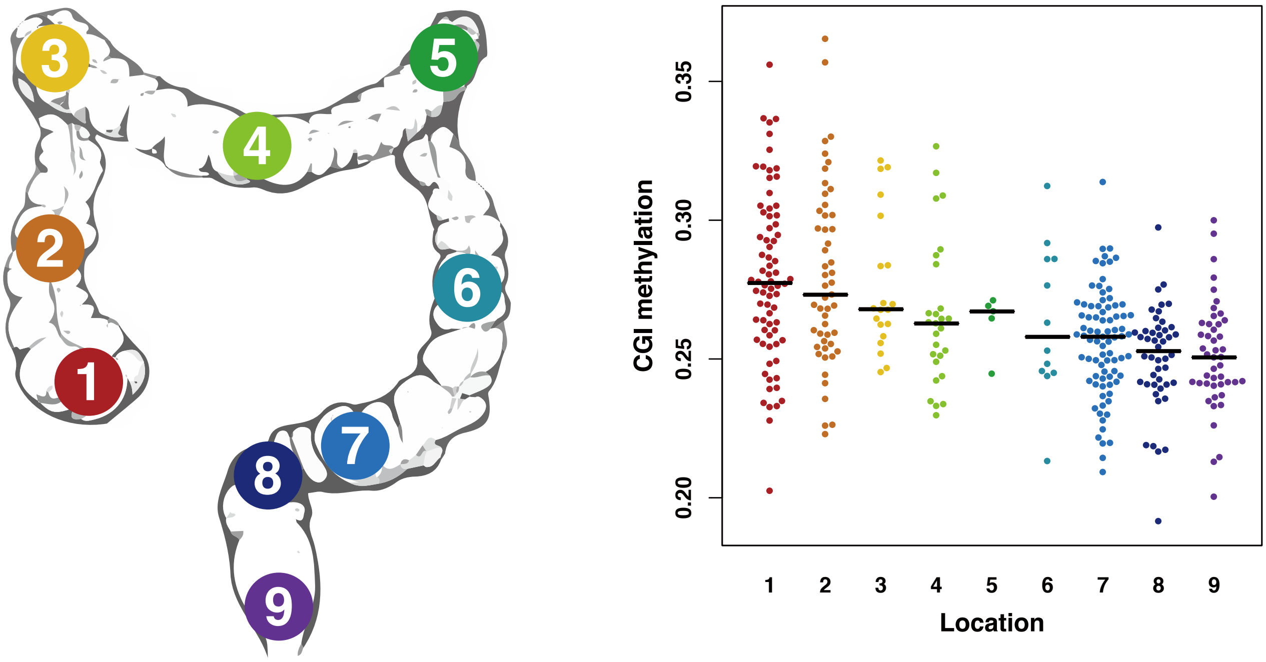

B

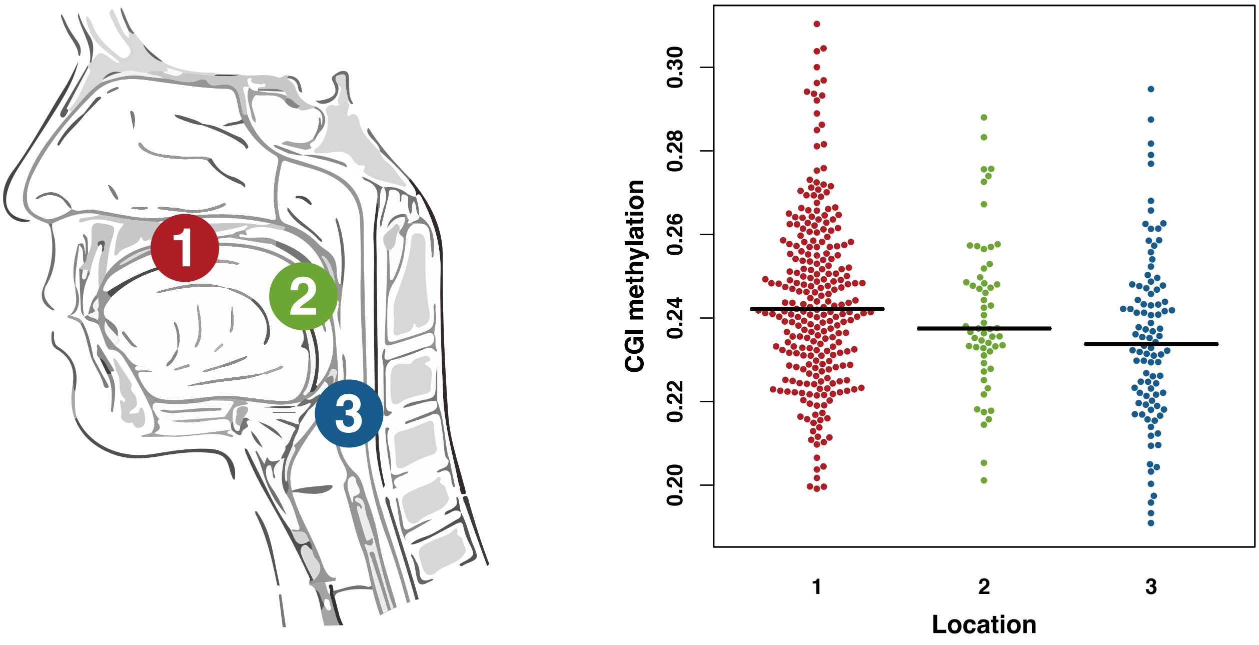

Supplementary Figure S26

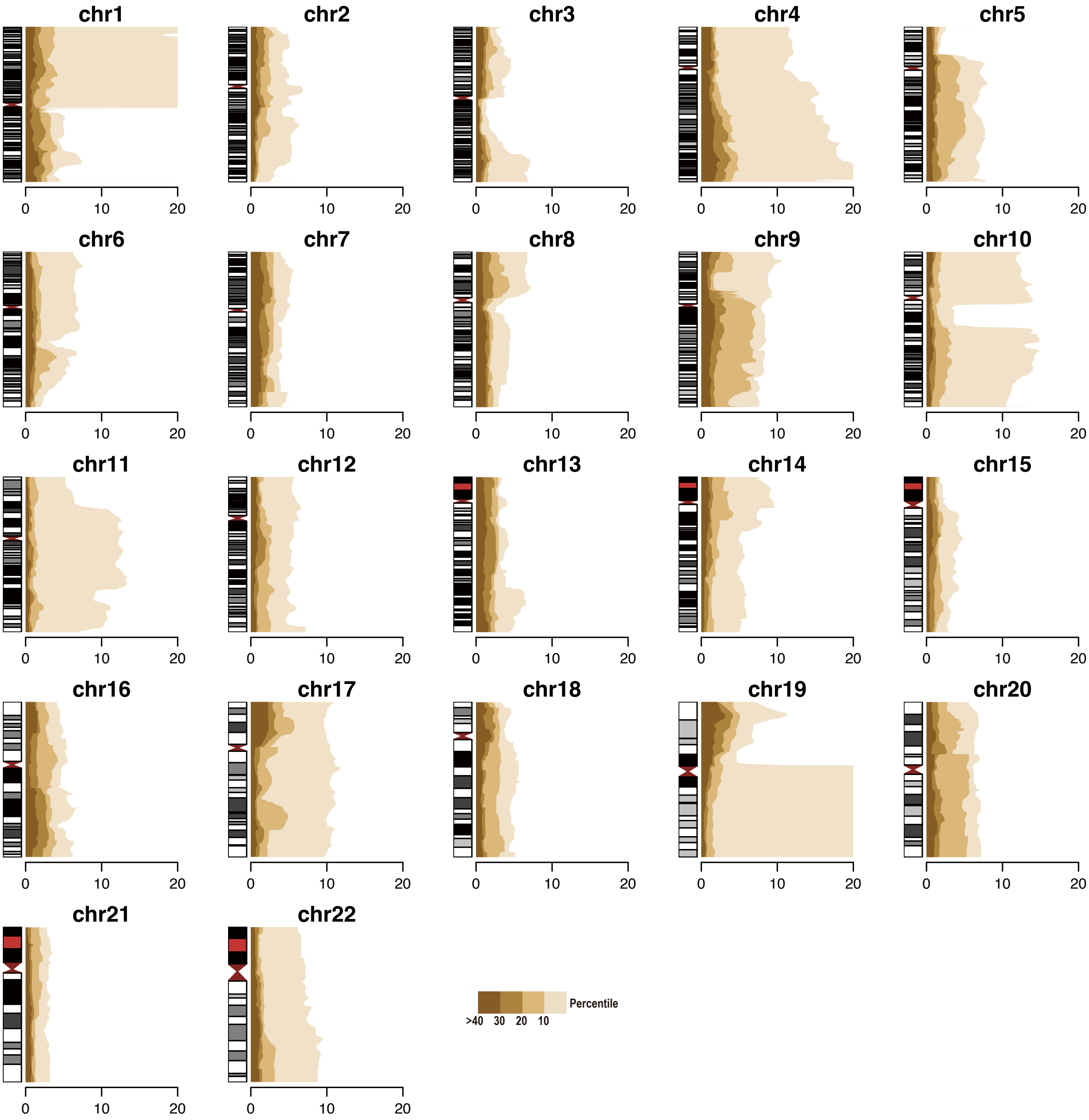

Supplementary Figure S27

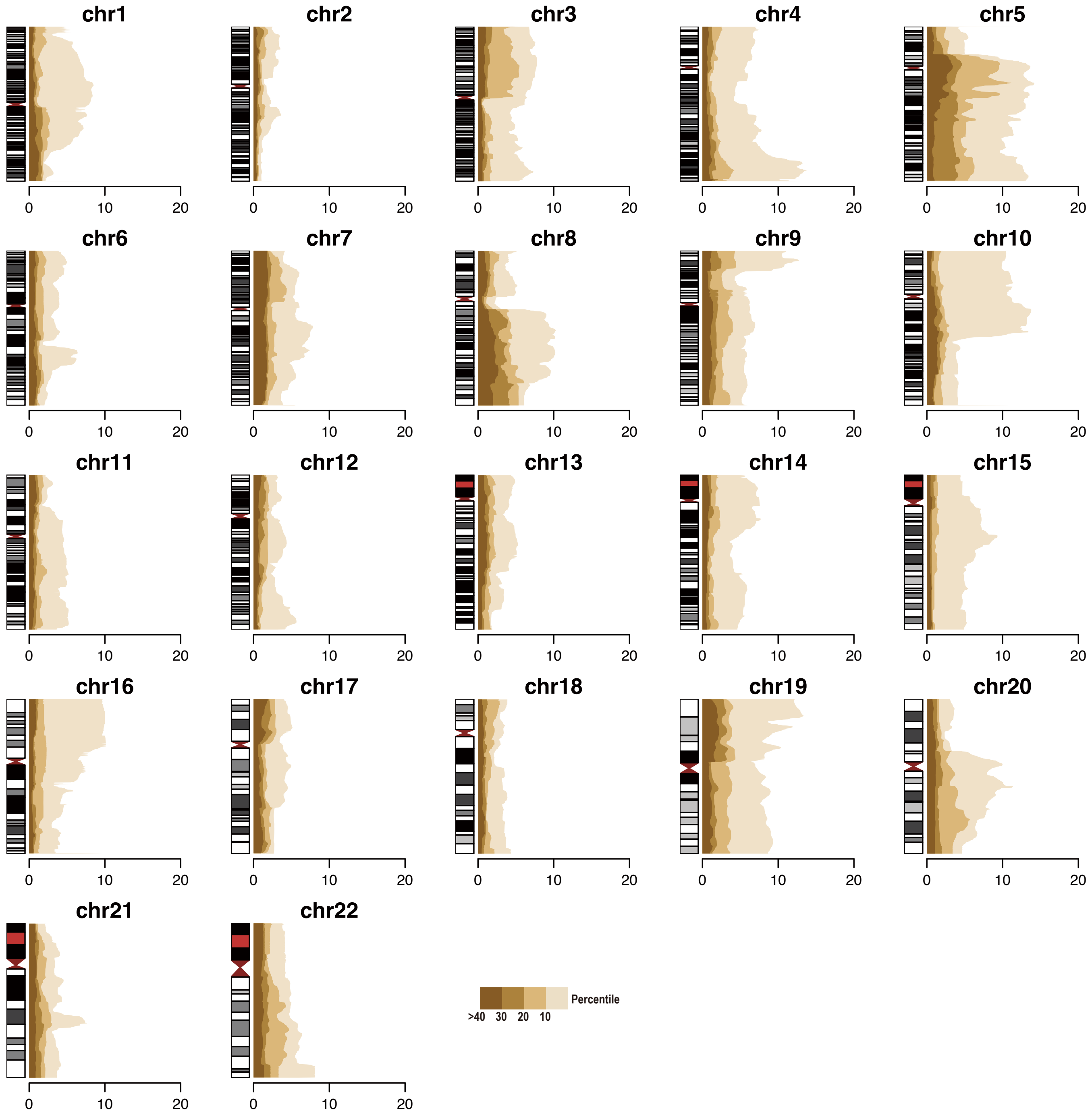

Supplementary Figure S28

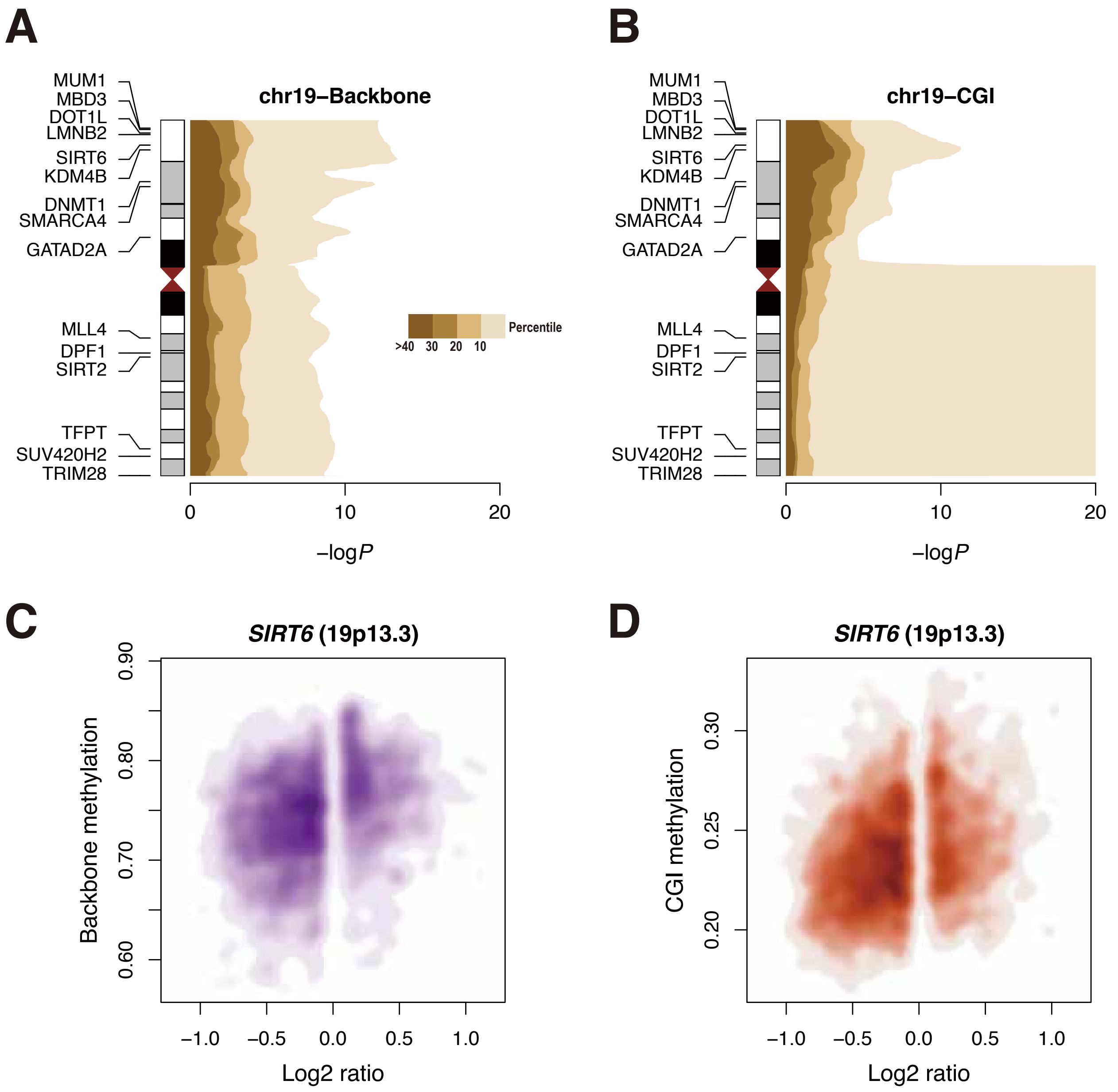

Supplementary Figure S29

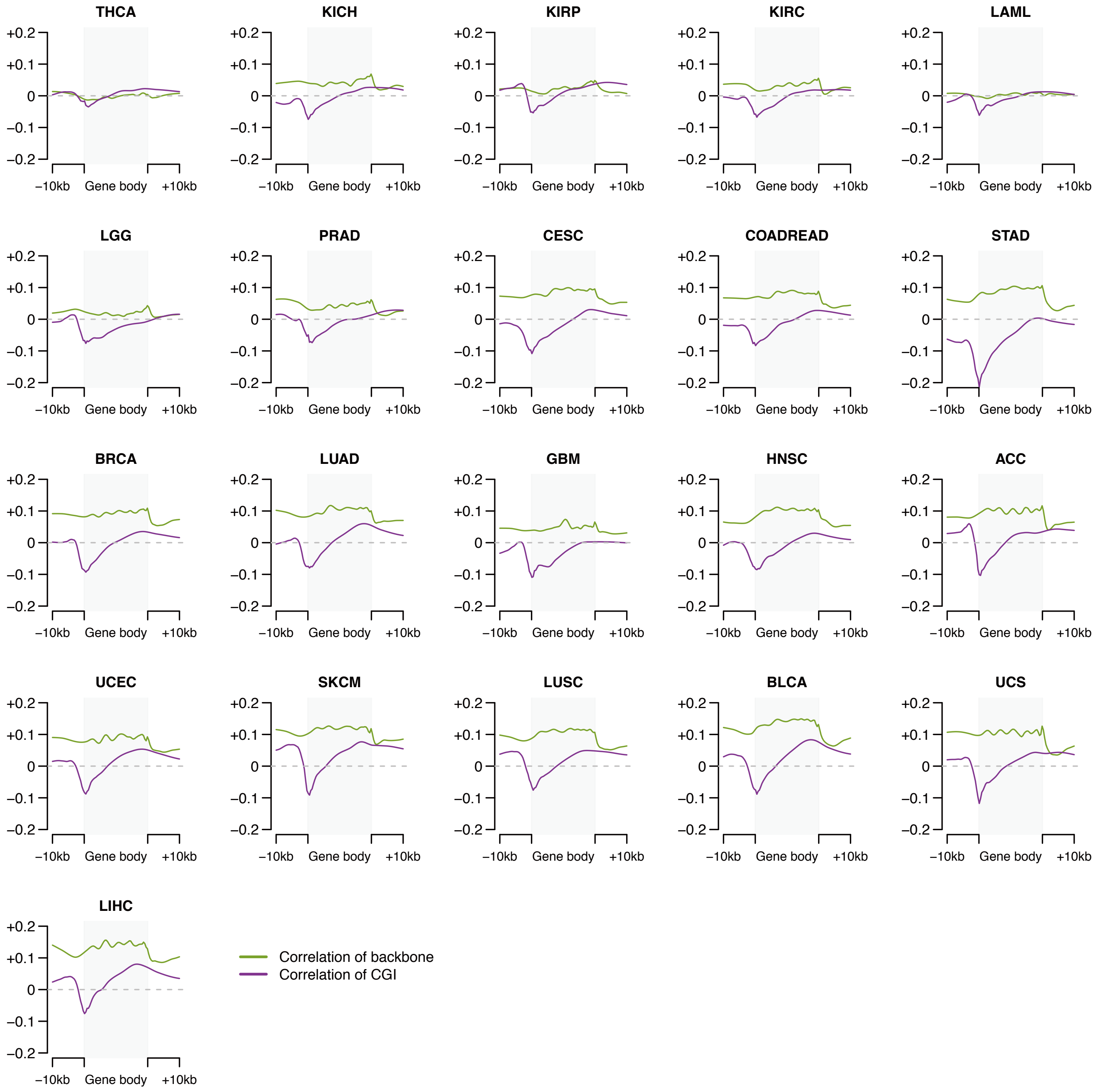

Supplementary Figure S30

A

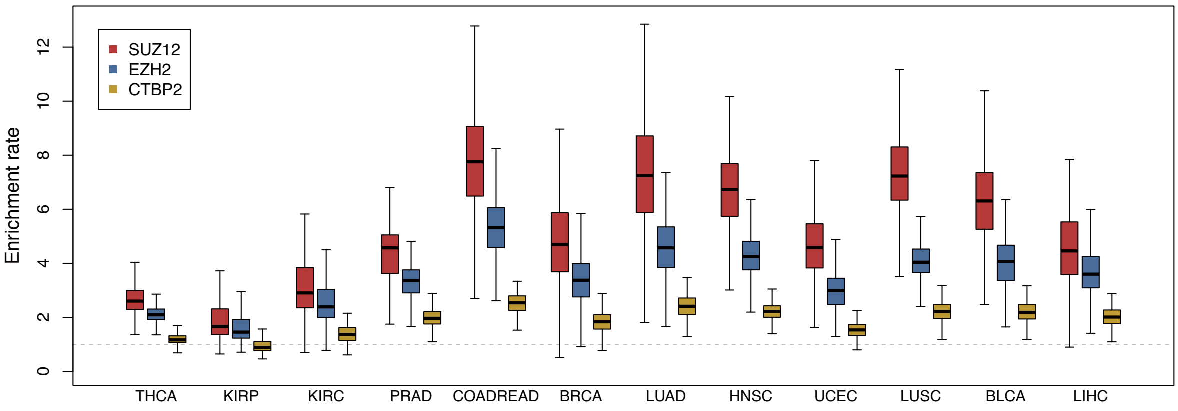

B

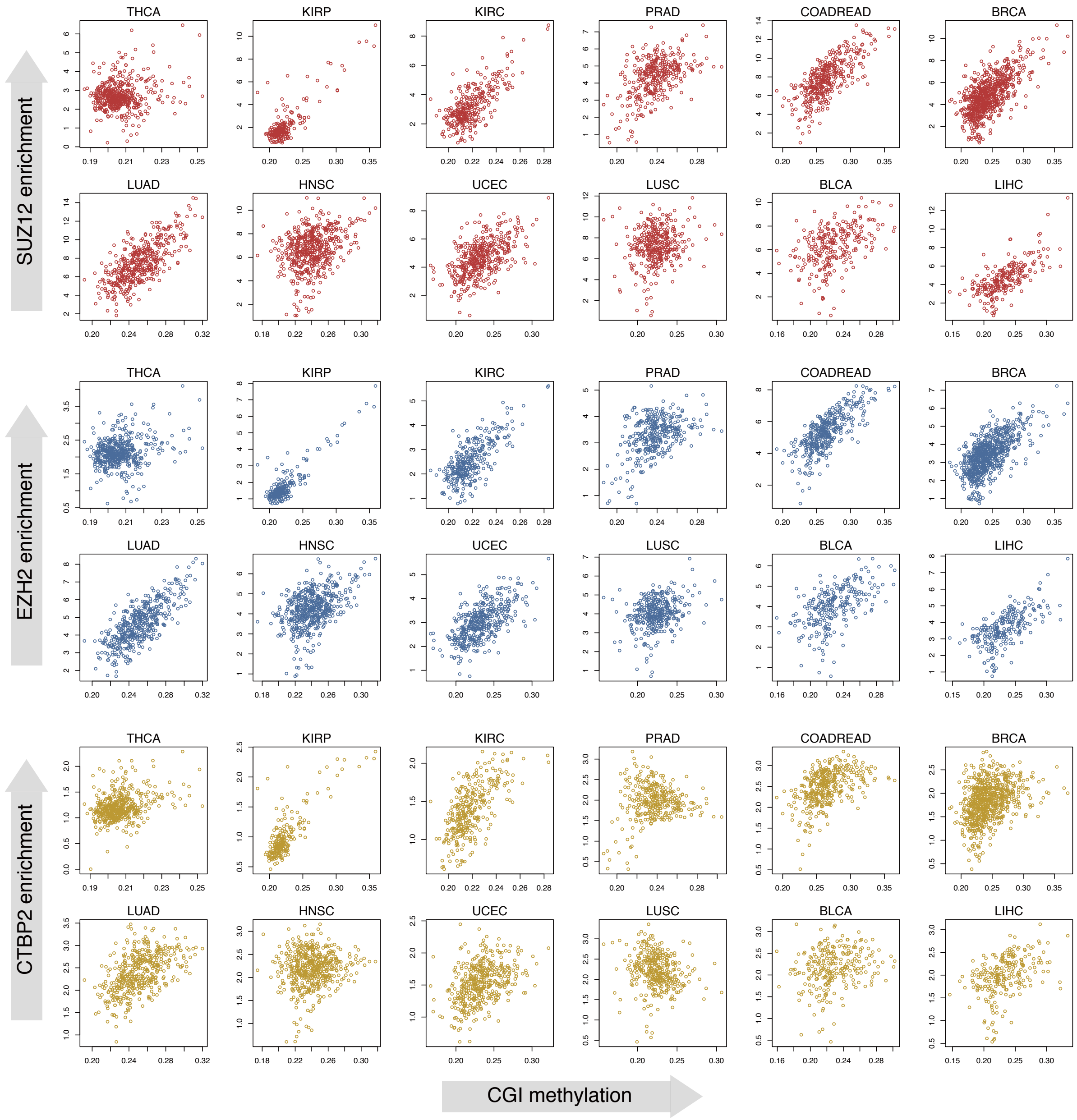

Supplementary Figure S31

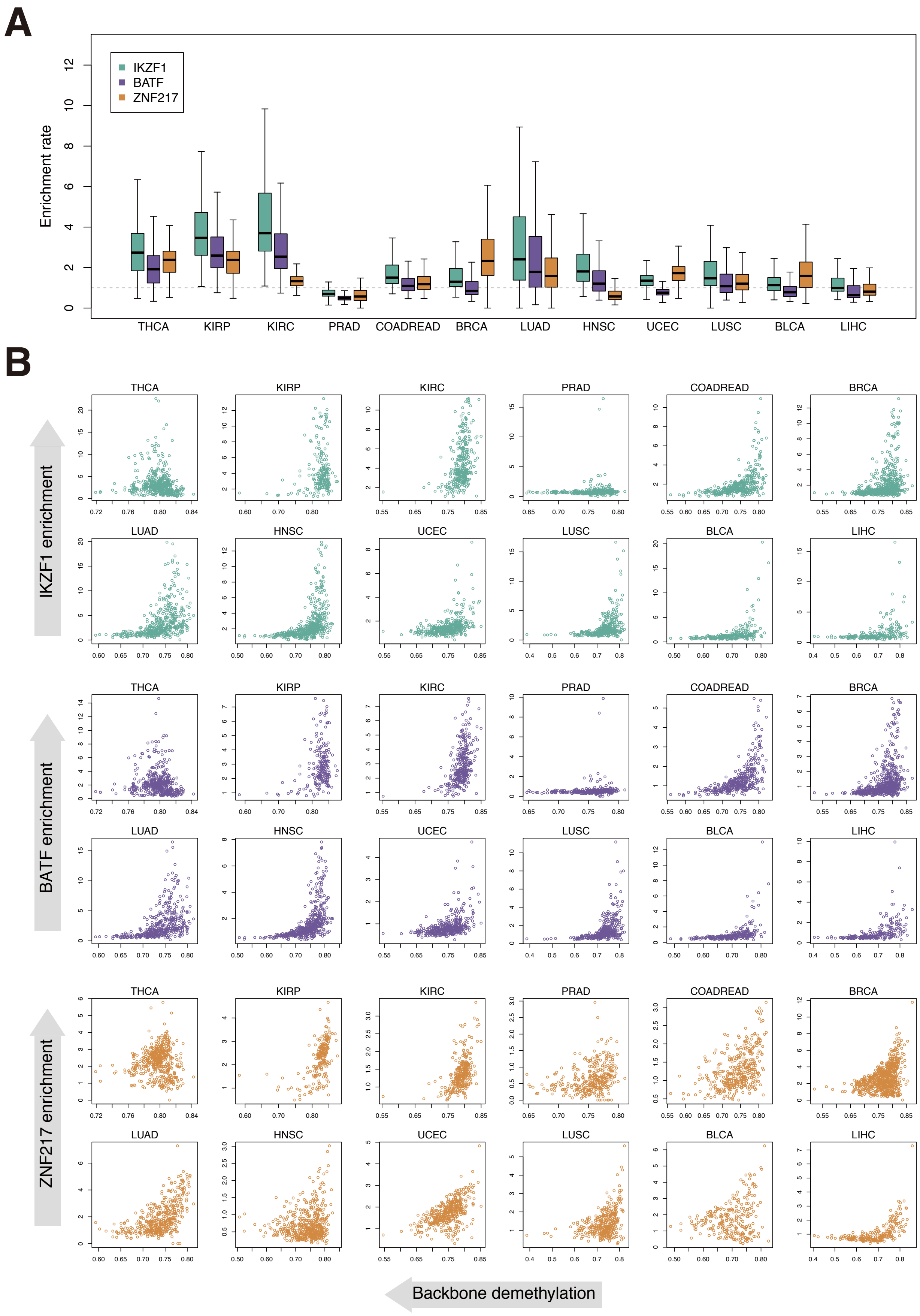

Supplementary Figure S32

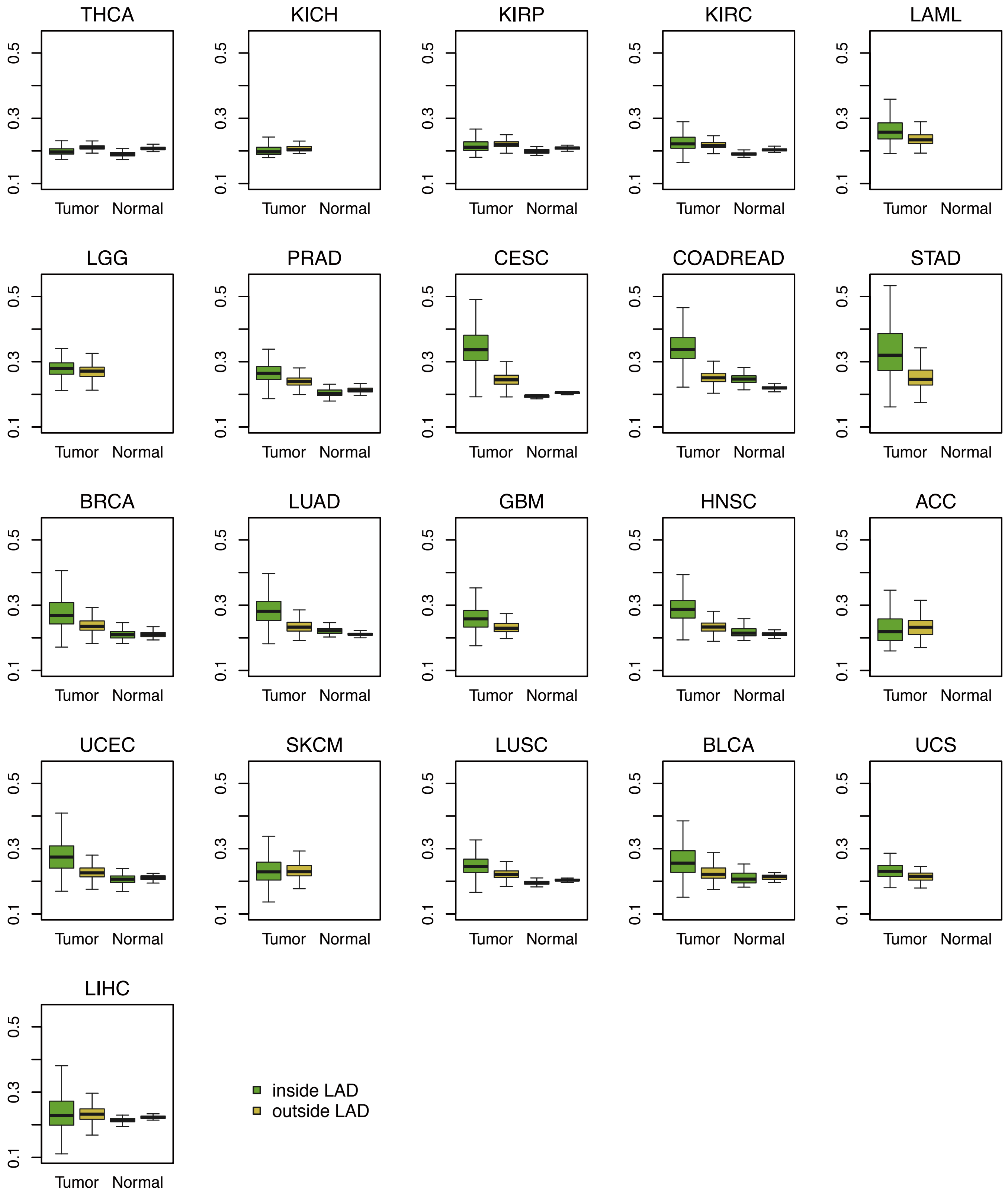

Supplementary Figure S33

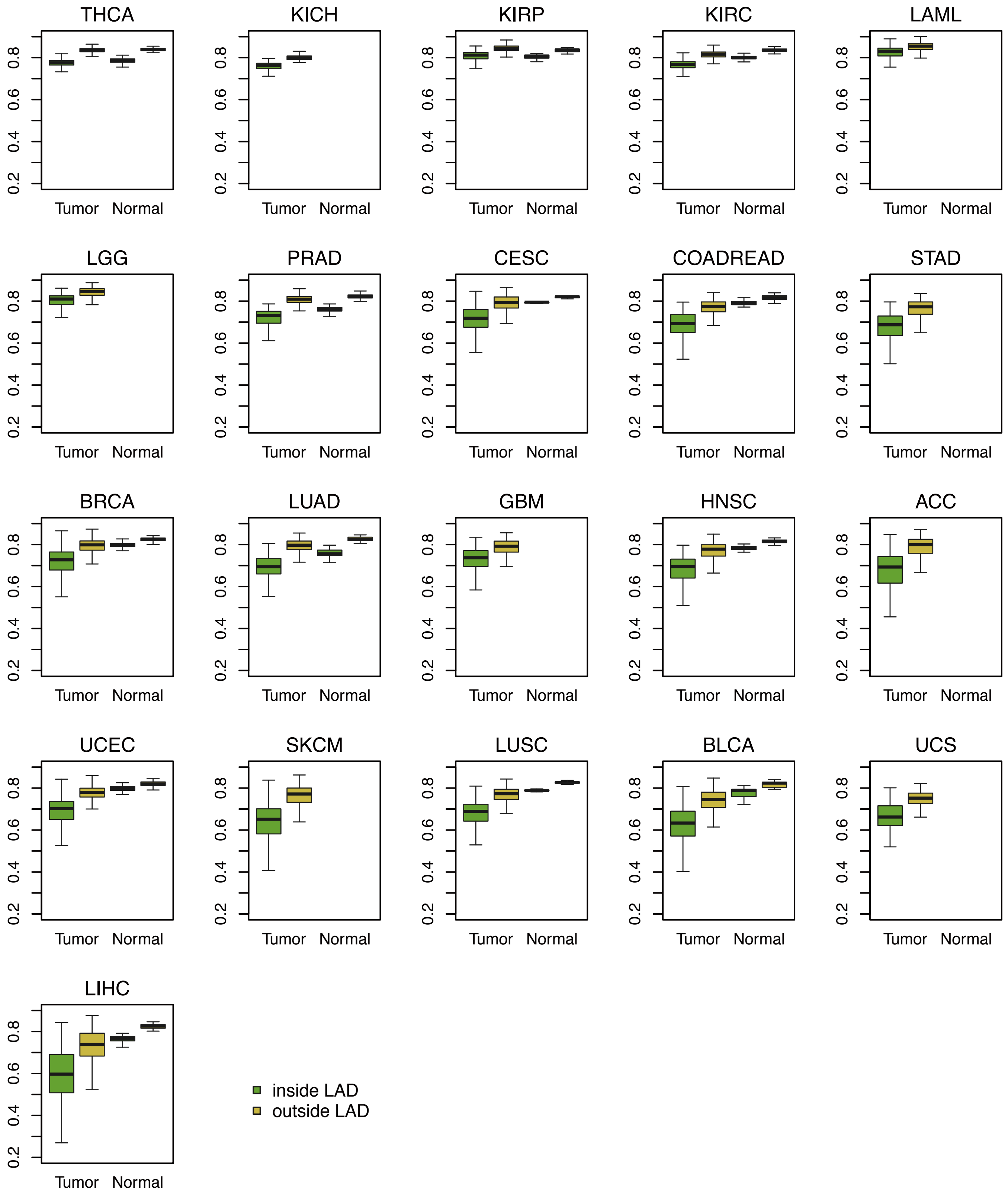

Supplementary Figure S34

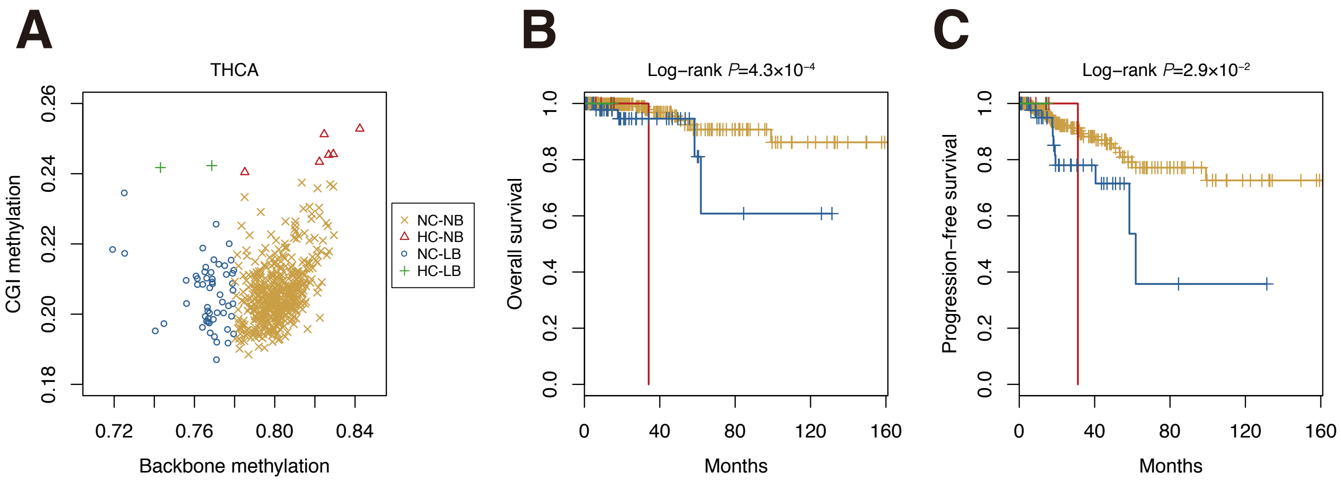

D

Overall survival

	Unadjusted			Model 1			Model 2			Model 3		
**Parameter**	**HR**	**95% CI**	***P***	**HR**	**95% CI**	***P***	**HR**	**95% CI**	***P***	**HR**	**95% CI**	***P***
Methylation status												
NC–NB	1.0			1.0			1.0			1.0		
HC–NB	24.0	2.8–208.5	0.004	3.1	0.3–30.4	0.331	9.3	0.9–101.0	0.067	0.0	0.0– Inf	0.998
NC–LB	3.2	1.0– 10.3	0.057	2.0	0.6– 6.9	0.289	1.3	0.3– 6.1	0.718	1.4	0.3– 6.6	0.662
Age												
Age (yrs)				1.10	1.05–1.14	<0.001						
Gender												
Female				1.0			1.0			1.0		
Male				1.1	0.3– 4.1	0.896	2.1	0.6– 7.6	0.253	2.3	0.6– 8.8	0.231
Pathological stage												
Stage I/II							1.0			1.0		
Stage III							5.4	1.3– 22.5	0.022	5.3	1.2–22.4	0.024
Stage IV							18.3	3.3–100.2	0.001	15.8	3.0–83.7	0.001
BRAF mutation												
Absent										1.0		
Present										0.7	0.2– 2.5	0.574

E

Progression-free survival

	Unadjusted			Model 1			Model 2			Model 3		
**Parameter**	**HR**	**95% CI**	***P***	**HR**	**95% CI**	***P***	**HR**	**95% CI**	***P***	**HR**	**95% CI**	***P***
Methylation status												
NC–NB	1.0			1.0			1.0			1.0		
HC–NB	5.3	0.7–39.8	0.104	2.4	0.3–19.0	0.402	3.6	0.5–28.5	0.227	0.0	0.0–Inf	0.997
NC–LB	2.5	1.2– 5.1	0.014	2.3	1.1– 4.8	0.025	1.9	0.9– 4.4	0.112	1.5	0.6–3.7	0.335
Age												
Age (yrs)				1.03	1.01–1.05	0.004						
Gender												
Female				1.0			1.0			1.0		
Male				1.4	0.7– 2.8	0.310	1.4	0.7– 2.8	0.348	1.7	0.8–3.5	0.193
Pathological stage												
Stage I/II							1.0			1.0		
Stage III							2.4	1.1– 5.0	0.022	3.0	1.4–6.6	0.006
Stage IV							3.7	1.6– 8.8	0.003	3.6	1.4–9.3	0.007
BRAF mutation												
Absent										1.0		
Present										1.5	0.7–3.4	0.311

Supplementary Figure S35

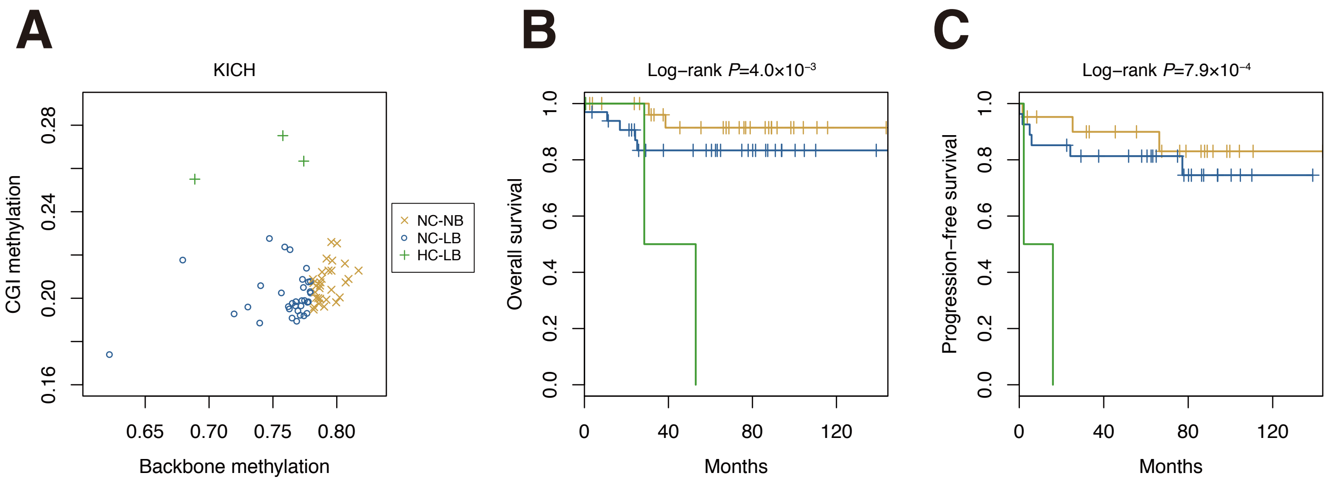

D

Overall survival

	Unadjusted (n=66)			Model 1 (n=66)			Model 2 (n=66)			Model 3 (n=52)		
**Parameter**	**HR**	**95% CI**	***P***	**HR**	**95% CI**	***P***	**HR**	**95% CI**	***P***	**HR**	**95% CI**	***P***
Methylation status												
NC–NB	1.0			1.0			1.0			1.0		
NC–LB	2.6	0.5– 13.2	0.263	4.1	0.6– 26.5	0.142	6.3	0.9– 43.6	0.062	6.8	0.7– 66.9	0.102
HC–LB	14.8	2.1–105.9	0.007	11.1	1.2–102.5	0.035	20.1	1.8–229.6	0.016	45.8	2.1–982.6	0.015
Age												
Age (yrs)				1.07	0.99–1.14	0.078	1.06	0.99–1.14	0.096	1.06	0.96–1.16	0.262
Gender												
Female				1.0			1.0			1.0		
Male				0.4	0.1– 2.8	0.387	0.2	0.0– 1.6	0.124	0.1	0.0– 1.6	0.105
Pathological stage												
I/II							1.0			1.0		
III/IV							8.1	1.6– 42.3	0.013	5.9	1.0– 34.3	0.047
Anemia												
Hb normal										1.0		
Hb decreased										0.5	0.1– 3.7	0.462

E

Progression-free survival

	Unadjusted (n=50)			Model 1 (n=50)			Model 2 (n=50)			Model 3 (n=36)		
**Parameter**	**HR**	**95% CI**	***P***	**HR**	**95% CI**	***P***	**HR**	**95% CI**	***P***	**HR**	**95% CI**	***P***
Methylation status												
NC–NB	1.0			1.0			1.0			1.0		
NC–LB	1.7	0.4– 6.6	0.475	2.2	0.5– 10.1	0.303	5.9	1.0– 35.7	0.055	3.2	0.4– 25.6	0.281
HC–LB	16.2	2.4–108.1	0.004	17.3	2.1–139.7	0.008	54.3	4.2–694.9	0.002	56.7	2.7–1194.4	0.009
Age												
Age (yrs)				1.03	0.98–1.09	0.267	1.04	0.98–1.10	0.189	1.03	0.94–1.12	0.533
Gender												
Female				1.0			1.0			1.0		
Male				0.4	0.1– 1.7	0.204	0.1	0.0– 0.9	0.038	0.1	0.0– 1.3	0.081
Pathological stage												
I/II							1.0			1.0		
III/IV							12.2	2.2– 68.9	0.005	6.6	1.0– 41.8	0.046
Anemia												
Hb normal										1.0		
Hb decreased										0.7	0.1– 5.1	0.721

Supplementary Figure S36

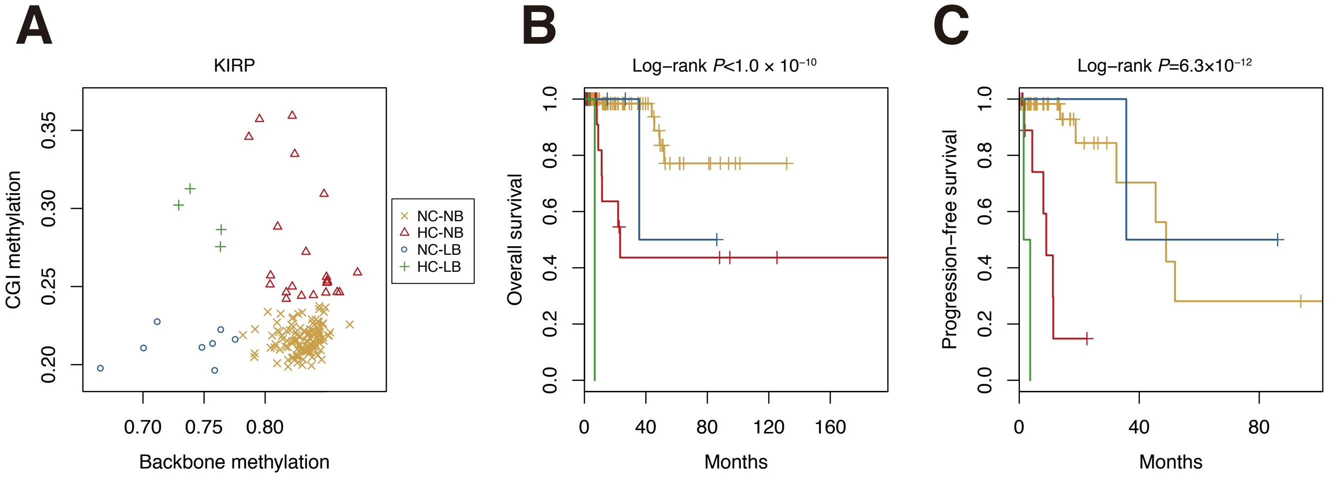

D

Overall survival

Parameter	Unadjusted HR	95% CI	*P*	Model 1 HR	95% CI	*P*	Model 2 HR	95% CI	*P*	Model 3 HR	95% CI	*P*
Methylation status												
NC–NB	1.0			1.0			1.0			1.0		
HC–NB	6.8	2.1–22.6	0.002	6.9	2.1–22.7	0.002	3.0	0.7–14.1	0.154	3.6	0.6–21.6	0.167
NC–LB	3.0	0.4–25.9	0.314	3.3	0.4–31.0	0.297	3.3	0.4–30.2	0.294	0.0	0.0– Inf	0.999
Age												
Age (yrs)				1.00	0.95–1.05	0.922	1.01	0.96–1.06	0.817	1.01	0.94–1.08	0.762
Gender												
Female				1.0			1.0			1.0		
Male				0.7	0.2– 2.5	0.630	0.7	0.2– 2.5	0.601	1.0	0.2– 3.8	0.943
Pathological stage												
I							1.0			1.0		
II/III							1.9	0.3–10.1	0.463	1.4	0.2–13.4	0.753
IV							8.0	1.2–54.6	0.033	6.7	0.7–67.2	0.105
Anemia												
Anemia Absent										1.0		
Anemia Present										1.2	0.3– 5.1	0.764

E

Progression-free survival

Parameter	Unadjusted HR	95% CI	*P*	Model 1 HR	95% CI	*P*	Model 2 HR	95% CI	*P*	Model 3 HR	95% CI	*P*
Methylation status												
NC–NB	1.0			1.0			1.0			1.0		
HC–NB	15.8	3.8–66.0	<0.001	18.1	4.1–80.7	<0.001	4.2	0.6– 30.5	0.155	10.2	0.6– 171.0	0.106
NC–LB	0.7	0.1– 5.5	0.694	0.9	0.1– 8.5	0.921	0.8	0.1– 6.9	0.805			
Age												
Age (yrs)				0.98	0.94–1.03	0.442	0.98	0.92–1.03	0.387	0.92	0.84–1.02	0.117
Gender												
Female				1.0			1.0			1.0		
Male				0.9	0.3– 3.3	0.905	0.9	0.2– 3.6	0.862	12.8	0.7– 241.6	0.089
Pathological stage												
I							1.0			1.0		
II/III							3.9	0.9– 18.1	0.077	101.8	1.1– 9487.8	0.046
IV							18.6	1.7–200.7	0.016	383.0	2.0–73853.3	0.027
Anemia												
Anemia Absent										1.0		
Anemia Present										9.8	0.8– 126.9	0.080

Supplementary Figure S37

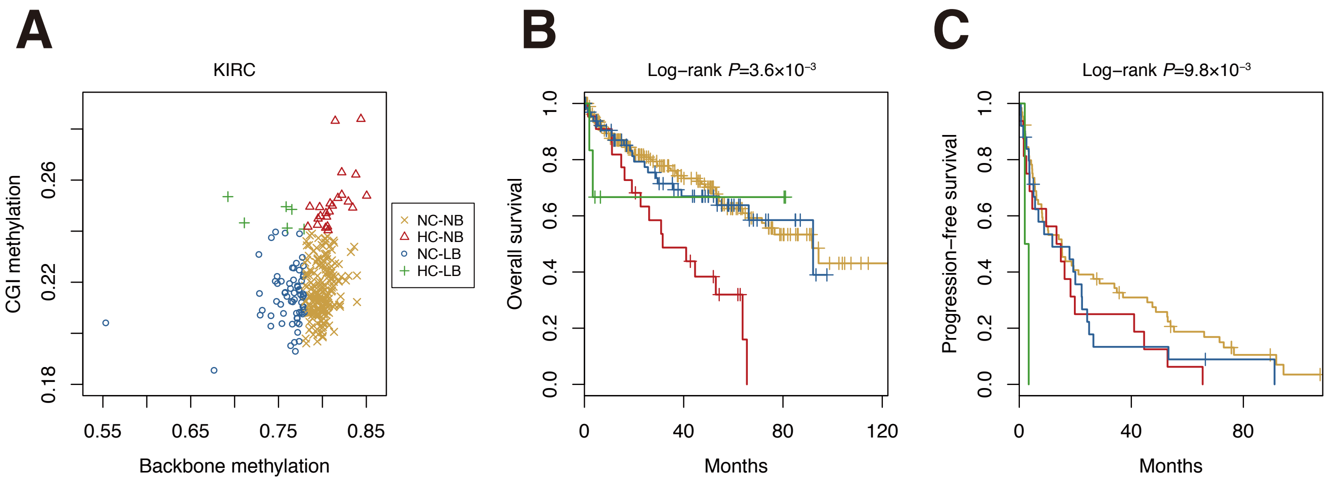

D

Overall survival

	Unadjusted			Model 1			Model 2			Model 3		
**Parameter**	**HR**	**95% CI**	***P***	**HR**	**95% CI**	***P***	**HR**	**95% CI**	***P***	**HR**	**95% CI**	***P***
Methylation status												
NC–NB	1.0			1.0			1.0			1.0		
HC–NB	2.7	1.5–4.7	0.001	2.5	1.4–4.3	0.002	1.2	0.6– 2.3	0.660	0.9	0.5– 1.9	0.863
NC–LB	1.1	0.6–1.8	0.800	1.1	0.7–1.8	0.678	0.8	0.5– 1.5	0.542	0.7	0.4– 1.3	0.224
HC–LB	1.4	0.3–5.8	0.634	1.5	0.4–6.1	0.591	1.9	0.4– 8.8	0.408	0.8	0.1– 7.2	0.875
Age												
Age (yrs)				1.03	1.01–1.05	0.002	1.03	1.01–1.05	0.006	1.03	1.01–1.06	0.005
Gender												
Female				1.0			1.0			1.0		
Male				1.0	0.7–1.6	0.885	0.8	0.5– 1.3	0.381	0.7	0.4– 1.2	0.230
Pathological stage												
I							1.0			1.0		
II							1.3	0.5– 3.4	0.654	1.7	0.6– 5.2	0.334
III							3.2	1.7– 6.0	<0.001	3.2	1.6– 6.2	0.001
IV							8.1	4.3–15.2	<0.001	9.7	4.7–19.8	<0.001
Anemia												
Anemia Absent							1.0			1.0		
Anemia Present							1.5	0.9– 2.5	0.137	1.7	1.0– 3.0	0.048
Thrombocytosis												
Thrombocytosis Absent							1.0			1.0		
Thrombocytosis Present							2.2	1.1– 4.2	0.023	1.8	0.8– 3.9	0.127
VHL mutation												
Absent										1.0		
Present										0.8	0.5– 1.3	0.475

E

Progression-free survival

	Unadjusted			Model 1			Model 2			Model 3		
**Parameter**	**HR**	**95% CI**	***P***	**HR**	**95% CI**	***P***	**HR**	**95% CI**	***P***	**HR**	**95% CI**	***P***
Methylation status												
NC–NB	1.0			1.0			1.0			1.0		
HC–NB	1.5	0.9– 2.7	0.129	1.6	0.9– 2.8	0.102	1.2	0.6– 2.4	0.542	1.4	0.7– 2.9	0.316
NC–LB	1.3	0.8– 2.2	0.238	1.4	0.8– 2.3	0.234	0.9	0.5– 1.6	0.762	0.8	0.4– 1.5	0.444
HC–LB	7.6	1.7–33.2	0.007	7.0	1.6–30.9	0.010	3.6	0.7–17.8	0.110	9.3	1.0–83.3	0.047
Age												
Age (yrs)				1.00	0.98–1.01	0.615	0.98	0.96–1.01	0.150	0.98	0.96–1.01	0.135
Gender												
Female				1.0			1.0			1.0		
Male				1.2	0.8– 1.9	0.372	0.9	0.5– 1.5	0.699	0.9	0.5– 1.5	0.670
Pathological stage												
I							1.0			1.0		
II							1.6	0.6– 4.3	0.396	3.2	1.0–10.6	0.060
III							5.0	2.2–11.2	<0.001	5.1	2.1–12.6	<0.001
IV							5.0	2.4–10.8	<0.001	5.1	2.1–12.3	<0.001
Anemia												
Anemia Absent							1.0			1.0		
Anemia Present							0.7	0.4– 1.2	0.231	0.7	0.4– 1.3	0.248
Thrombocytosis												
Thrombocytosis Absent							1.0			1.0		
Thrombocytosis Present							1.5	0.7– 2.9	0.283	1.7	0.8– 3.6	0.191
VHL mutation												
Absent										1.0		
Present										0.6	0.3– 1.1	0.077

Supplementary Figure S38

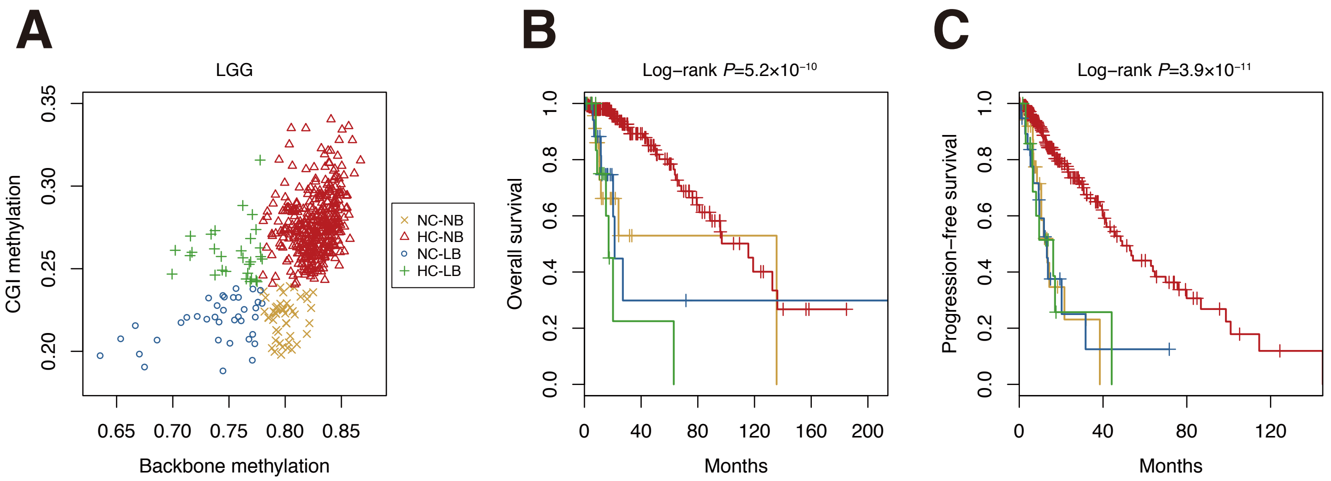

D

Overall survival

	Unadjusted			Model 1			Model 2			Model 3		
**Parameter**	**HR**	**95% CI**	***P***	**HR**	**95% CI**	***P***	**HR**	**95% CI**	***P***	**HR**	**95% CI**	***P***
Methylation status												
HC–NB	1.0			1.0			1.0			1.0		
NC–NB	3.4	1.8– 6.5	<0.001	1.9	0.9– 3.8	0.078	1.8	0.9–3.8	0.115	1.5	0.5–4.7	0.512
NC–LB	3.6	1.6– 8.2	0.003	2.0	0.8– 4.9	0.114	1.3	0.5–3.3	0.558	1.5	0.3–7.0	0.628
HC–LB	10.0	4.2–23.6	<0.001	6.0	2.4–14.7	<0.001	2.8	1.1–7.1	0.036	2.0	0.5–8.7	0.336
Age												
Age (yrs)				1.07	1.05–1.10	<0.001	1.08	1.06–1.11	<0.001	1.07	1.04–1.10	<0.001
Gender												
Female				1.0			1.0			1.0		
Male				0.8	0.5– 1.5	0.522	0.7	0.4–1.3	0.308	0.6	0.3–1.2	0.128
Histological type												
Oligodendroglioma							1.0			1.0		
Oligoastrocytoma							1.2	0.6–2.5	0.664	2.1	0.8–5.3	0.126
Astrocytoma							2.3	1.1–4.5	0.023	3.5	1.4–8.9	0.009
Histological grade												
Grade 2							1.0			1.0		
Grade 3							2.3	1.2–4.3	0.014	1.9	0.9–4.0	0.114
IDH1/2 mutation												
IDH1/2 Absent										1.0		
IDH1/2 Present										0.3	0.1–1.1	0.081
TP53 mutation												
TP53 Absent										1.0		
TP53 Present										1.3	0.4–4.4	0.621
1p19q codeletion												
1p19q Absent										1.0		
1p19q Present										0.8	0.2–2.9	0.694
TERT												
TERT Absent										1.0		
TERT Present										1.4	0.4–4.4	0.563

E

Progression-free survival

	Unadjusted			Model 1			Model 2			Model 3		
**Parameter**	**HR**	**95% CI**	***P***	**HR**	**95% CI**	***P***	**HR**	**95% CI**	***P***	**HR**	**95% CI**	***P***
Methylation status												
HC–NB	1.0			1.0			1.0			1.0		
NC–NB	2.3	1.3–3.8	0.002	1.8	1.0–3.3	0.035	1.9	1.1–3.3	0.033	1.5	0.7–3.4	0.302
NC–LB	3.8	2.0–7.0	<0.001	3.0	1.5–5.9	0.001	2.7	1.4–5.3	0.004	2.2	0.7–6.7	0.162
HC–LB	4.5	2.2–9.0	<0.001	3.7	1.7–7.7	0.001	2.6	1.2–5.6	0.016	2.5	0.9–7.3	0.087
Age												
Age (yrs)				1.02	1.00–1.03	0.069	1.02	1.00–1.04	0.025	1.02	1.01–1.04	0.013
Gender												
Female				1.0			1.0			1.0		
Male				0.9	0.6–1.3	0.556	0.8	0.6–1.3	0.378	0.7	0.4–1.1	0.162
Histological type												
Oligodendroglioma							1.0			1.0		
Oligoastrocytoma							1.0	0.6–1.6	0.895	1.1	0.6–2.2	0.687
Astrocytoma							1.8	1.1–2.9	0.030	1.5	0.8–3.0	0.227
Histological grade												
Grade 2							1.0			1.0		
Grade 3							1.2	0.8–1.8	0.458	0.9	0.6–1.6	0.840
IDH1/2 mutation												
IDH1/2 Absent										1.0		
IDH1/2 Present										0.5	0.2–1.2	0.105
TP53 mutation												
TP53 Absent										1.0		
TP53 Present										0.8	0.3–2.1	0.592
1p19q codeletion												
1p19q Absent										1.0		
1p19q Present										0.8	0.3–2.4	0.685
TERT												
TERT Absent										1.0		
TERT Present										0.5	0.2–1.3	0.163

Supplementary Figure S39

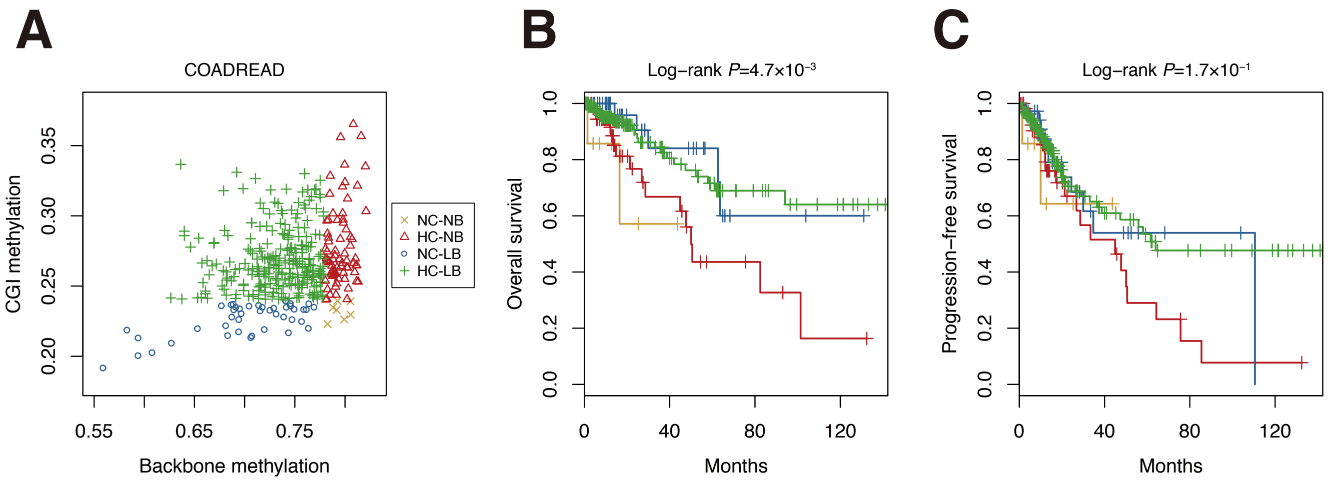

D

Overall survival

	Unadjusted			Model 1			Model 2			Model 3		
**Parameter**	**HR**	**95% CI**	***P***	**HR**	**95% CI**	***P***	**HR**	**95% CI**	***P***	**HR**	**95% CI**	***P***
Methylation status												
NC–NB	1.0			1.0			1.0			1.0		
HC–NB	0.6	0.1–2.7	0.527	0.5	0.1–2.2	0.362	0.4	0.1– 1.8	0.217	0.3	0.0– 3.2	0.331
NC–LB	0.2	0.0–1.1	0.060	0.2	0.0–1.3	0.093	0.1	0.0– 0.8	0.026	0.1	0.0– 1.5	0.097
HC–LB	0.2	0.1–1.1	0.059	0.2	0.1–1.0	0.044	0.1	0.0– 0.7	0.013	0.1	0.0– 1.2	0.075
Age												
Age (yrs)				1.02	0.99–1.04	0.228	1.03	1.00–1.06	0.046	1.04	1.01–1.07	0.016
Gender												
Female				1.0			1.0			1.0		
Male				1.6	0.9–2.9	0.132	1.5	0.8– 2.9	0.243	1.6	0.8– 3.3	0.164
Pathological stage												
I							1.0			1.0		
II							1.5	0.3– 6.8	0.600	1.6	0.4– 7.2	0.542
III							2.5	0.6–11.4	0.229	2.1	0.4– 9.5	0.359
IV							10.3	2.3–46.3	0.002	9.0	2.0–41.5	0.005
Microsatellite instability (MSI)												
MSS/MSI–L										1.0		
MSI–H										0.3	0.1– 1.3	0.103

E

Progression-free survival

	Unadjusted			Model 1			Model 2			Model 3		
**Parameter**	**HR**	**95% CI**	***P***	**HR**	**95% CI**	***P***	**HR**	**95% CI**	***P***	**HR**	**95% CI**	***P***
Methylation status												
NC–NB	1.0			1.0			1.0			1.0		
HC–NB	1.0	0.2–4.3	0.999	0.9	0.2–3.8	0.877	0.8	0.2– 3.4	0.732	1.0	0.1– 8.2	0.991
NC–LB	0.6	0.1–2.8	0.535	0.6	0.1–2.7	0.504	0.4	0.1– 1.9	0.256	0.6	0.1– 5.0	0.600
HC–LB	0.6	0.1–2.4	0.445	0.5	0.1–2.2	0.386	0.4	0.1– 1.7	0.215	0.5	0.1– 4.2	0.538
Age												
Age (yrs)				1.00	0.98–1.02	0.981	1.01	0.99–1.03	0.478	1.01	0.99–1.03	0.287
Gender												
Female				1.0			1.0			1.0		
Male				1.4	0.9–2.1	0.189	1.1	0.7– 1.9	0.580	1.2	0.7– 1.9	0.553
Pathological stage												
I							1.0			1.0		
II							2.1	0.6– 7.1	0.234	2.1	0.6– 7.0	0.240
III							3.2	1.0–10.8	0.057	2.8	0.8– 9.5	0.101
IV							13.1	3.9–44.3	<0.001	11.5	3.3–39.6	<0.001
Microsatellite instability (MSI)												
MSS/MSI–L										1.0		
MSI–H										0.5	0.2– 1.3	0.183

Supplementary Figure S40

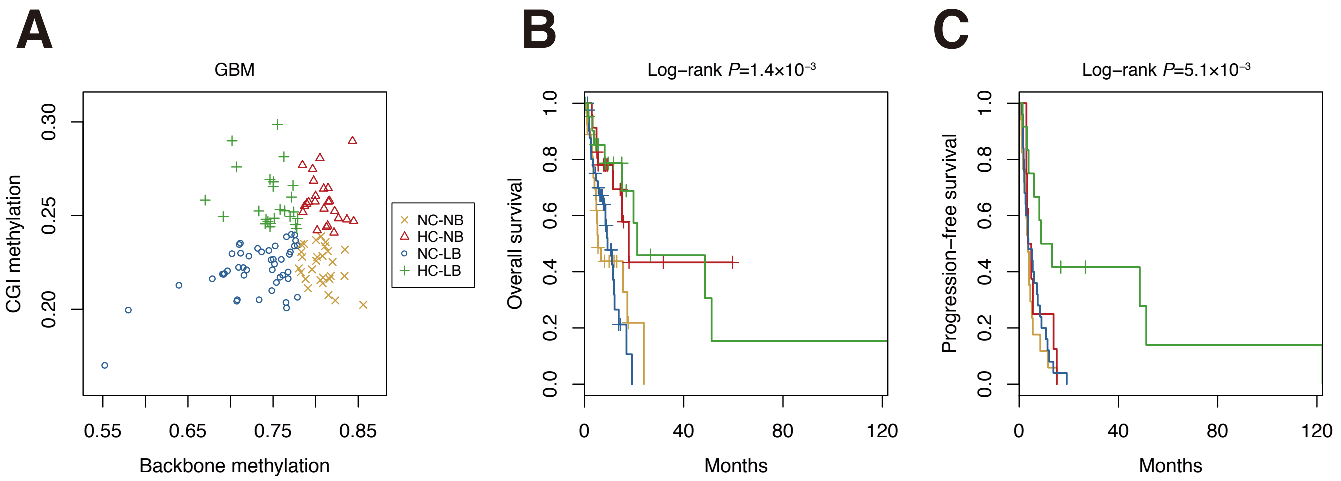

D

Overall survival

Parameter	Unadjusted HR	Unadjusted 95% CI	Unadjusted *P*	Model 1 HR	Model 1 95% CI	Model 1 *P*	Model 2 HR	Model 2 95% CI	Model 2 *P*	Model 3 HR	Model 3 95% CI	Model 3 *P*
Methylation status												
HC–NB	1.0			1.0			1.0			1.0		
NC–NB	3.0	1.3–7.1	0.011	2.8	1.2–6.7	0.019	1.7	0.6–5.0	0.308	5.1	0.2–124.9	0.316
NC–LB	3.2	1.4–7.2	0.006	2.7	1.2–6.3	0.019	2.0	0.7–5.1	0.171	0.9	0.1– 9.5	0.900
HC–LB	0.9	0.3–2.4	0.820	0.9	0.3–2.3	0.768	0.7	0.2–2.5	0.582	0.0	0.0– Inf	0.998
Age												
Age (yrs)				1.05	1.02–1.08	<0.001	1.06	1.02–1.10	0.002	1.11	1.02–1.21	0.018
Gender												
Female				1.0			1.0			1.0		
Male				1.5	0.8–2.5	0.184	0.8	0.4–1.7	0.612	0.1	0.0– 0.6	0.017
Karnofsky score												
>=80							1.0			1.0		
<80							1.3	0.6–2.8	0.562	3.8	0.6– 22.8	0.147
TP53 mutation												
TP53 Absent										1.0		
TP53 Present										1.3	0.3– 5.6	0.738
PTEN mutation												
PTEN Absent										1.0		
PTEN Present										2.5	0.5– 12.5	0.265
EGFR mutation												
EGFR Absent										1.0		
EGFR Present										0.3	0.1– 2.2	0.253
TERT mutation												
TERT Absent										1.0		
TERT Present										15.9	0.5–466.7	0.108

E

Progression-free survival

Parameter	Unadjusted HR	Unadjusted 95% CI	Unadjusted *P*	Model 1 HR	Model 1 95% CI	Model 1 *P*	Model 2 HR	Model 2 95% CI	Model 2 *P*	Model 3 HR	Model 3 95% CI	Model 3 *P*
Methylation status												
HC–NB	1.0			1.0			1.0			1.0		
NC–NB	1.5	0.6–3.5	0.344	1.6	0.7–3.8	0.294	1.2	0.5–3.3	0.694	6.0	0.2–239.4	0.340
NC–LB	1.2	0.5–2.7	0.672	1.1	0.5–2.6	0.807	0.9	0.3–2.3	0.809	1.0	0.1– 8.9	0.985
HC–LB	0.3	0.1–0.9	0.034	0.4	0.1–1.2	0.102	0.3	0.1–1.4	0.125	0.0	0.0– Inf	0.998
Age												
Age (yrs)				1.02	0.99–1.04	0.178	1.02	0.98–1.06	0.457	1.03	0.94–1.11	0.562
Gender												
Female				1.0			1.0			1.0		
Male				1.0	0.5–1.8	0.955	0.5	0.2–1.2	0.124	0.2	0.0– 1.9	0.169
Karnofsky score												
>=80							1.0			1.0		
<80							1.3	0.6–2.8	0.540	1.2	0.1– 9.4	0.872
TP53 mutation												
TP53 Absent										1.0		
TP53 Present										2.7	0.5– 16.0	0.277
PTEN mutation												
PTEN Absent										1.0		
PTEN Present										3.9	0.6– 23.7	0.136
EGFR mutation												
EGFR Absent										1.0		
EGFR Present										0.6	0.1– 3.6	0.552
TERT mutation												
TERT Absent										1.0		
TERT Present										2.2	0.2– 19.3	0.479

Supplementary Figure S41

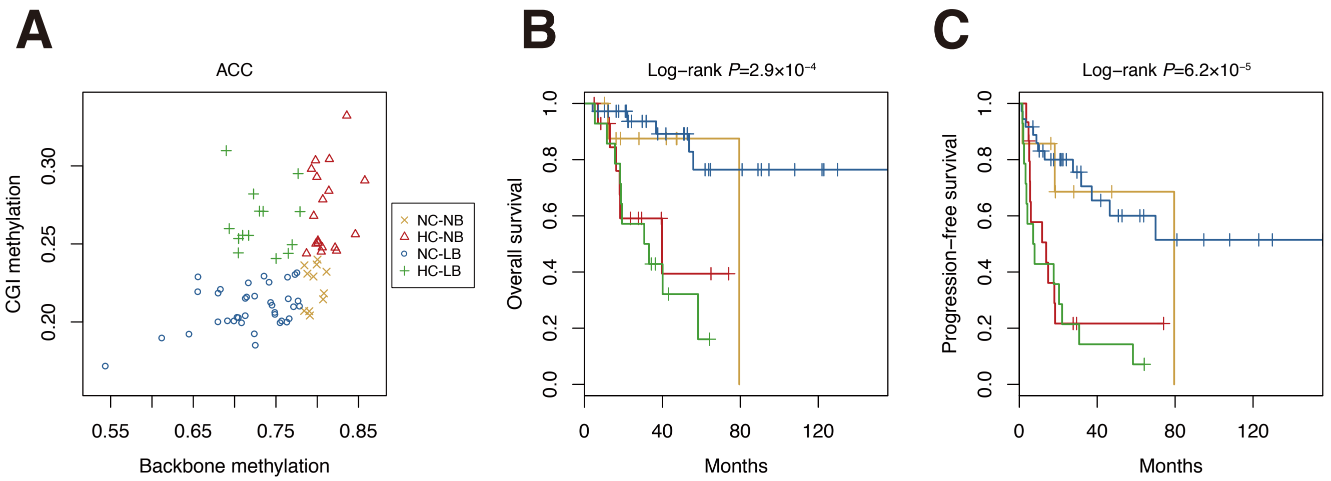

D

Overall survival

	Unadjusted			Model 1			Model 2			Model 3		
**Parameter**	**HR**	**95% CI**	***P***	**HR**	**95% CI**	***P***	**HR**	**95% CI**	***P***	**HR**	**95% CI**	***P***
Methylation status												
NC–NB	1.0			1.0			1.0			1.0		
HC–NB	2.5	0.5–12.7	0.265	2.8	0.5–14.7	0.214	2.1	0.4–11.2	0.405	3.6	0.6–21.2	0.152
NC–LB	0.4	0.1– 2.2	0.305	0.4	0.1– 2.3	0.337	0.3	0.1– 1.8	0.196	0.5	0.1– 3.0	0.486
HC–LB	3.5	0.7–16.2	0.111	4.1	0.8–19.8	0.081	2.2	0.4–11.2	0.351	5.0	0.9–27.9	0.066
Age												
Age (yrs)				1.00	0.97–1.02	0.831	1.01	0.98–1.04	0.649	1.00	0.96–1.03	0.908
Gender												
Female				1.0			1.0			1.0		
Male				0.7	0.3– 1.7	0.433	0.9	0.3– 2.3	0.760	1.1	0.4– 3.0	0.905
Pathological stage												
I/II							1.0			1.0		
III/IV							6.1	1.9–19.5	0.002	3.3	1.0–11.3	0.058
TP53 mutation												
TP53 Absent										1.0		
TP53 Present										4.1	1.5–11.8	0.008

E

Progression-free survival

	Unadjusted			Model 1			Model 2			Model 3		
**Parameter**	**HR**	**95% CI**	***P***	**HR**	**95% CI**	***P***	**HR**	**95% CI**	***P***	**HR**	**95% CI**	***P***
Methylation status												
NC–NB	1.0			1.0			1.0			1.0		
HC–NB	2.8	0.8–10.2	0.117	3.9	1.0–14.8	0.042	2.9	0.7–11.3	0.124	2.9	0.8–11.4	0.121
NC–LB	0.7	0.2– 2.5	0.580	0.8	0.2– 2.8	0.723	0.7	0.2– 2.4	0.546	0.7	0.2– 2.6	0.591
HC–LB	3.6	1.0–12.9	0.047	5.3	1.4–19.6	0.013	3.2	0.8–12.9	0.097	3.3	0.8–13.1	0.092
Age												
Age (yrs)				0.99	0.97–1.02	0.633	0.99	0.97–1.02	0.566	0.99	0.97–1.02	0.521
Gender												
Female				1.0			1.0			1.0		
Male				0.4	0.2– 0.9	0.028	0.5	0.2– 1.1	0.072	0.5	0.2– 1.1	0.095
Pathological stage												
I/II							1.0			1.0		
III/IV							3.7	1.7– 7.9	0.001	3.5	1.6– 8.0	0.003
TP53 mutation												
TP53 Absent										1.0		
TP53 Present										1.1	0.5– 2.5	0.867
